# NIR‐Sensitized Activated Formation of Lophyl Radicals by Heptamethine Cyanines Enables Dry Film Photoresists

**DOI:** 10.1002/anie.202422700

**Published:** 2025-04-03

**Authors:** Xianglong He, Yayu Shao, Weifeng Ma, Xun Sun, Zehao Jin, Yulian Pang, Yangyang Xin, Yingquan Zou, Bernd Strehmel

**Affiliations:** ^1^ College of Chemistry Beijing Normal University No. 19, Xinjekouwai St., Haidian District Beijing 100875 P.R. China; ^2^ Key Lab of Organic Optoelectronics and Molecular Engineering of Ministry of Education Department of Chemistry Tsinghua University No. 30, Shuangqing Rd., Haidian District Beijing 100084 P.R. China; ^3^ College of Chemistry Beijing University of Chemical Technology Beijing 100029 P.R. China; ^4^ Hubei Gurun Technologies Co., Ltd. Jingmen Chemical Recycling Industrial Park Jingmen Hubei Province 448000 P.R. China; ^5^ Institute for Coatings and Surface Technology Niederrhein University of Applied Sciences Adlerstr. 1 47798 Krefeld Germany

**Keywords:** Hexaarylbisimidazole, Near infrared, Photoinduced electron transfer, Radical polymerization, Resist

## Abstract

The near‐infrared (**NIR**) sensitized generation of lophyl radicals (**L·**) by heptamethine cyanine led to initiation of radical photopolymerization of multifunctional acrylates when different hexa‐arylbisimidazoles (**HABI**s) and *N*‐phenylglycine (**NPG**) operated as coinitiator. The latter functioned as donor. For a deeper understanding, heptamethine cyanines were used following a photoinduced electron transfer (**PET**). **HABI** derivatives with electron‐donating and ‐withdrawing substituents demonstrated that those with electron acceptors resulted in a higher photopolymerization efficiency of multifunctional acrylates. Tri‐(propylene glycol) diacrylate (**TPGDA**) and tri‐methylolpropane triacrylate (**TMPTA**) served as the monomers. Sensitizers (**Sens**) exposed with a high intense NIR‐light source at 808 nm exhibiting a positive charge in the cyanine pattern significantly operate more efficiently for radical photopolymerization than a **Sens** without a positive charge. Differences in efficiency of **PET** can give an explanation for these differences. The heat generated by the cyanine's internal conversion from S_1_ to S_0_ additionally influenced the endothermic reaction between **L·** and **NPG**. These systems worked in practical applications for dry film photoresists (**DFR**s), reported here for the first time.

## Graphical abstract

High‐intensity NIR radiation around 800 nm enables the manufacture of dry film resists (**DFR**s) using a combination of hexa‐arylbisimidazole (HABI) and *N*‐phenyl imino acetic acid. The mechanism is based on activated electron transfer processes supported by the respective product analysis.

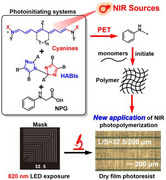

## Introduction

Nowadays, the electronics industry demands the development of materials with improved resolution.^[^ ^1^, ^2^, ^3^, ^4^, ^5^, ^6^, ^7^, ^8^, ^9^, ^10^, ^11^, ^12^, ^13^ ^]^ At the same time, introducing cheaper and more efficient production technologies requires advancements in patterning technologies, which also necessitates more focus on resist technologies.^[^ ^12^, ^13^, ^14^, ^15^, ^16^ ^]^ This addresses the development of respective light‐sensitive materials combined with efficient radiation sources. The use of mask techniques also connects to the manufacture of circuit boards with a broader resolution, which is also needed for electronic mass goods^[^ ^10^ ^]^ including high‐throughput technologies.^[^ ^13^ ^]^ This becomes more economical in combination with light emitting diode (LED) techniques. Ultraviolet (UV)‐LED have received great development^[^ ^17^, ^18^ ^]^ but these short wavelengths possess higher scattering coefficients compared to those emitting in the near infrared (NIR) region.^[^ ^19^, ^20^ ^]^

The development of affordable and easy‐to‐handle NIR sources, such as high‐power NIR LED, lacked sufficient progress for many years until the first high‐power NIR LED was introduced for use in conventional radical photopolymerization in late 2018.^[^ ^21^ ^]^ Here, the heat released by a NIR **Sens** enabled to overcome the internal activation barrier of a photoinduced electron transfer (**PET**),^[^ ^21^, ^22^, ^23^, ^24^ ^]^ which additionally led to the development of NIR‐sensitized cationic polymerization.^[^ ^21^, ^23^, ^24^, ^25^, ^26^, ^27^ ^]^ These systems benefit from less scattering of NIR photons because of the fewer scattering coefficients than UV photons.^[^ ^19^, ^20^ ^]^ Nevertheless, they comprise iodonium salts as coinitiators, which connect to several toxicological issues. This relates particularly to the anions. Here, hexafluoro phosphates (PF_6_^−^) were disclosed to release HF under certain circumstances.^[^ ^28^, ^29^ ^]^ Alternative anions, such as the bis(trifluomethanesulfonyl) imide^[^ ^30^ ^]^ [(CF_3_SO_2_)N]^−^ or tetra(nonafluoro‐*t*‐butoxy)aluminate^[^ ^25^, ^31^ ^]^ [(*t*‐C_4_F_9_‐O)_4_Al]^−^, fit under the definition of polyfluoroalkyl substances (**PFAS**).^[^ ^32^, ^33^ ^]^ **PFAS** chemicals have received many concerns, and according to regulations they may be mostly banned for use in the future.^[^ ^32^ ^]^ Here, a demand exists to develop alternatives demonstrated in this contribution in the case of **DFR**s.

The disclosed systems could also be used for other applications based on direct laser writing (**DLW**).^[^ ^1^, ^4^, ^7^, ^9^ ^]^ This technique has gained significant interest in processing metal oxides, carbon‐based materials, depositing precursors, and other applications nanomaterials.^[^ ^1^ ^]^ It needed only a few years after the introduction of the laser^[^ ^34^ ^]^ to introduce the first report of laser machining to generate thin films and integrated circuits.^[^ ^35^ ^]^ The development also focused on the writing of memory cards^[^ ^36^ ^]^ and other devices for the electronic industry.^[^ ^1^ ^]^ Presumably, the system disclosed here may also find use in some of those mentioned applications as well.

Light‐mediated conventional radical polymerization meets the requirements of **DFR**s as shown for a UV‐LED system recently.^[^ ^37^ ^]^ Nevertheless, such systems lack in some circumstances regarding the resolution caused by scattering as discussed *vide supra*. In addition, it opens the possibility to use light‐insensitive matrix materials or additives with UV absorption, which typically filter UV light, explaining the growing interest in these technologies.^[^ ^38^ ^]^ The design of such a system requires a **Sens** that absorbs **NIR** radiation between 750 and 900 nm, and a coinitiator to initiate the crosslinking of multifunctional acrylic esters. The latter should not connect to the issues of HF release and PFAS.

Although **HABI** were disclosed to work in UV/blue light‐sensitized systems, their use in NIR‐sensitized systems failed.^[^ ^39^, ^40^, ^41^ ^]^ There, a third component operated as a coinitiator, typically a heterocyclic mercapto compound.^[^ ^39^, ^40^ ^]^ A **PET** was believed to transfer an electron to **HABI,** resulting in the formation of the respective lophyl radical (**L·**), which reacts with a mercapto compound, resulting in the generation of the highly reactive thiyl radical.^[^ ^39^, ^40^ ^]^ Hydrogen abstraction was believed to proceed as the main responsible pathway to generate RS**·** for a long time. This must be revised after the release of a report showing that, particularly with mercapto triazole (**MT**), the mechanism appeared more complex.^[^ ^42^ ^]^ The thiol/thion equilibrium might be one main reason because **MT** mainly appears in its tautomeric thion form in acrylic esters.^[^ ^42^ ^]^

Thus, this article provides a new impetus in the field of **DFR**s development using as a modern light source a high‐power NIR‐LED. The NIR‐sensitized generation of initiating radicals proceeds by introducing two coinitiators, namely the **HABI**, and **NPG**. **HABI‐101** (*vide infra*) was not identified as being related to serious toxicological issues. Thus, cytotoxicity of the new materials was checked (Table S1).

## Results and Discussion

### Selection of the System

**Scheme 1:**
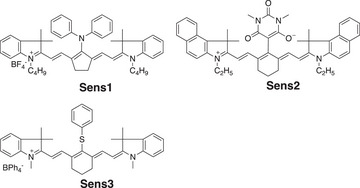
Structures of **Sens1–3**.

The **Sens1**‐**3** (see Scheme 1) operated as **Sens** in a triple system comprising additionally **HABI** (**L**‐**L**), a dimer of two **L·**, and **NPG** as donor. They sufficiently absorb NIR‐radiation at 820 nm. The excited state formed (Sens*) transfers an electron to **HABI** resulting in formation of the respective **L·** and lophine (**LH**). Here, the oxidation potential *E*_ox_ of **Sens**, reduction potential *E*_red_ of **HABI**, and the excitation energy (*E* **_∞_**) of **Sens** tailor the free enthalpy of the **PET**; that is Δ*G*_et_. Equation 1 defines the relation between these parameters.

(1) $$\begin{array}{ccc} & & \Delta G_{\mathrm{et}}=F\times \left(E_{\mathrm{ox}}-E_{\mathrm{red}}\right)-E_{\infty }\\ & & \left(F=\text{Faraday constant}\right) \end{array}$$

**Scheme 2:**
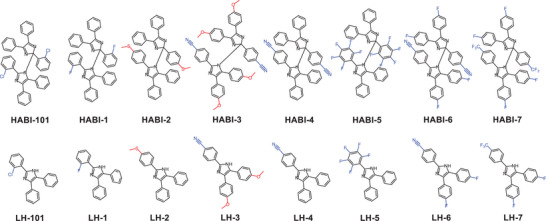
Structures of the commercially available **HABI‐101** and the self‐synthesized **HABI‐1** to **HABI‐7**, as well as their respective precursors **LH‐101**, **LH‐1** to **LH‐7**.

Table 1 shows the data for the reaction between Sens*, and several **HABI** derivatives. Scheme 2 depicts the structural changes of them. **HABI‐101** comprises one chlorine substituent in each imidazolyl group. The change by fluorine in the same position resulted in **HABI‐1**, while the CH_3_‐S group in **HABI‐2** should increase the electron density. **HABI‐3** comprises donor and acceptor groups, which should affect the electron density as well. A comparison between **HABI‐4** and **HABI‐5** would also be interesting because five fluorine atoms on a phenyl moiety cause a similar decrease of electron density as one cyano group.^[^ ^43^ ^]^ This was concluded from electrochemical reduction potentials. In addition, perfluorinated arenes can form π‐stacked structures,^[^ ^44^ ^]^ which might have an additional impact on the reactivity of **L·**. **HABI‐6** and **HABI‐7** bear additional acceptor moieties complementing this series. Table 1 shows the results regarding the electrochemical experiments (Figures S1–S16) *vide infra*.

**Table 1:** Results obtained for optical data (*λ*_max_: absorption maximum, *ε*_max_: extinction coefficient), electrochemical data (*E*_ox_: oxidation potential, *E*_red_: reduction potential, and *ΔG*_et_: reaction enthalpy of **PET** between **Sens** and **HABI**s).^a)^ Supporting Information provides more spectral data of HABIs and Sens (Figure S24).

						Oxidative Mechanism			Reductive Mechanism	**L·** + **NPG**
	*λ*_max_ [nm]^a)^	*ε*_max_ [M^−1^ cm^−1^]^a)^	*ε*_808_ [M^−1^ cm^−1^]^a)^	*E*_red_ [V]^a)^	*E*_ox_ [V]^a)^	*ΔG*_et_ (**Sens1**) [eV]	*ΔG*_et_ (**Sens2**) [eV]	*ΔG*_et_ (**Sens3**) [eV]	*ΔG*_et_(**NPG**)^c)^ [eV]	*ΔG*_et_ ^c)^ [eV]
**HABI‐101**	230	3.295 × 10^4^	0	−0.91	1.47	−0.063	−0.180	−0.040		
**HABI‐1**	230	2.900 × 10^4^	0	−0.91	1.48	−0.067	−0.185	−0.045		
**HABI‐2**	268	3.390 × 10^4^	0	−0.92	1.06	−0.052	−0.170	−0.030		
**HABI‐3**	231	4.787 × 10^4^	0	−0.89	1.27	−0.077	−0.195	−0.055		
**HABI‐4**	232	4.422 × 10^4^	0	−0.90	1.59	−0.076	−0.194	−0.054		
**HABI‐5**	231	3.181 × 10^4^	0	−0.89	1.69	−0.081	−0.199	−0.059		
**HABI‐6**	232	4.992 × 10^4^	0	−0.90	1.58	−0.072	−0.190	−0.050		
**HABI‐7**	230	3.016 × 10^4^	0	−0.89	1.58	−0.081	−0.199	−0.059		
**LH‐1**				−0.90	0.27					0.96
**LH‐2**				−0.44	0.17					0.86
**LH‐3**				−0.87	0.36					1.05
**LH‐4**				−0.79	0.31					1.00
**LH‐5**				−0.81	0.16					0.85
**LH‐6**				−0.77	0.33					1.02
**LH‐7**				−0.96	0.33					0.561
**Sens1**	802	2.55 × 10^5^	2.43 × 10^5^	−0.60^b)^	0.57^b)^				−0.26	
**Sens2**	788	3.56 × 10^5^	1.71 × 10^5^	−0.86^b)^	0.48^b)^				−0.024	
**Sens3**	803	3.34 × 10^5^	3.17 × 10^5^	−0.49^b)^	0.59^b)^				−0.36	

^a)^ In dichloromethane;

^b)^ From Ref. [45];

^c)^ *E*_ox_ for **NPG** = 0.69 V.^[^ ^46^ ^]^

**HABI** derivatives were originally developed as photochromic materials,^[^ ^47^, ^48^, ^49^, ^50^ ^]^ showing a quantitative efficiency for forming **L·** by exposure to UV light. The sterically challenging structure retards the recombination of **L·** to the respective dimer (**L**‐**L**), the hexa‐arylbisimidazole.^[^ ^49^, ^51^, ^52^, ^53^ ^]^ Nevertheless, **L·** adds to vinyl monomers with very low efficiency.^[^ ^42^ ^]^ It exhibits, depending on the structure, a colored appearance even under oxygen. Adding electron‐rich components leads to decolorization and formation of reactive radicals that initiate radical polymerization.^[^ ^42^ ^]^ These reactions allow them to function in *Type II* photoinitiating systems, which require at least two components. However, these systems mostly exhibit a high sensitivity to UV by direct exposure to **L**‐**L**.^[^ ^42^, ^54^ ^]^ At the same time, sensitization was shown to be an effective method for achieving operation with a blue or green light source. This was accomplished by incorporating a sensitizer with the corresponding absorption capacity.^[^ ^40^ ^]^ To our best knowledge, there does not exist any report disclosing successful NIR‐sensitized formation of **L**.

Additional electrochemical data (Figures S17–S23) were taken from the respective lophines. One can conclude from their oxidation potential the respective reduction value in the equilibrium of Equation 2, which is the back reaction. Data obtained can be seen in Table 1 *vide infra*. They fit in the frame of triphenyl imidazolyl radicals.^[^ ^48^, ^55^, ^56^ ^]^

(2) $$\mathrm{LH}⇄L\cdot +H^{+}+e^{-}$$

In this instance, **LH**‐**5** exhibits the lowest *E*_ox_ value within this series, signifying that this species possesses the highest affinity for the electron uptake of **L·**. This observation surprises because **LH**‐**4** comprises a cyano group. It exhibits comparable electron‐withdrawing characteristics as the five fluorine atoms on the phenyl ring in **LH**‐**5**. This is concluded from previous experiments.^[^ ^43^, ^44^, ^57^ ^]^ Furthermore, data of the respective precursors **LH** were taken in electrochemical investigations considering the equilibrium **LH** ⇄ **L·** + e^−^ + **H** ^+^. The forward process represents the oxidation of **LH**, while the back reaction represents the reduction of **L·**.

**Scheme 3:**
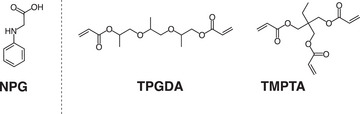
Structures of the **NPG**, monomers **TPGDA** and **TMPTA**.

**NPG**, see Scheme 3, was chosen as an additional coinitiator to generate initiating radicals. This selection bases on several aspects. On the one hand, one may expect H‐abstraction at the CH_2_‐group of the amine by **L·**. On the other hand, the amine can also donate an electron to a species operating as an acceptor, such as the **L·** formed, resulting in the formation of PhNH‐CH_2_ **·** and CO_2_. Experiments pursued can confirm the formation of the latter; see the discussion *vide infra*. Furthermore, the option of electron transfer without sensitization was previously discussed in a series of several **NPG** derivatives substituted with electron‐donating and electron‐withdrawing groups in combination with **HABI**‐**101**.^[^ ^46^ ^]^ Here, the experiments depended on the reactivity of the lophyl radical and *E*_ox_ of **NPG**.

From this point of view, the coinitiator combination comprising **HABI** and **NPG** represents an interesting alternative with less toxicological issues^[^ ^30^ ^]^ compared to an iodonium salt (Table S1), whose PF_6_^−^ anion can release HF under certain circumstances.^[^ ^28^, ^29^ ^]^ Only **HABI‐5** does not fit in this series but it was comparable with bis(*t*‐butyl phenyl)iodonium hexafluorophosphate. The latter has been widely used in practical applications.^[^ ^58^ ^]^ The perfluoro substitution at the arene ring appears undoubtly as one reason although this pattern does not fit to the **PFAS** issue.^[^ ^32^ ^]^

In addition, polymerization experiments were pursued on the crosslinking monomers **TPGDA** and **TMPTA**, which exhibit different functionalities that may affect mobility during network formation.

### NIR‐Sensitized Radical Polymerization

**Figure 1:**
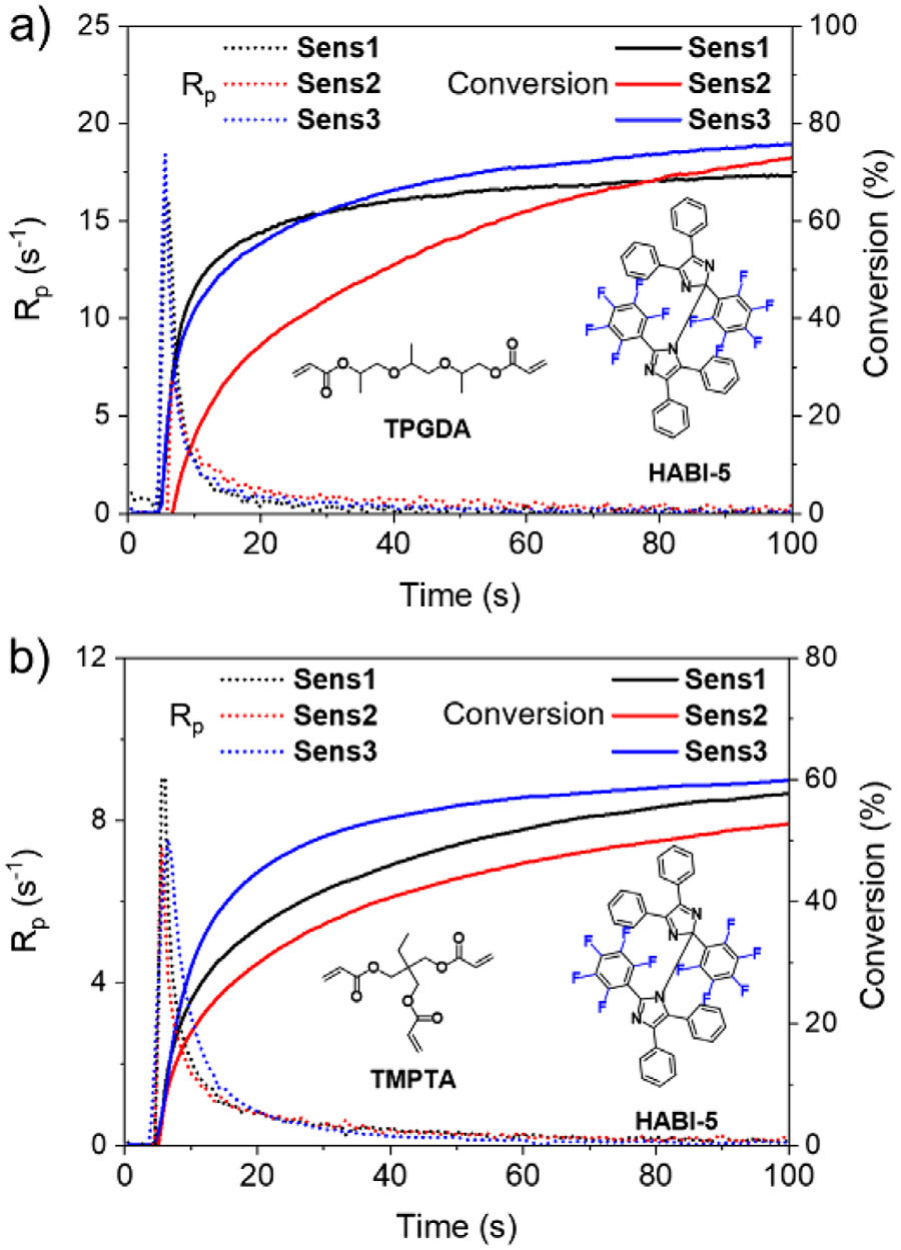
a) Double bond conversion of **TPGDA** and b) *R*_p_ with different **Sens** (0.2 mol% to **TPGDA**) and **HABI‐5** (1 mol% to **TPGDA**) in the presence of the **NPG** (2 mol% to **TPGDA**) applying a NIR laser source emitting at 808 nm with an excitation density of 714 mW·cm^−2^ (see the Supporting Information for more examples).

Figure 1 shows the successful results obtained for photoinduced conventional radical polymerization using a cyanine shown in Scheme 1 as sensitizer together with the coinitiator combination **HABI‐5** and **NPG**. Numeric differentiation of the conversion degree with respect to the reaction time *t* yields the polymerization rate *R*_p_. Thus, the maximum polymerization rate (*R*_p_^max^) and the final conversion (*x*_∞_) represent parameters disclosing the reactivity of a system with a crosslinking mechanism. Supporting Information provides more data in Figures S25–S28 and Tables S2, S3.

**TPGDA** (Figure 1a) indicates an acceptable high *x*_∞_, while the entire polymerization exhibits different profiles. Here, the systems containing **Sens1**, and **Sens3** polymerize faster than that with **Sens2** upon exposure with a high‐intensity NIR source; that is a laser. This can be seen more clearly by comparing the *R*_p_^max^ data in Figure 1b confirming a similar pattern regarding the reactivity of **Sens2**. This was not expected because **Sens2** always responded well to exposure even with low intensity NIR sources.^[^ ^59^, ^60^ ^]^ Thus, additional parameters caused by the different structures of **HABI** must responsibly change the efficiency of radical photopolymerization. However, quantum chemical calculations did not make the situation clearer (Figures S29–S33). One reason can be the large size of the target compounds, which does not facilitate to generate results reliably explaining the scenario.^[^ ^61^, ^62^ ^]^ From a qualitative point of view, these data enable to understand changes in the pattern of the participating molecular orbitals. These calculations were not helpful to understand the scenario regarding the excitation of cyanines (Figure S29), the band gab between **HOMO** and **LUMO** of the **HABI**s in Figure S30, and the findings for the bond dissociation energies in Figure S31. Calculation of the charge distributions and the spin densities gave for all lophyl radicals a similar pattern as shown in Figures S32–S33. This would not help to explain the reactivity differences found. Experiments indicated different behavior.

**Sens1** was disclosed as **PET** system with a higher internal activation barrier if an iodonium salt operated as coinitiator^[^ ^21^, ^23^, ^24^, ^25^, ^45^ ^]^ if a high intensity NIR source served as light source. However, **Sens2** exhibited the best reactivity with the same coinitiator even with a low NIR LED as exposure source. Obviously, change to the coinitiator combination **HABI**/**NPG** results in a different scenario. **Sens2** connects to less photopolymerization efficiency compared to **Sens1** but it also approaches a similar *x*_∞_ in a similar time frame. A different mechanism of the coinitiator combination **HABI**/**NPG** could explain these findings.

**Figure 2:**
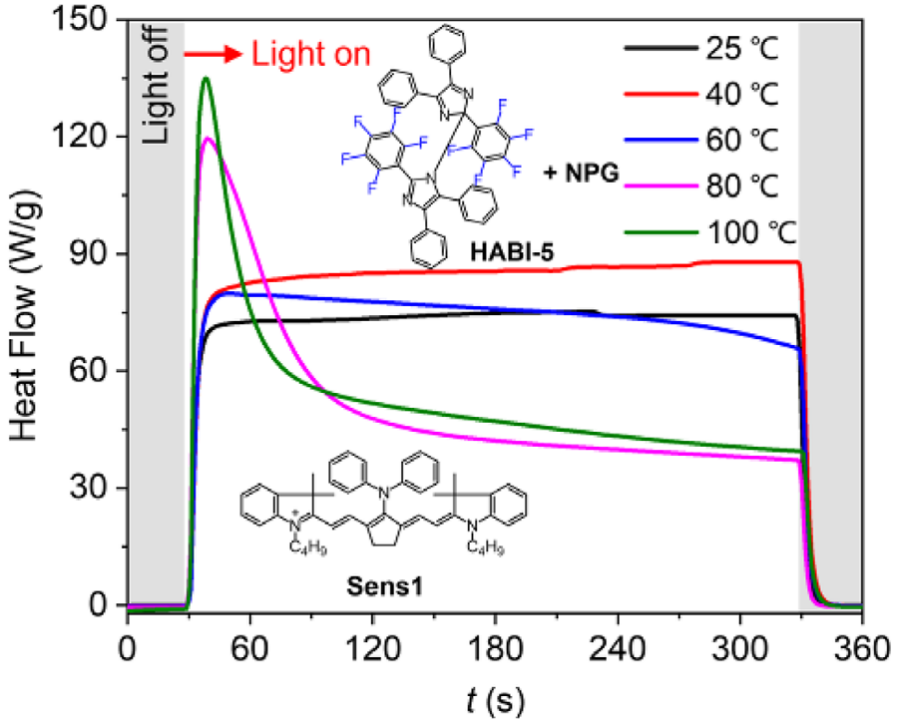
Photo‐DSC curves of the **Sens1**/**HABI‐5**/**NPG**/**TPGDA** system (0.2 mol% for **Sens1,** 1 mol% for **HABI‐5,** and 2 mol% for **NPG** to **TPGDA**) under irradiation at 808 nm with a NIR laser operating with light intensity of 225 mW cm^−2^ at different temperatures. The nonequality between the reference and sample causes the heat flow between 60 and 70 W g^−1^, while nearly no heat generated by polymerization was recorded at this temperature.

**Figure 3:**
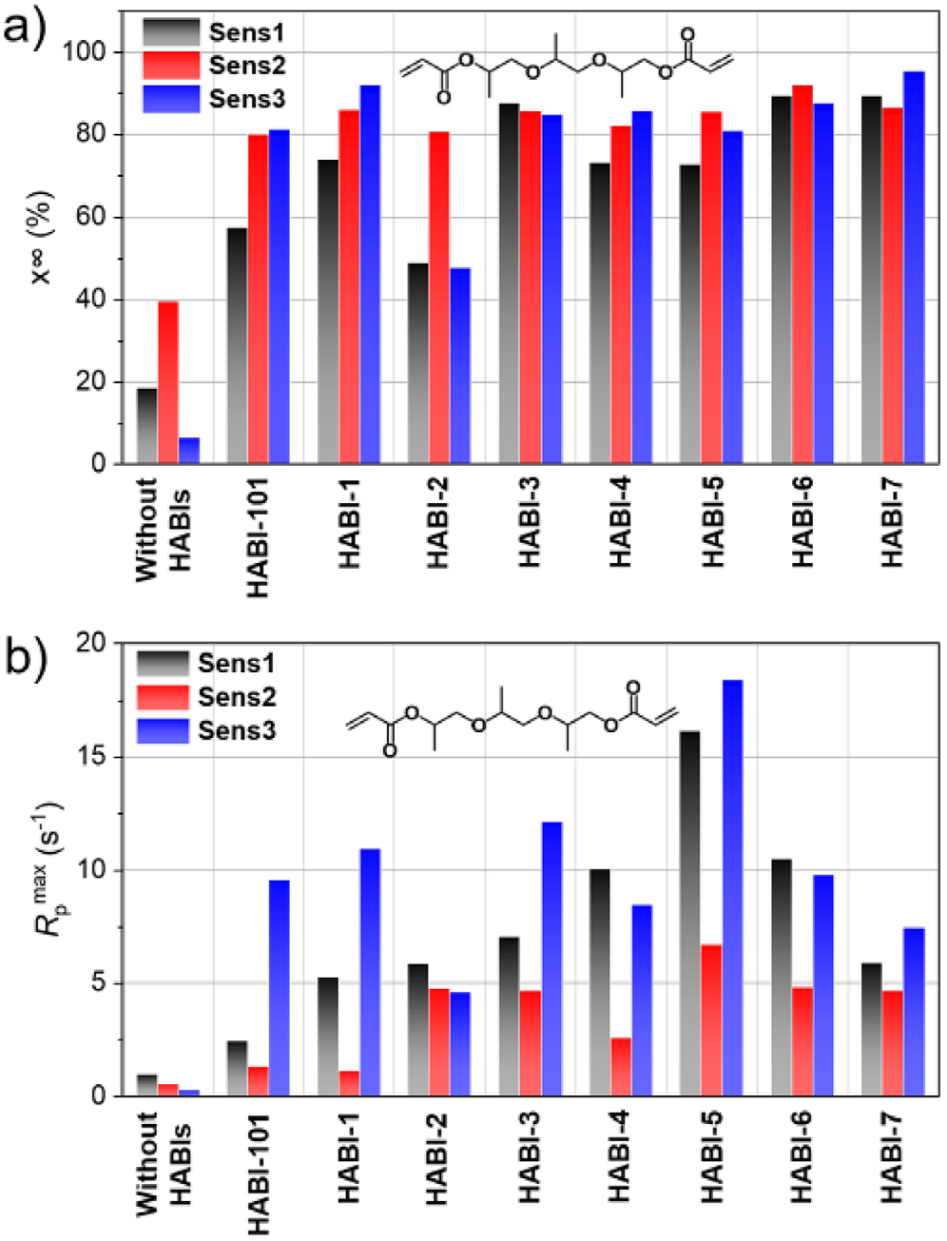
a) *x*_∞_ of **TPGDA** and b) *R*_p_^max^ with different **Sens** (0.2 mol% to **TPGDA**) and **HABI**s (1 mol% to **TPGDA**) in the presence of the **NPG** (2 mol% to **TPGDA**) applying a NIR laser source emitting at 808 nm with an excitation density of 714 mW·cm^−2^ (Exposure time: 5 min).

The mechanism discussed regarding the activated **PET** mentioned *vide surpa* uses photo‐DSC to record the heat flow caused by polymerization at different temperatures, whose setup was previously disclosed.^[^ ^21^, ^24^ ^]^ This provides answers whether the system **Sens1**/**HABI‐5**/**NPG** follows an activated **PET** mechanism. Figure 2 illustrates the results. Exposure at 25°C and 40°C showed nearly no heat evolution and therefore no photopolymerization. Only small increase exists at 60°C, while data obtained at 80°C suddenly increased indicating a threshold system. This did not show up with such a significance compared to iodonium salts although also there the temperature dependence was reported.^[^ ^21^, ^24^ ^]^ An activated **PET** can be seen as possible reason to explain this phenomenon. On the other hand, real‐time FTIR experiments, where this photopolymerization occurred well as shown in Figure 1 *vide supra*, relate to isoperibolic conditions where the remaining heat positively affects the **PET** resulting finally in efficient photopolymerization, Figures 1 and 3.

These results motivated the study of how a variation of the **HABI** pattern affected reactivity continuing with real‐time FTIR experiments. Figure 3 summarizes the results obtained. The omission of **HABI** resulted, as expected, in a very low polymerization rate, no matter which sensitizer operated upon exposure. This rules out that the borate anion in **Sens3** could cause any significant acceleration of polymerization efficiency as reported for previous systems.^[^ ^63^, ^64^, ^65^ ^]^ *R*_p_^max^ exhibited the highest value in the case of **Sens3** combined with **HABI**‐**5,** followed by the remaining **HABI** derivatives on a similar level except for **HABI**‐**2,** which comprises an electron‐donating moiety. This became clearer by replacing **Sens3** by **Sens1**, where **HABI**‐**5** exhibited the highest polymerization rate followed by **HABI**‐**4** and **HABI**‐**6**. These differences surprised because the Δ*G*_et_ for **PET** between Sens* and **HABI** appeared similar for all of them, see Table 1. This shows that the thermodynamic parameters to disclose **PET** do not efficiently describe the reactivity if **HABI** and **NPG** operate as coinitiator. Obviously, the addition of **HABI** derivatives positively affects the photochemical reactivity of the NIR photoinitiator system. These results motivated to continue with exposure studies to understand the proceeding processes in more detail.

### Photochemistry with NIR Exposure of Heptamethine Cyanines in Combination with HABI and NPG

**Figure 4:**
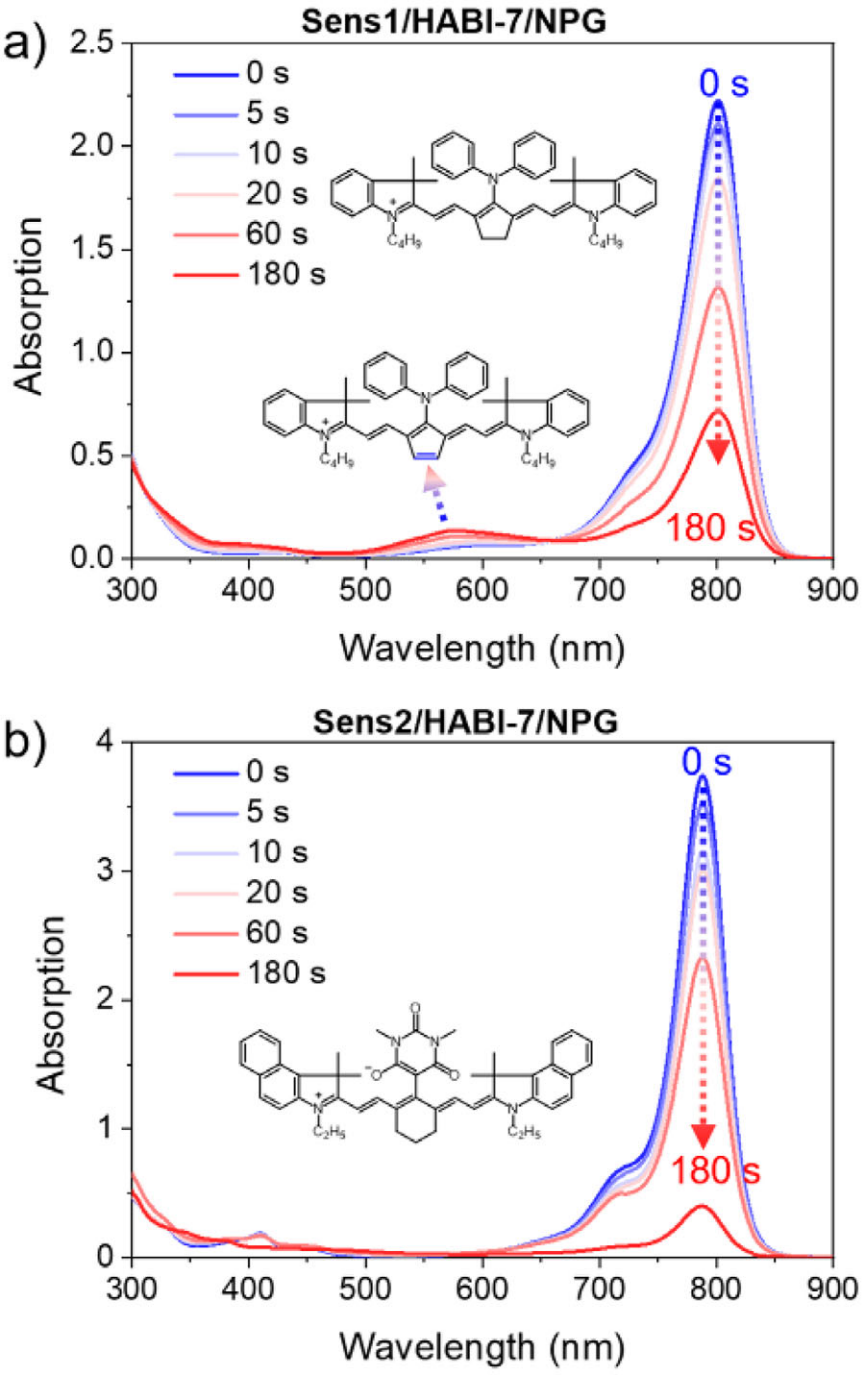
Photolysis curves of a) **Sens1**/**HABI**‐**7**/**NPG** and b) **Sens2**/**HABI**‐**7**/**NPG** systems in anhydrous dichloromethane upon irradiation at 808 nm laser with an exposure density of 714 mW·cm^−2^, (concentration = 1.0 × 10^−5^ M for **Sens**, 2 × 10^−5^ M for **HABI**‐**7,** and 4.0 × 10^−5^ M for **NPG**). Experiments were pursued under nitrogen.

Figure 4 shows the change in the absorption spectra of the respective cyanine in combination with **HABI**‐**7** and **NPG**. Both document a decrease of the cyanine absorption around 800 nm. A new band between 550 and 600 nm appears in the spectrum, indicating the formation of colored reaction products. This can be, as previously reported, photoproducts formed in the photolysis including the structure shown during the oxidation of **Sens**.^[^ ^21^, ^22^, ^45^ ^]^ Surprisingly, **Sens2** bleaches at 800 nm while no new photoproducts were detected in the visible range between 500 and 600 nm. This stands in contrast to studies with iodonium salts operating as coinitiator where a strong colorization was reported in this wavelength region.^[^ ^60^ ^]^ Thus, the reaction mechanism occurs differently in the system comprising **HABI**/**NPG**. Exposure without **HABI** resulted in similar spectral profiles.

**Figure 5:**
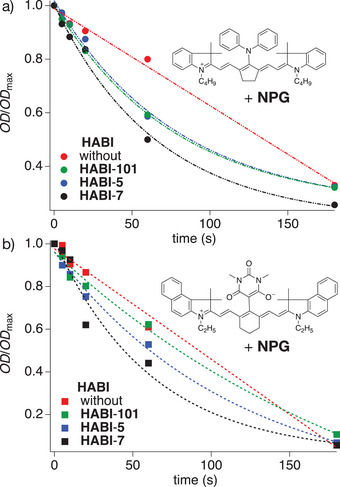
Bleaching kinetics of a) **Sens1** and b) **Sens2** (1.0 × 10^−5^ M) without or with **HABI‐101**, **HABI‐5,** and **HABI‐7** (2.0 × 10^−5^ M) in the presence of the **NPG** (4.0 × 10^−5^ M) using dichloromethane as solvent. Laser emitting 808 nm with an exposure density of 714 mW·cm^−2^. Experiments were pursued under nitrogen.

Figure 5 illustrates normalized changes in the **OD** as a function of exposure time. **Sens1** or **Sens2** resulted with **NPG** in a linear absorption decrease showing linear slopes of −0.0036 and −0.0052 s^−1^, respectively. The oxidation of **NPG** by Sens* can explain this loss of absorption thermodynamically supported by negative Δ*G*_et_, see Table 1. A reductive mechanism responsibly tunes the bleaching. Here, *E*_red_ of **Sens** tunes the thermodynamic scenario according to Equation 1. *E*_red_ of **Sens2** shows slightly smaller values than **Sens1**, confirming the reduction of **Sens1**.

(3) $$\mathrm{Sen}s^{*}+\mathrm{NPG}\to \mathrm{Sen}s^{-\cdot }+\mathrm{NP}G^{+\cdot }$$

(4) $$\mathrm{NP}G^{+\cdot }\to \mathrm{PhNHC}H_{2}\cdot +CO_{2}$$

The *R*_p_^max^ values obtained were low indicating less efficient initiation efficiency, Figure 3b. The scenario changed after adding **HABI**. A certain dependence existed depending on the structure. Adding of **HABI**‐**101**, **HABI**‐**5**, and **HABI**‐**7** resulted in the scenario shown in Figures 3a,b for **Sens1** and **Sens2**, respectively. The data were fit by an exponential function, indicating a more complex reaction mechanism, which exhibited faster bleaching of **Sens** in both graphs. Formation of products absorbing at the exposure wavelength should be of minor importance. An earlier consumption of at least one coinitiator component may cause such results.

The oxidative bleaching of **Sens** with **HABI** can explain this scenario. This reaction, shown in Equation 5, possesses a slight negative Δ*G*_et_, Table 1. **HABI** ^−**·**^ decomposes fast to **L·** and **L** ^−^, Equation 6. The long lifetime of **L·**, depending on the solvent between several microseconds^[^ ^66^ ^]^ and minutes,^[^ ^42^, ^67^, ^68^ ^]^ compared to the excited state of **Sens** (between subnanoseconds^[^ ^69^ ^]^ and nanoseconds^[^ ^24^ ^]^) favors the reaction with an additional component. This can be an electron donor, for which **NPG** represents one candidate. Its oxidation potential of 0.69 V^[^ ^46^ ^]^ enables the reaction with **L·** following an activated electron transfer according to Equation 7 because Δ*G*_et_ appears positive, Table 1. It is also clear that **L** ^−^ and **H** ^+^ fast form **LH**, Equation 8. The latter can additionally react in Equation 9. This process possesses negative Δ*G*_et_ values for all derivatives shown in Table 1, where **LH‐5** gives the most negative data depending on the structure of **Sens**. Here, **Sens1** and **Sens3** resulted in this reaction additionally contributes to the formation of **L·**, which can enter Equation 7 again. Here, a chain reaction starts needing no light resulting in a faster consumption of **HABI** and **NPG**. It explains the curved behavior of all data obtained for systems comprising **HABI** in Figure 5b. In this cycle, Equation 7 starts the cycle followed by Equation 4, forming the initiating radical PhNH‐CH_2_ **·**. **H^+^** enters Equation 8 to protonate **L** ^−^, while the **LH** formed reacts with Sens* resulting in the formation of **L·** in Equation 9. Nevertheless, H‐abstraction of **L·** cannot be completely excluded, but the products detected support more a mechanism based on **PET**. Figure S34 illustrates more absorption spectra.

(5) $$\mathrm{Sen}s^{*}+\mathrm{HABI}\to \mathrm{Sen}s^{+\cdot }+\mathrm{HAB}I^{-\cdot }$$

(6) $$\mathrm{HAB}I^{-\cdot }\to L^{-}+L\cdot$$

(7) $$L\cdot +\mathrm{NPG}\to L^{-}+\mathrm{NP}G^{+\cdot }$$

(8) $$L^{-}+H^{+}⇄L-H$$

(9) $$\mathrm{Sen}s^{*}+\mathrm{LH}\to \mathrm{Sen}s^{-\cdot }+L\cdot +H^{+}$$

**Figure 6:**
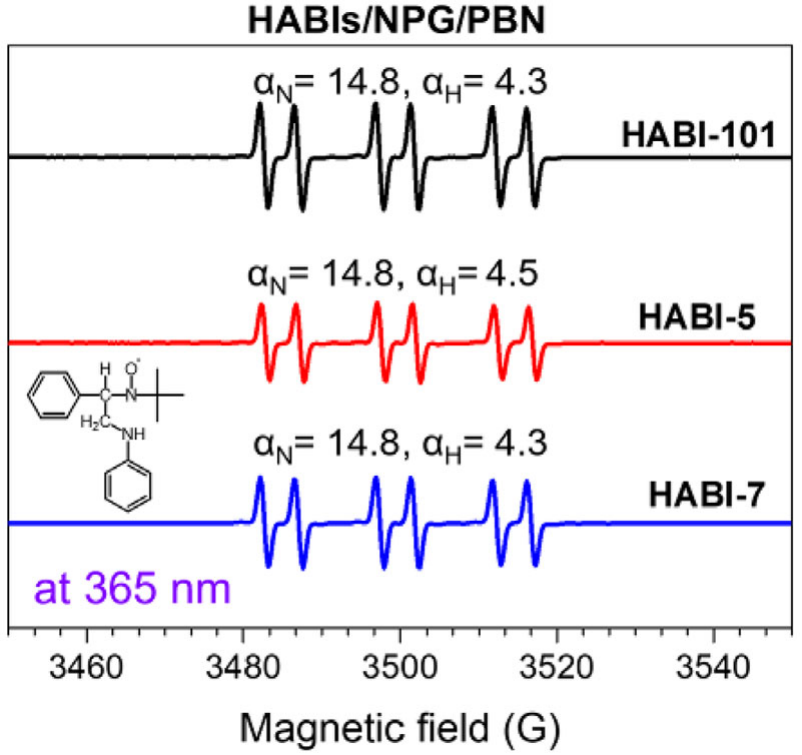
ESR and hyperfine splitting constants generated by dichloromethane solution of **HABI**s/**NPG**/**PBN** systems under irradiation at 365 nm LED source with an exposure density of 50 mW·cm^−2^, (concentration = 2.0 × 10^−4^ M for **HABI**s, 4 × 10^−4^ M for **NPG,** and 4.0 × 10^−4^ M for **PBN**).

**Scheme 4:**
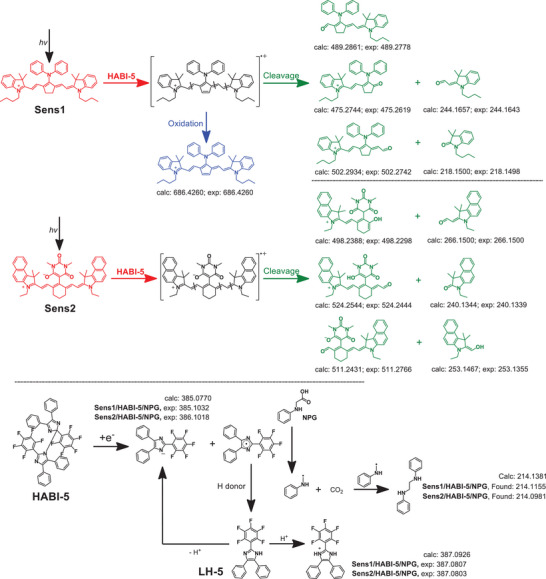
LC‐MS testing results of the **Sens1**/**HABI‐5**/**NPG** and **Sens2**/**HABI‐5**/**NPG** systems in acetonitrile under 808 nm laser source with an exposure intensity of 714 mW·cm^−2^, (concentration at the begin = 0.15 g L^−1^ for **Sens**, 0.3 g L^−1^ for **HABI‐5,** and 0.3 g L^−1^ for **NPG**, exposed solution was appropriately diluted to obtain acceptable separation and signals).

ESR spin trap studies detected the radical PhNH‐CH_2_ **·** using **PBN** as a scavenger, Figure 6. The pattern obtained agrees with that obtained at 365 nm exposure considering the hyperfine coupling constants for N and H.^[^ ^70^, ^71^ ^]^ This approves the formation of this short‐living carbon‐centered radical in the system **Sens**/**HABI**s/**NPG**. It also confirms the mechanistic proposal in Scheme 4. Three different **HABI**s resulted in a similar pattern of the trapped radical. Nevertheless, hydrogen abstraction of **L·** on **NPG** can also competitively proceed resulting in $\mathrm{PhNH}\dot{C}\mathrm{HCOOH}$ but no release of CO_2_. This seems to occur at 365 nm exposure, see Figure 6, upon exposure of **HABI**/**NPG** without **Sens**. However, the results here show that **PET** in the triple system **Sens**/**HABI**s/**NPG** responsibly tunes reactivity. ESR spectra obtained after 808 nm exposure in a solution comprising **Sens** and **HABI**s exhibit a single signal assigned to **L·**. This agrees with that of **L·** disclosed for direct exposure;^[^ ^54^ ^]^ see Figures S35 and S36.

**Figure 7:**
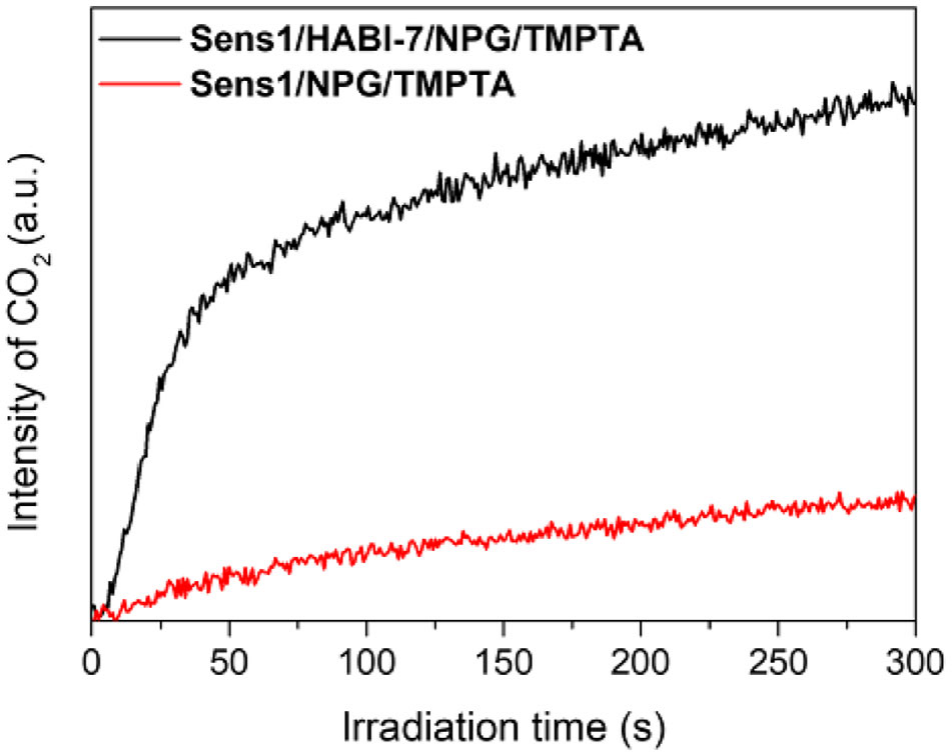
Progression of the IR peak signal intensity of CO_2_ during the photopolymerization process by testing the **Sens1**/**NPG**/**TMPTA** (0.2 mol% for **Sens1** and 2 mol% for **NPG** to **TMPTA**) and **Sens1**/**HABI‐7**/**NPG**/**TMPTA** (0.2 mol% for **Sens1**, 1 mol% for **HABI‐7,** and 2 mol% for **NPG** to **TMPTA**) systems upon irradiation at 808 nm laser (714 mW cm^−2^).

FTIR spectra indicated the formation of CO_2,_ as shown by the band appearing at 2350 cm^−1^. The results significantly demonstrate more CO_2_ formation in the presence of **HABI** compared to a system comprising only **Sens** and **NPG**, Figure 7. Thus, adding **HABI** increases the efficiency of CO_2_ release explaining the higher reactivity observed in Figure 3b. It additionally evidences the better efficiency of radical polymerization with **HABI** indicating a dominating oxidative **PET** between Sens* and **HABI**. Figure S37 provides IR data of different systems.

**Figure 8:**
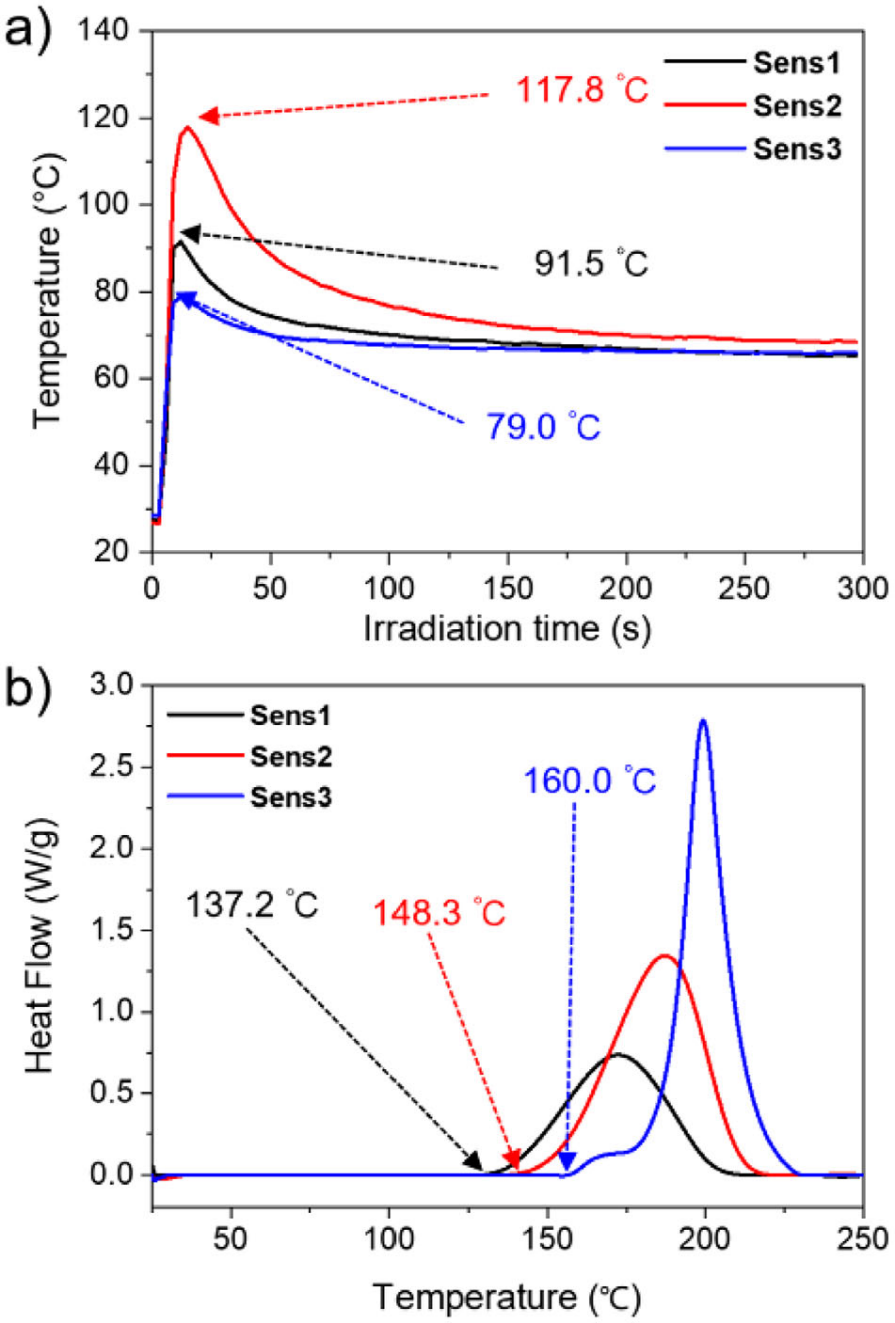
a) The temperature generated by **Sens** (0.2 mol% to **TPGDA**) in the samples (thickness: about 30 µm) comprising **HABI‐5** (1 mol% to **TPGDA**), **NPG** (2 mol% to **TPGDA**), and **TPGDA**. The samples were irradiated with the laser device at 808 nm having an intensity of 714 mW cm^−2^. b) DSC curves of **Sens**/**HABI**‐**5**/**NPG**/**TPGDA** (same ratio as above) systems upon heating using a rate of 10 K min^−1^.

The heat generated by the sensitizer through internal conversion (**IC**) between the lowest vibrational level of the S_1_ and a higher vibrational level of the S_0_ can overcome internal activation barriers of **PET**.^[^ ^21^, ^22^, ^24^, ^25^, ^45^ ^]^ The primary source of the observed thermal energy is the collision of matrix molecules with the very hot ground state of **Sens** conversing the excess energy to heat in the first collision events. The thus formed extremely very hot matrix molecule continues to collide with neighbored matrix molecules resulting in transfer of excess energy to them leading to a distribution of heat in the matrix while itself cooling down. This process, named as vibrational cooling, remains to be fully elucidated. The notion that external heat can contribute efficiently to the process is a misbelief, as previously demonstrated.^[^ ^22^ ^]^ Thus, the temperature depicted in Figure 8a exhibits the average profile obtained during exposure at the time *t*, but it does not relate to the temperature after the first collision. To our knowledge, such data is unavailable and cannot be determined with any standard picosecond setup, a time scale at which vibrations occur. However, there has been existing insufficient and sometimes confusing interpretation due to lack of reliable literature data. One of the best discussions regarding the contribution of vibrations to heat formation was done much earlier.^[^ ^72^ ^]^ Moreover, intramolecular vibrational energy distribution (**IVR**) was sometimes believed as the source to generate heat. Of course, this also proceeds but does not relate to the main route to generate heat in such a photothermal procedure. The extremely hot **Sens** would thermally decompose if **IVR** primarily loses excessive vibrational energy. Supporting Information provide additional data in Figure S38 and Table S4.

Data shown in Figure 8b illustrate quite thermally stable photopolymer compositions. The onset of thermal radical polymerization appears significantly higher compared to those comprising iodonium salts.^[^ ^59^ ^]^ Particularly **Sens3** exhibits the most stable composition approaching that of the neat monomer.^[^ ^59^ ^]^ This can be seen as an additional benefit if these systems will receive more attraction in the future. Thus, the heat formed by internal conversion does not contribute to thermal decomposition of the system with coinitiator while those with iodonium salt did.^[^ ^59^ ^]^ This heat is needed to overcome the internal activation barrier of **PET**.^[^ ^21^, ^45^ ^]^ Supporting Information provide additional data in Figure S39 and Table S5.

Scheme 4 shows the reactions proceeding in the system comprising **Sens**, **HABI**‐**5**, and **NPG**. LC‐MS measurements give a rough pattern regarding the mass of the products formed following an oxidative mechanism, where Sens* and **HABI** react in the first step resulting in formation of **Sens** ^+**·**^ and **HABI**‐**5** ^−**·**^, Equation 5 (Figures S40–S57). In the case of **Sens1**, two consecutive reactions of **Sens** ^+**·**^ compete. This relates to forming an oxidation product comprising one additional double bond. It exhibits, as shown in Figure 4a, a hypsochromic shift. Such a structural pattern no longer assigns to a cyanine and follows other rules between color and structure.^[^ ^21^, ^22^, ^24^ ^]^ The other part shows the decomposition of **Sens** ^+**·**^. **Sens2** only follows the decomposition of **Sens** ^+**·**^. Previous investigations support this proposal^[^ ^21^, ^22^, ^24^ ^]^ following a mechanism based on an oxidative **PET** protocol for **Sens2** ^[^ ^60^ ^]^ and **Sens1**.^[^ ^21^, ^22^, ^24^ ^]^ LC‐MS additionally detected a product assigned to the recombination between two PhNH‐CH_2_ **·** radicals confirming again its formation in a **PET** disclosed in Equation 4. It additionally agrees with the discussion *vide supra*. The negative mode used to operate the MS also facilitated the detection of the lophine anion formed after **PET** between Sens* and **HABI**‐**5**. However, mass spectrometry of pure **LH‐5** resulted in a signal at *m/z* = 385.0987 when operated in negative mode, consistent with data obtained from the exposed solution. This data assign to deprotonated **LH‐5**. The run in positive mode showed a peak at *m/z* = 387.0876 corresponding to the protonated structure of **LH‐5** formed by H‐transfer from the respective H‐donor.

### Dry Film Photoresists

**DFR**s represent important consumables in producing printed circuit boards (**PCB**s). They have been widely used for flexible and stretchable electronics or emerging biomedical applications – just to name a few of them.^[^ ^12^, ^13^ ^]^ NIR **DFR**s, as a new technology, provide promising prospects. Therefore, based on the **Sens**/**HABI**s/**NPG** system, NIR **DFR**s were prepared according to the sketch in Figure S58 and characterized, see Figures S59–S62. Because of the excellent photoinitiating abilities of **HABI**‐**5** and **HABI**‐**7** among the **HABI**s, and the superior thermal stability of **HABI‐5**, they were selected for comparison with the commercial **HABI**‐**101** in NIR **DFR**s.

**Figure 9:**
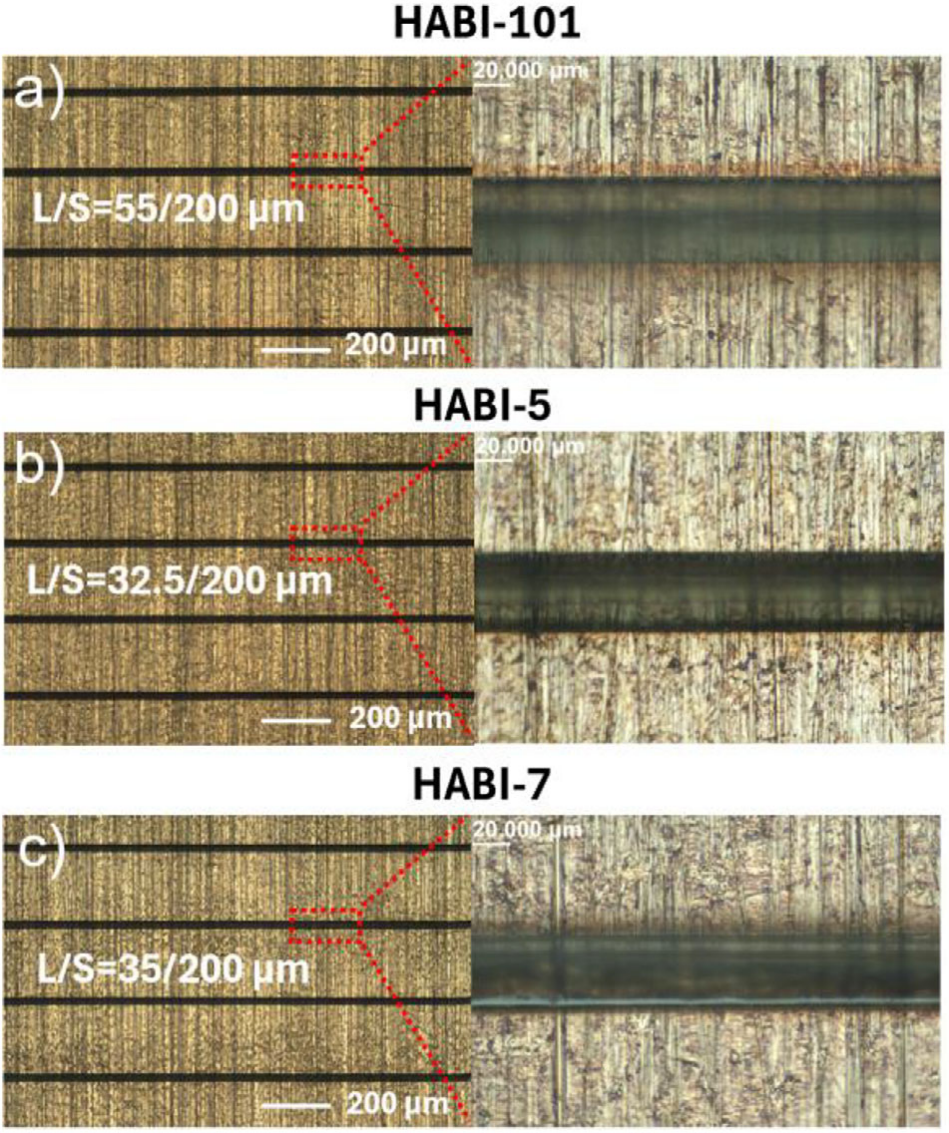
The L/S (L: line width, S: line spacing) of NIR **DFR**s containing **Sens**/**HABI**s/**NPG** systems under intense irradiation at 820 nm with a LED source (1.5 W cm^−2^) for 120 s. Film thickness: 40 µm.

The experimental results (Figure 9) indicate that **HABI**‐**5** and **HABI**‐**7** demonstrate higher resolution overall compared to **HABI**‐**101**, suggesting that they possess greater potential for practical applications.

## Conclusion

This first report about using NIR‐sensitized initiation of radical polymerization using **HABI** and **NPG** as coinitiators brings new directions in the practical field of **DFR**s. This combination brings two components in this field whose photoproducts were not reported to show toxicologic issues compared to some iodonium salts. They additionally do not carry any charge since ions may interfere with insulating properties of the coating. This could raise issues in the applications of electronics.

The design of several **HABI** derivatives also revealed a deeper understanding between its substitution pattern and reactivity. In general, **HABI**s with strong electron‐withdrawing groups performed better than those with electron‐donating substituents (**HABI**‐**2**). **HABI**‐**5**, comprising five fluorine atoms at the phenyl ring, showed the best reactivity. Interestingly, it does not fit into the **PFAS** definition. Future work may address synthesizing more derivatives to receive more effort in this field.

**NPG** performed well as a coinitiator. The release of CO_2_ in the photolysis may change the aerobic medium to an anaerobic one. Nevertheless, there might exist more opportunities. Mercaptotriazol (**MT**) would be one since the first feasibility experiments in the Supporting Information also showed promising results. Here, the initiating radical may not be formed by H‐abstraction, as found earlier. This only works with aliphatic thiols, but this process proceeds significantly slower, as shown in a previous report. However, the surroundings responsibly determine the ratio of thion in the thion/thiol tautomeric equilibrium where the thiol form seems responsible for the reactivity with the lophyl radical. Thus, a change in the matrix monomer could have a drastic influence on reactivity. From this point of view, **NPG** seems to be a promising alternative with more robustness by changing the surroundings.

In principle, heptamethine cyanines are suitable for dry film photoresists when a **HABI** derivative with a strong electron acceptor function combined with **NPG** as donor operated as coinitiators. This has the advantage that less toxic photolysis products are formed than when iodonium salts are used. The fact that these systems are based on a **PET** with an internal activation barrier also gives them a certain stability under moderate room light conditions since radical photopolymerization can only be initiated with intense NIR light sources. This is interesting from a practical viewpoint and has the potential to be an attractive candidate over UV **DFR**s, which can be exposed to room light and, therefore, require processing under a special safety light. To conclude, NIR‐**DFR**s could be expected to receive more attention as the next generation of such **DFR**s instead of UV‐**DFR**s. Such a system could find practical use in flexible and transparent electronic devices.

The system introduced here exhibits much higher thermal stability than those comprising an iodonium salt, which is known to exhibit less thermal stability. Future work should focus on understanding how cyanine and **HABI** affect reactivity by including more samples. Such systems possess more potential by synthesizing more **HABI** structures with alternative electron withdrawing patterns. In addition, modifying **NPG** to result in a branched pattern would positively impact network density, an important parameter in making resists.

## Supporting Information

The Wiley Online Library provides supporting information on material synthesis, photolysis, thermal stability, thermal imaging, and **DFR**s preparation. This also shows more data regarding absorption, photopolymerization experiments, and mass spectrometry. Quantum chemical calculations complement this part.

## Conflict of Interests

The authors declare no conflict of interest.

## Supporting information



Supporting Information

## Data Availability

The data that support the findings of this study are available from the corresponding author upon reasonable request.
