# Supplementary material for: NIR‐Sensitized Activated Formation of Lophyl Radicals by Heptamethine Cyanines Enables Dry Film Photoresists
Source: Angew Chem Int Ed Engl. 2025 Apr 3;64(19):e202422700. doi: 10.1002/anie.202422700 (PMC12051825; doi:10.1002/anie.202422700)
Supplement: Supplementary file 1 — Supporting Information [file ANIE-64-e202422700-s001.docx]

**Table of Contents**

[Materials and Procedures 2](#_Toc187178300)

[Materials 2](#_Toc187178301)

[Characterization Methods for the Materials 2](#_Toc187178302)

[Irradiation Sources 2](#_Toc187178303)

[Electron Spin Resonance (ESR) Experiments 3](#_Toc187178304)

[Steady-State Photolysis 3](#_Toc187178305)

[Computational Procedures 3](#_Toc187178306)

[Photopolymerization Experiments 3](#_Toc187178307)

[Redox Potentials 4](#_Toc187178308)

[Preparation and Process of Dry Film Photoresist (DFRs) 4](#_Toc187178309)

[Thermal Stability Tests 4](#_Toc187178310)

[Thermal Imaging 5](#_Toc187178311)

[Cytotoxicity Tests 5](#_Toc187178312)

[Synthesis of HABIs 6](#_Toc187178313)

[SUPPLEMENTARY DATA 9](#_Toc187178314)

[Cytotoxic Data 9](#_Toc187178315)

[Cyclic Voltammetry Data 10](#_Toc187178316)

[Optical Properties of Sens and HABIs 18](#_Toc187178317)

[Photopolymerization Experiments 19](#_Toc187178318)

[Quantum Chemical Calculations 24](#_Toc187178319)

[Photochemical Properties of Sens/HABI/NPG Systems 27](#_Toc187178320)

[Effect of Heat on Photopolymerization Reaction 30](#_Toc187178321)

[LC-MS Data of Sens/HABI-5/NPG System 34](#_Toc187178322)

[Preparation and Characterization of Dry Film Photoresist (DFR) 40](#_Toc187178323)

[Nuclear Magnetic Resonance Data 42](#_Toc187178324)

[Mass Spectrometry Data 46](#_Toc187178325)

[References 53](#_Toc187178326)

# Materials and Procedures

## Materials

The 1-butyl-2-((*E*)-2-((*E*)-3-(2-((*E*)-1-butyl-3,3-dimethylindolin-2-ylidene)ethylidene)-2-(diphenylamino)cyclo-pent-1-en-1-yl)vinyl)-3,3-dimethyl-3*H*-indol-1-ium tetrafluoroborate (**Sens1**), 5-((*E*)-6-((*E*)-2-(3-ethyl-1,1-dimethyl-1,3-dihydro-2*H*-benzo[e]indol-2-ylidene)ethylidene)-2-((*E*)-2-(3-ethyl-1,1-dimethyl-1*H*-benzo[e]indol-3-ium-2-yl)vinyl)cyclohex-1-en-1-yl)-1,3-dimethyl-2,6-dioxo-1,2,3,6-tetrahydropyrimidin-4-olate (**Sens2**), and 1-butyl-2-((*E*)-2-((*E*)-3-(2-((*E*)-1-butyl-3,3-dimethylindolin-2-ylidene)ethylidene)-2-(phenylthio)cyclohex-1-en-1-yl)vinyl)-3,3-dimethyl-3*H*-indol-1-ium tetraphenylborate (**Sens3**) were obtained from FEW Chemicals GmbH. Tri-(propylene glycol) diacrylate (**TPGDA**) and tri-methylolpropane triacrylate (**TMPTA**) were obtained from Sanmu Group. Ethyl bisphenol A dimethacrylate (**SR480NS**, m+n=10) was acquired from Guangzhou Sartomer. 2-Fluorobenzaldehyde, 4-cyanobenzaldehyde, 4-(methylthio)benzaldehyde, pentafluorobenzaldehyde, 4-(trifluoromethyl)benzaldehyde, benzil, 4,4'-difluorobenzil, 4,4'-dimethoxybenzil, ammonium acetate, *N*-phenylglycine (**NPG**), mercapto triazole (**MT**) potassium ferricyanide, tetrabutylammonium hexafluorophosphate and ferrocene were provided by Beijing Innochem Chemical. Spin trapping reagents *N*-*tert*-butyl-2-phenylnitrone (**PBN**) was provided by Shanghai Bide Pharmatech. 2,2'-Bis(2-chlorophenyl)-4,4',5,5'-tetraphenyl-1,2'-biimidazole (**HABI-101**) was supplied by Changzhou Powerful Photoelectric Materials. Bis(4-*t*-butyl phenyl)iodonium hexafluorophosphate was obtained from Hubei Gurun Technology. Anhydrous acetonitrile, dichloromethane (CH_2_Cl_2_), acetic acid, methanol, ethyl acetate, petroleum ether, sodium hydroxide (NaOH) and anhydrous sodium sulfate (NaSO_4_) were supplied by Beijing Chemical Work. Deuterated dimethyl sulfoxide (*d_6_*-DMSO) was provided by Sinopharm Chemical.

## Characterization Methods for the Materials

The ^1^H nuclear magnetic resonance (NMR, 400 MHz) spectra of **HABI**s were recorded using a Bruker AV400 unity spectrometer. The high-resolution mass spectra (HRMS) were obtained by Agilent 6540 QToF mass detector equipped with an electrospray ionization source. The LC-MS analysis was conducted utilizing an Agilent 1260 high-performance liquid chromatography system coupled with a Bruker micrOTOF-QⅡquadrupole time-of-flight tandem mass spectrometer. The UV-vis-NIR absorption properties of **HABI**s and **Sens**, steady-state photolysis behaviors of **Sens/HABI**s**/NPG** and **Sens/NPG** systems, were recorded by Shimadzu UV-3600 UV−vis−NIR spectrophotometer.

## Irradiation Sources

The NIR laser source MDL-H-808-5W, manufactured by Changchun New Industries Optoelectronics Tech, was utilized as the irradiation device with an emission wavelength of 808 nm. Additionally, the 820 nm LED light source UVEC-60X20 can be obtained from Shenzhen Lamplic. The ShangHai Futansi UVSP-81T LED bulb, which emitted at concentrated wavelengths of 365 nm, was also employed as an irradiation device.

## Electron Spin Resonance (ESR) Experiments

ESR tests on **HABI**s**/NPG** systems and **Sens/HABI**s**/NPG** systems were conducted using a JEOL JES-FA200 spectrometer (X-band). The tests were performed at 9.06 GHz and 100 kHz field modulation, with a microwave power of 0.998 mW. The free radicals were generated by **Sens/HABI**s**/NPG** systems through irradiation at 808 nm laser (714 mW cm^−2^) at room temperature, and then trapped by **PBN**. The mole ratio of **Sens/HABI**s**/NPG/PBN** systems was 0.1:0.5:1:1. These systems were dissolved in dichloromethane, with a concentration of **Sens** at 1 × 10^−4^ mol L^−1^. Subsequently, the free radicals generated by **HABI**s**/NPG** systems were trapped by **PBN** also at room temperature through irradiation at 365 nm LED (50 mW cm^−2^). The mole ratio of **HABI**s**/NPG/PBN** systems was 0.5:1:1. These systems were dissolved in dichloromethane, with a concentration of **HABI**s at 2 × 10^−4^ mol L^−1^.

In addition, the imidazole radical generated by the **Sens/HABI**s systems under 808 nm laser irradiation (714 mW cm^−2^) was also detected without **PBN** at room temperature. The mole ratio of **Sens/HABI**s systems was 1:5. These systems were dissolved in dichloromethane, with a concentration of **Sens** at 1 × 10^−3^ mol L^−1^.

## Steady-State Photolysis

The steady-state photolysis tests were conducted by preparing anhydrous dichloromethane solutions of **Sens/HABI**s**/NPG** (1×10^-5^ mol L^-1^ for **Sens**, 2×10^-5^ mol L^-1^ for **HABI**s and 4×10^-5^ mol L^-1^ for **NPG**) and **Sens/NPG** (1×10^-5^ mol L^-1^ for **Sens**, and 4×10^-5^ mol L^-1^ for **NPG**). The irradiation intensity was 714 mW cm^−2^, corresponding to an 808 nm laser.

## Computational Procedures

The theoretical calculation of **Sens** and **HABI**s were executed using Gaussian 09W software, based on density function theory (DFT). The frontier molecular orbits and energies of **Sens** and **HABI**s were analyzed by optimizing molecular geometries at B3LYP/6-31G* level. Molecular transition methods of **Sens** were calculated at the TD/B3LYP/6-31G(d) levels. The bond dissociation energies (BDE) at the cleavage sites of **HABI**s can be calculated at the B3LYP/6-31G(d) and UB3LYP/6-31G(d) levels. The atomic charge values and spin density of the **L•** generated by **HABI**s were analysed by optimizing the molecular geometries and frequency calculations at the UB3LYP/6-31G(d) levels, then the atomic charge values were calculated via the atomic dipole moment-corrected Hirshfeld (ADCH) method with Multiwfn software.^[1]^ Multiwfn and VMD software were used for drawing and visual analysis.

## Photopolymerization Experiments

The photopolymerization kinetics experiments were conducted on photosensitive formulas consisting of **Sens/HABI**s**/donor**s (**NPG** or **MT**) systems or **Sens/donor**s systems and polymerizable **monomer**s of **TPGDA** or **TMPTA** using a Nicolet 5700 FT-IR spectroscope. The mole ratios were set as 0.002:0.01:0.02:1 for **Sens/HABI**s**/donors/monomer**s systems, and 0.002:0.02:1 for **Sens/NPG/monomer**s systems. The photosensitive formulas were injected into a laminate with a thickness of approximately 30 *μ*m between two KBr plates, and irradiated using laser at 808 nm (714 mW cm^−2^). In addition, **HABI**s**/NPG/TPGDA** systems with a 0.01:0.02:1 molar ratio were also tested under 365 nm source irradiation (50 mW cm^−2^). The conversions of the C=C bond for **TPGDA** or **TMPTA** were calculated considering the decrease of the double bond area of 1660-1600 cm^-1^ or 820-773 cm^-1^ with respect to the peak area of 1720 cm^−1^,^[2-4]^ Eq. SI1.

$x=\left[ 1-\frac{{A(C=C)}_{t}/{A(C=C)}_{t=0}}{{A(C=O)}_{t=0}/{A(C=O)}_{t=0}} \right]\times100\%$ (SI1)

Furthermore, by integrating the peak (around 2350 cm^−1^) of the carbon dioxide generated by the **Sens1/NPG/monomer**s and **Sens1/HABI-7/NPG/monomer**s systems under irradiation at 808nm source, the signal intensity variations of carbon dioxide were obtained. The testing method is the same as mentioned above.

## Redox Potentials

The redox potentials of **HABI**s and **LH**s were investigated using a Shanghai Chenhua CHI660C electrochemical workstation. Tetrabutylammonium hexafluorophosphate in anhydrous acetonitrile (0.1 mol L^-1^) was employed as a supporting electrolyte, ferrocene was used as an external standard. The anhydrous acetonitrile solutions of **HABI**s and **LH**s (1×10^-3^ mol L^-1^) were tested by using platinum disk as a working electrode and Ag/AgCl as reference electrode with a scanning rate of 0.1 V s^-1^.

## Preparation and Process of Dry Film Photoresist (DFRs)

Three **DFR**s were prepared by mixing and stirring a specific ratio of **Sens1** and **Sens3**, **HABI**s (**HABI-101, HABI-5,** or **HABI-7** respectively), **NPG**, **SR480NS**, **resin S80-1** (provided by Nanjing University of Science and Technology), methanol and acetone. The **DFR**s were applied onto a polyester film using a scraper, ensuring that the thickness of **DFR**s is approximately 40μm, and then placed in a vacuum oven at a temperature of 90℃ for 3 mins. Afterwards, the **DFR**s were attached onto a copper plate, forming a sandwich-like structure with the copper plate at the bottom, the **DFR**s in the middle, and the polyester film on top, creating a condition that isolates the oxygen. Exposure was performed utilizing an 820 nm LED light source (1.5 W cm^−2^), followed by a quiescent period of 30 mins. Subsequently, a 1% sodium carbonate solution at a temperature of 30 ℃ was used for development, followed by rinsing with water to accomplish the patterning of **DFR**s. The resulting patterns were imaged using an optical microscope (12XB-PC, Shanghai optical instrument factory) at a magnification of 50x.

## Thermal Stability Tests

The thermal stability of photosensitive formulas (same as photopolymerization experiments section) and **DFR**s were evaluated using a differential scanning calorimetry (DSC) instrument (METTLER TOLEDO Company DSC1). Approximately 4 mg – 6 mg of the photosensitive formulas or **DFR**s were heated from 25 ℃ to 250 ℃ at a rate of 10 ℃ min^−1^ under N_2_ atmosphere. The initiation of polymerization was determined by identifying the onset of the first exothermic peak.

## Thermal Imaging

An infrared thermal imaging camera Testo 890 was employed to monitor the reaction temperature. The formulas and the light intensity of irradiation sources were kept consistent with the sections of photopolymerization experiments. These formulas were sandwiched between two transparent glass slides (approximately 30 μm thick) at a temperature of 20 ± 2 ℃. The temperature variations during 300 seconds of irradiation of the sandwich were recorded.

## Cytotoxicity Tests

L929 cells were cultured in a medium with 10% horse serum and incubated overnight at 37°C in a humidified atmosphere with 5% CO_2_. Once the cells reached the logarithmic growth phase, they were digested with trypsin, counted, and adjusted to 5×10⁴ cells mL^-1^. 96-well plates were seeded with 5000 cells/well and incubated. Solutions of different **HABI**s at concentrations of 0.56, 1.12, 2.23, 4.43, and 8.92 mmol L^-1^, along with iodonium salt as references, were added to the cells. After 24 hours, the medium was removed, and CCK-8 reagent was used to assess cell viability. CCK-8 was diluted 1:10 with medium, and 100 μL was added per well, followed by incubation for 2 hours. Optical density (OD) at 450 nm was measured using a microplate reader. Cell viability was calculated by comparing the results those from the blank (medium + CCK-8 without cells) and control (cells + medium + CCK-8 without **HABI**s or iodonium salt) samples.^[5]^ The cell viability can be calculated by Eq. SI2:

$\mathrm{Viability}=\frac{{OD}_{\mathrm{sample}}-{OD}_{blank sample}}{{OD}_{control sample}-{OD}_{blank sample}}$ (SI2)

## Synthesis of HABIs

The synthesis routes of **HABI**s are illustrated in **Scheme SI1**^[6]^, and the specific details are described below.

**Scheme SI1:** The synthesis routes of **HABI**s

**2,2'****-Bis(2-fluorophenyl)-4,4',5,5'-tetraphenyl-2'H-1,2'-biimidazole (HABI-1)**

2-Fluorobenzaldehyde (1.24 g, 10 mmol), benzil (1.68 g, 8 mmol) and ammonium acetate (3.08 g, 40 mmol) were dissolved in 50 mL of acetic acid and heated to 120 ℃ with stirring for 5 hours. The reaction was conducted under N_2_ atmosphere, and the progress of the reaction was monitored by thin-layer chromatography (TLC). After the completion of the reaction, the mixture was poured into a 200 mL aqueous solution of sodium bisulfite (concentration of 20 g L^-1^) and stirred for 5 mins, and then filtered to obtain the intermediate product **LH-1** (milky white solid, yield of 81%). HRMS (ESI) [M + H]^+^ calcd for C_21_H_15_FN_2_^+^ 315.1253, found 315.1311.

Second step, **LH-1** (1.26 g, 4 mmol) and potassium ferricyanide (3.95 g, 12 mmol) were dissolved in a mixture of deionized water (70 mL) and dichloromethane (30 mL), then stirred at 25 ℃. Subsequently, 30 mL aqueous solution of sodium hydroxide with a concentration of 200 g L^-1^ was gradually added dropwise to the aforementioned mixture (1 s per drop). After the addition, the reaction temperature was increased to 40 °C and kept the temperature for 4 hours, and the progress of the reaction was monitored by thin-layer chromatography (TLC). Then the organic layer was separated and aqueous layer was extracted with ethyl acetate (3 × 20 mL). Subsequently, organic layers were combined, dried by anhydrous sodium sulfate, and removed most of the solvent by vacuum distillation. Slowly add petroleum ether into the above solution until a large amount of the product precipitates, and then filter, dry to obtain the pure **HABI-1** (pale yellow solid, yield of 73 %).^1^H NMR (400 MHz, DMSO-d, ppm) δ 7.58 (m, 3H), 7.41 (t, 5H), 7.26 (m, 12H), 7.09 (m, 5H), 6.95 (ddd, 1H), 6.65 (m, 2H). HRMS (ESI) [M + H]^+^ calcd for C_42_H_28_F_2_N_4_^+^ 627.2316, found 627.3126.

**2,2'-bis(4-(methylthio)phenyl)-4,4',5,5'-tetraphenyl-2'H-1,2'-biimidazole (HABI-2)**

4-(Methylthio)benzaldehyde (1.52 g, 10 mmol), benzil (1.68 g, 8 mmol) and ammonium acetate (3.08 g, 40 mmol) were dissolved in 50 mL of acetic acid and heated to 120 ℃ with stirring for 5 hours. The remaining experimental steps are the same as the synthesis of **LH-1**. Finally, **LH-2** was obtained (yellow solid, yield of 72%). HRMS (ESI) [M + H]^+^ calcd for C_22_H_18_N_2_S^+^ 343.1124, found 343.1256.

Second step, **LH-2** (1.37 g, 4 mmol) and potassium ferricyanide (3.95 g, 12 mmol) were dissolved in a mixture of deionized water (70 mL) and dichloromethane (30 mL), then stirred at 25 ℃. The remaining experimental steps are the same as the synthesis of **HABI-1**. Finally, pure **HABI-2** was obtained (orange-yellow solid, yield of 78 %).^1^H NMR (400 MHz, DMSO-d, ppm) δ 7.53 (t, 2H), 7.39 (m, 6H), 7.19 (m, 16H), 7.03 (d, 2H), 6.88 (d, 2H), 2.46 (s, 3H), 2.37 (s, 3H). HRMS (ESI) [M + H]^+^ calcd for C_44_H_34_N_4_S_2_^+^ 683.2258, found 683.3155.

**4,4'-(4,4',5,5'-tetrakis(4-methoxyphenyl)-2'H-[1,2'-biimidazole]-2,2'-diyl)dibenzonitrile (HABI-3)**

4-Cyanobenzaldehyde (1.31 g, 10 mmol), 4,4'-dimethoxybenzil (2.16 g, 8 mmol) and ammonium acetate (3.08 g, 40 mmol) were dissolved in 50 mL of acetic acid and heated to 120 ℃ with stirring for 5 hours. The remaining experimental steps are the same as the synthesis of **LH-1**. Finally, **LH-3** was obtained (pale yellow solid, yield of 68%). HRMS (ESI) [M + H]^+^ calcd for C_24_H_19_N_3_O_2_^+^ 382.1511, found 382.1543.

Second step, **LH-3** (1.53 g, 4 mmol) and potassium ferricyanide (3.95 g, 12 mmol) were dissolved in a mixture of deionized water (70 mL) and dichloromethane (30 mL), then stirred at 25 ℃. The remaining experimental steps are the same as the synthesis of **HABI-1**. Finally, pure **HABI-3** was obtained (yellow solid, yield of 78 %).^1^H NMR (400 MHz, DMSO-d, ppm) δ 7.61 (m, 4H), 7.46 (d, 2H), 7.22 (m, 8H), 7.06 (d, 2H), 6.92 (d, 4H), 6.73 (d, 2H), 6.68 (d, 2H), 3.78 (s, 6H), 3.67 (s, 3H), 3.62 (s, 3H). HRMS (ESI) [M + H]^+^ calcd for C_48_H_36_N_6_O_4_^+^ 761.2832, found 761.3853.

**4,4'-(4,4',5,5'-tetraphenyl-2'H-[1,2'-biimidazole]-2,2'-diyl)dibenzonitrile (HABI-4)**

4-Cyanobenzaldehyde (1.31 g, 10 mmol), benzil (1.68 g, 8 mmol) and ammonium acetate (3.08 g, 40 mmol) were dissolved in 50 mL of acetic acid and heated to 120 ℃ with stirring for 5 hours. The remaining experimental steps are the same as the synthesis of **LH-1**. Finally, **LH-4** was obtained (pale yellow solid, yield of 74%). HRMS (ESI) [M + H]^+^ calcd for C_22_H_15_N_3_^+^ 322.1300, found 322.1344.

Second step, **LH-4** (1.29 g, 4 mmol) and potassium ferricyanide (3.95 g, 12 mmol) were dissolved in a mixture of deionized water (70 mL) and dichloromethane (30 mL), then stirred at 25 ℃. The remaining experimental steps are the same as the synthesis of **HABI-1**. Finally, pure **HABI-4** was obtained (pale yellow solid, yield of 77%).^1^H NMR (400 MHz, DMSO-d, ppm) δ 7.71 (t, 4H), 7.57 (t, 2H), 7.51 (d, 2H), 7.41 (t, 4H), 7.28 (m, 13H), 7.14 (m, 3H). HRMS (ESI) [M + H]^+^ calcd for C_44_H_28_N_6_^+^ 641.2409, found 641.3244.

**2,2'-bis(perfluorophenyl)-4,4',5,5'-tetraphenyl-2'H-1,2'-biimidazole (HABI-5)**

Pentafluorobenzaldehyde (1.96 g, 10 mmol), benzil (1.68 g, 8 mmol) and ammonium acetate (3.08 g, 40 mmol) were dissolved in 50 mL of acetic acid and heated to 120 ℃ with stirring for 5 hours. The remaining experimental steps are the same as the synthesis of **LH-1**. Finally, **LH-5** was obtained (pale yellow solid, yield of 81%). HRMS (ESI) [M + H]^+^ calcd for C_21_H_11_F_5_N_2_^+^ 387.0876, found 387.0923.

Second step, **LH-5** (1.55 g, 4 mmol) and potassium ferricyanide (3.95 g, 12 mmol) were dissolved in a mixture of deionized water (70 mL) and dichloromethane (30 mL), then stirred at 25 ℃. The remaining experimental steps are the same as the synthesis of **HABI-1**. Finally, pure **HABI-5** was obtained (orange-yellow solid, yield of 71%).^1^H NMR (400 MHz, DMSO-d, ppm) δ 7.66 (t, 2H), 7.51 (t, 4H), 7.46 (m, 6H), 7.39 (m, 3H), 7.32 (d, 2H), 7.19 (m, 3H). HRMS (ESI) [M + H]^+^ calcd for C_42_H_20_F_10_N_4_^+^ 771.1526, found 771.2577.

**4,4'-(4,4',5,5'-tetrakis(4-fluorophenyl)-2'H-[1,2'-biimidazole]-2,2'-diyl)dibenzonitrile (HABI-6)**

4-Cyanobenzaldehyde (1.31 g, 10 mmol), 4,4'-difluorobenzil (1.97 g, 8 mmol) and ammonium acetate (3.08 g, 40 mmol) were dissolved in 50 mL of acetic acid and heated to 120 ℃ with stirring for 5 hours. The remaining experimental steps are the same as the synthesis of **LH-1** Finally, **LH-6** was obtained (pale yellow solid, yield of 66%). HRMS (ESI) [M + H]^+^ calcd for C_22_H_13_F_2_N_3_^+^ 358.1111, found 358.1163.

Second step, **LH-6** (1.43 g, 4 mmol) and potassium ferricyanide (3.95 g, 12 mmol) were dissolved in a mixture of deionized water (70 mL) and dichloromethane (30 mL), then stirred at 25 ℃. The remaining experimental steps are the same as the synthesis of **HABI-1**. Finally, pure **HABI-6** was obtained (pale yellow solid, yield of 63%).^1^H NMR (400 MHz, DMSO-d, ppm) δ 7.67 (s, 4H), 7.55 (d, 2H), 7.39 (ddd, 4H), 7.30 (m, 10H), 7.05 (m, 4H). HRMS (ESI) [M + H]^+^ calcd for C_44_H_24_F_4_N_6_^+^ 713.2032, found 713.2989.

**4,4',5,5'-tetrakis(4-fluorophenyl)-2,2'-bis(4-(trifluoromethyl)phenyl)-2'H-1,2'-biimidazole (HABI-7)**

4-(Trifluoromethyl)benzaldehyde (1.74 g, 10 mmol), 4,4'-difluorobenzil (1.97 g, 8 mmol) and ammonium acetate (3.08 g, 40 mmol) were dissolved in 50 mL of acetic acid and heated to 120 ℃ with stirring for 5 hours. The remaining experimental steps are the same as the synthesis of **LH-1**. Finally, **LH-7** was obtained (pale yellow solid, yield of 78%). HRMS (ESI) [M + H]^+^ calcd for C_22_H_13_F_5_N_2_^+^ 402.1066, found 402.1602.

Second step, **LH-7** (1.60 g, 4 mmol) and potassium ferricyanide (3.95 g, 12 mmol) were dissolved in a mixture of deionized water (70 mL) and dichloromethane (30 mL), then stirred at 25 ℃. The remaining experimental steps are the same as the synthesis of **HABI-1**. Finally, pure **HABI-7** was obtained (pale yellow solid, yield of 76%).^1^H NMR (400 MHz, DMSO-d, ppm) δ 7.67 (d, 2H), 7.55 (d, 2H), 7.37 (m, 8H), 7.28 (m, 8H), 7.02 (td, 4H). HRMS (ESI) [M + H]^+^ calcd for C_44_H_24_F_10_N_4_^+^ 799.1875, found 799.2936.

Figures S63-S69 and Figures S70-S83 provide the necessary NMR spectra and mass spectrometry data, respectively, at the end of this SI.

# SUPPLEMENTARY DATA

## Cytotoxic Data

**Table S1** Cytotoxic effects for different concentrations of **HABIs** and iodonium salt with L929 cells

|  | Cell viability (%) | | | | |
| --- | --- | --- | --- | --- | --- |
| concentration of loading | 0.56 mM | 1.12 mM | 2.23 mM | 4.43 mM | 8.92 mM |
| **HABI-101** | 101.6 | 108.4 | 109.5 | 108.8 | 107.1 |
| **HABI-1** | 110.7 | 110.8 | 107.3 | 97.9 | 96.2 |
| **HABI-2** | 104.4 | 104.1 | 103.8 | 105.1 | 71.7 |
| **HABI-3** | 111.1 | 113.4 | 113.0 | 103.6 | 109.3 |
| **HABI-4** | 99.8 | 94.4 | 94.4 | 100.4 | 78.5 |
| **HABI-5** | 11.5 | 5.8 | 8.1 | 14.3 | 23.5 |
| **HABI-6** | 103.7 | 107.7 | 111.1 | 100.7 | 97.0 |
| **HABI-7** | 101.8 | 98.9 | 97.8 | 104.4 | 78.7 |
| Iodonium salt | 2.7 | 3.2 | 9.3 | 36.4 | 48.0 |

## Cyclic Voltammetry Data


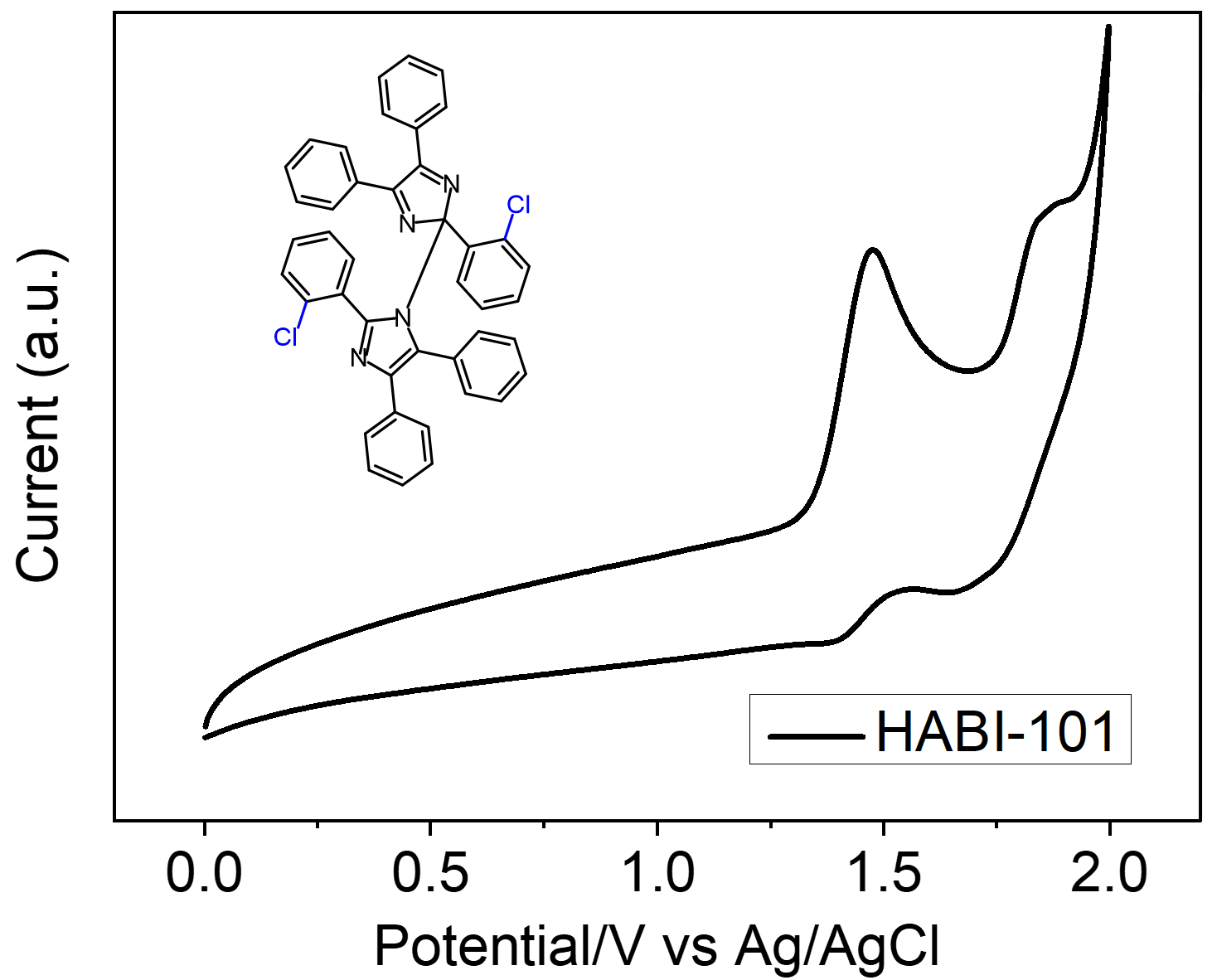


**Figure S1.** The cyclic voltammetry curve of the acetonitrile solution of **HABI-101** (1×10^-3^ mol L^-1^), used to determine the *E*_ox_.


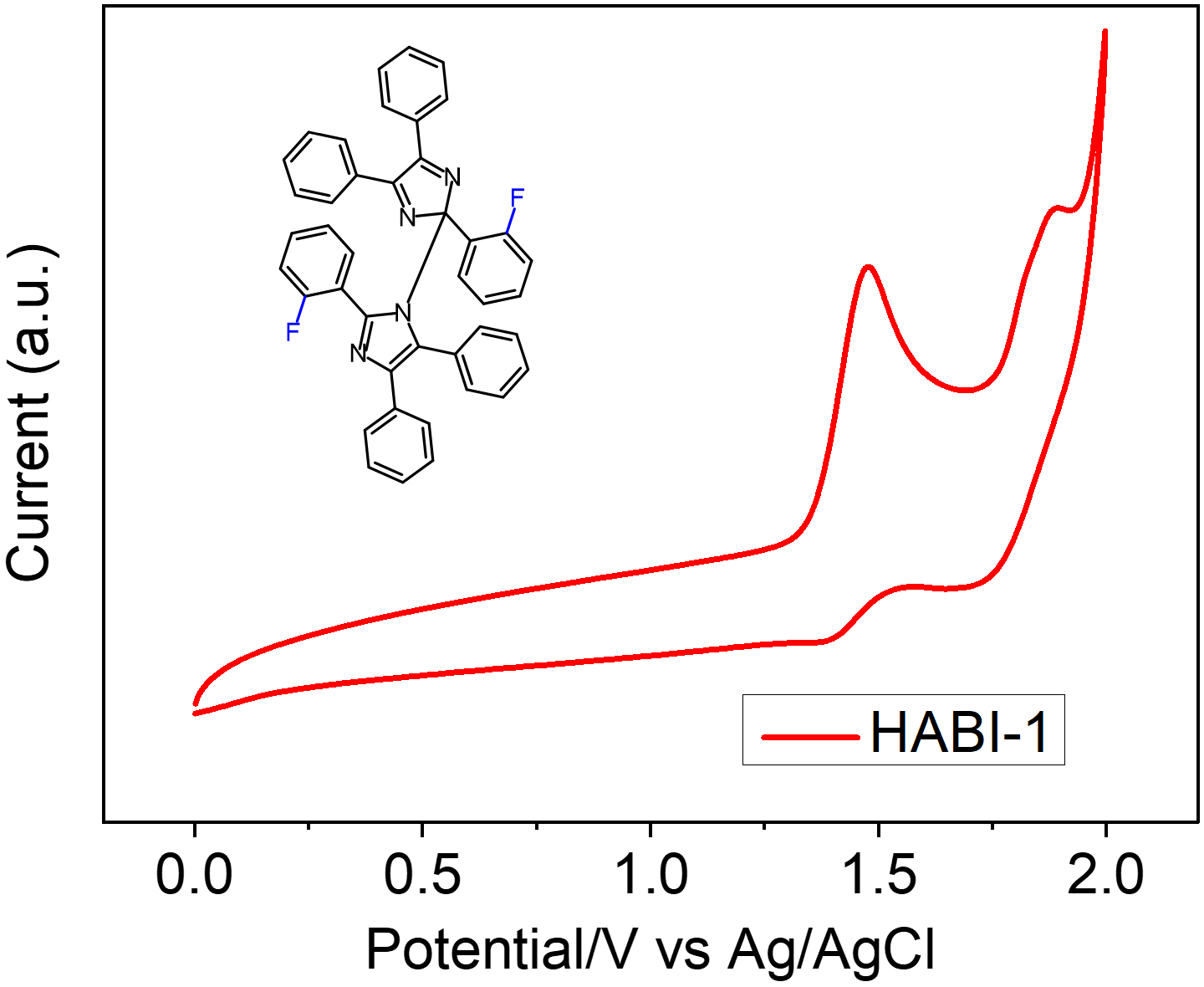


**Figure S2.** The cyclic voltammetry curve of the acetonitrile solution of **HABI-1** (1×10^-3^ mol L^-1^), used to determine the *E*_ox_.


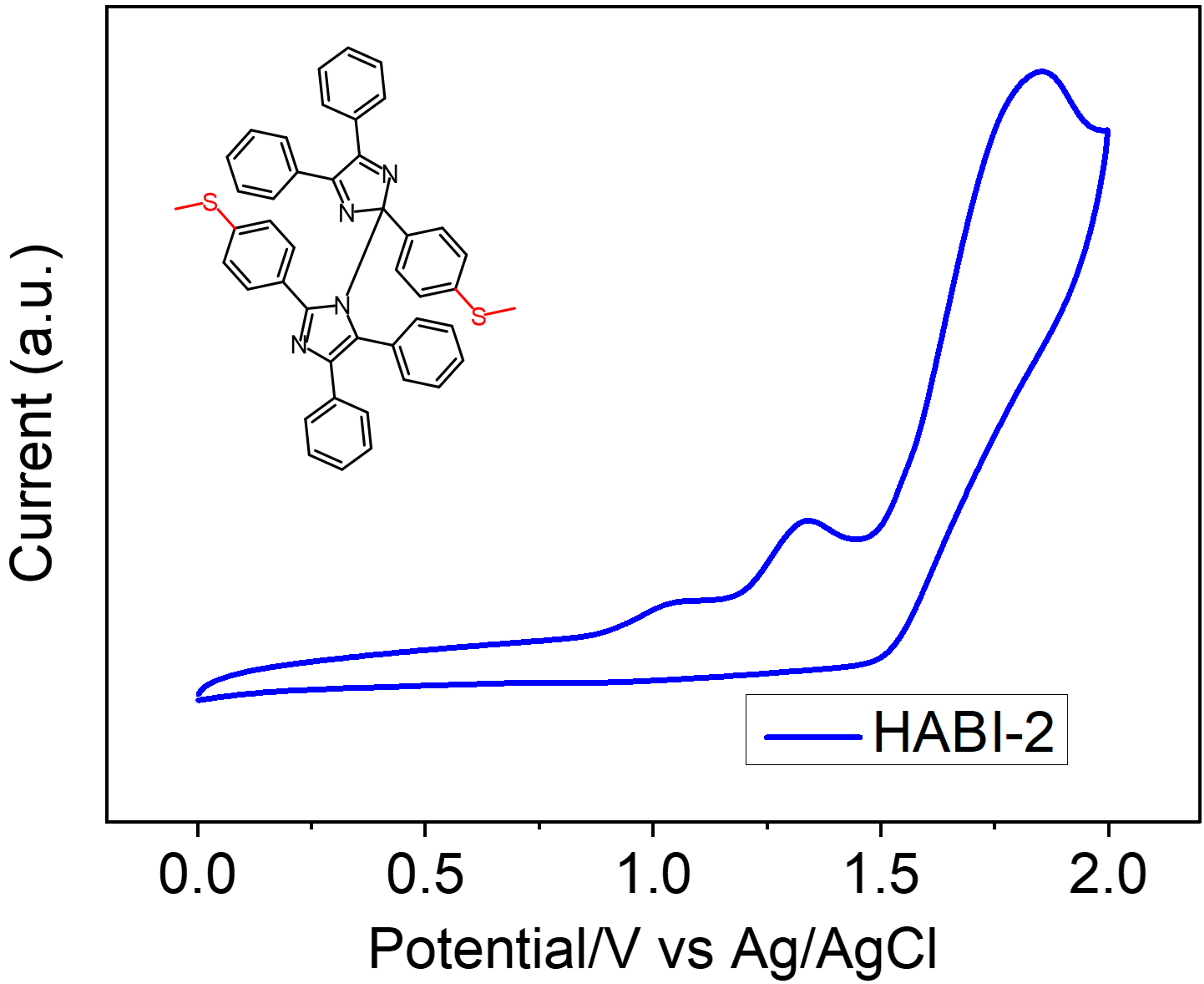


**Figure S3.** The cyclic voltammetry curve of the acetonitrile solution of **HABI-2** (1×10^-3^ mol L^-1^), used to determine the *E*_ox_.


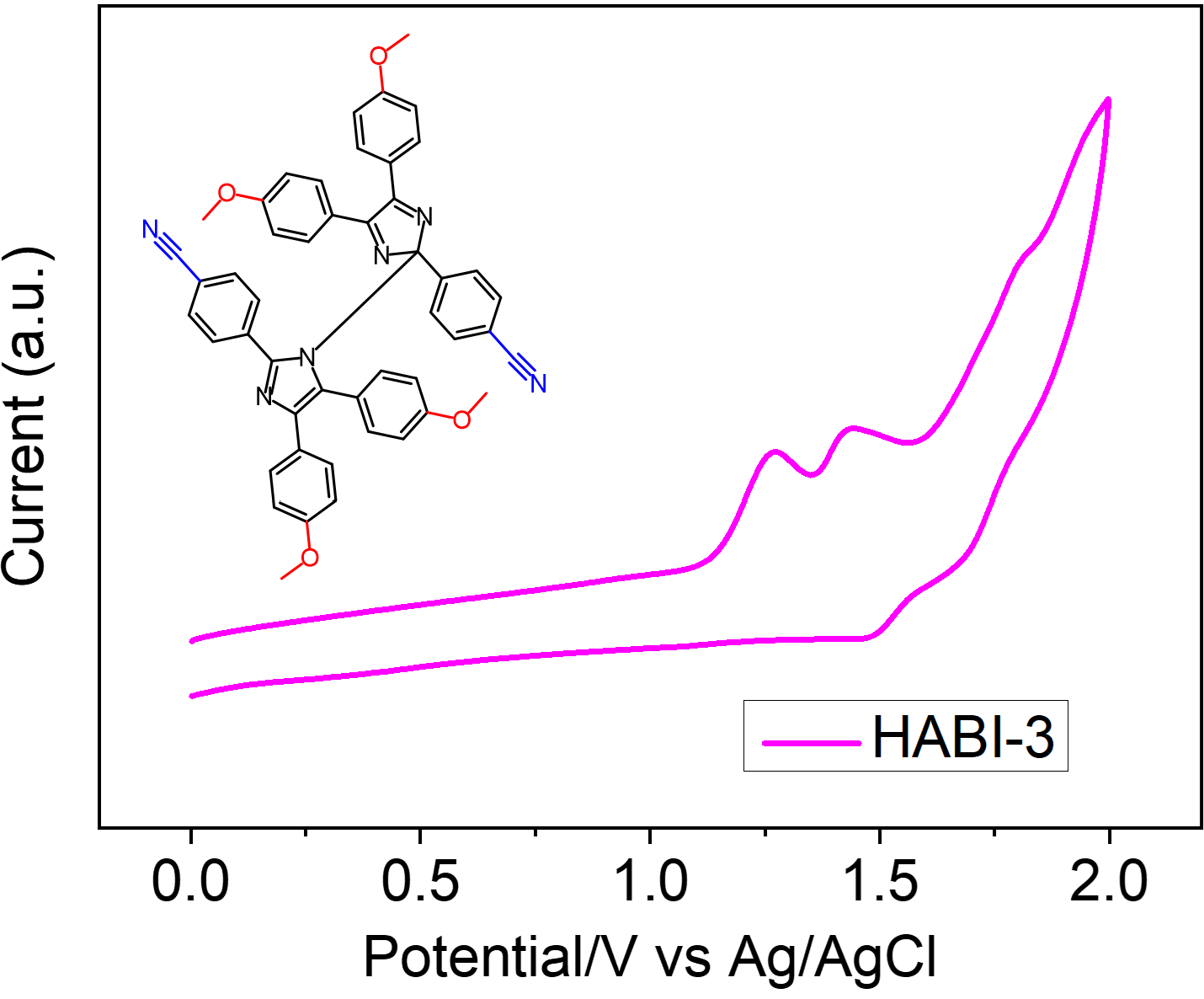


**Figure S4.** The cyclic voltammetry curve of the acetonitrile solution of **HABI-3** (1×10^-3^ mol L^-1^), used to determine the *E*_ox_.


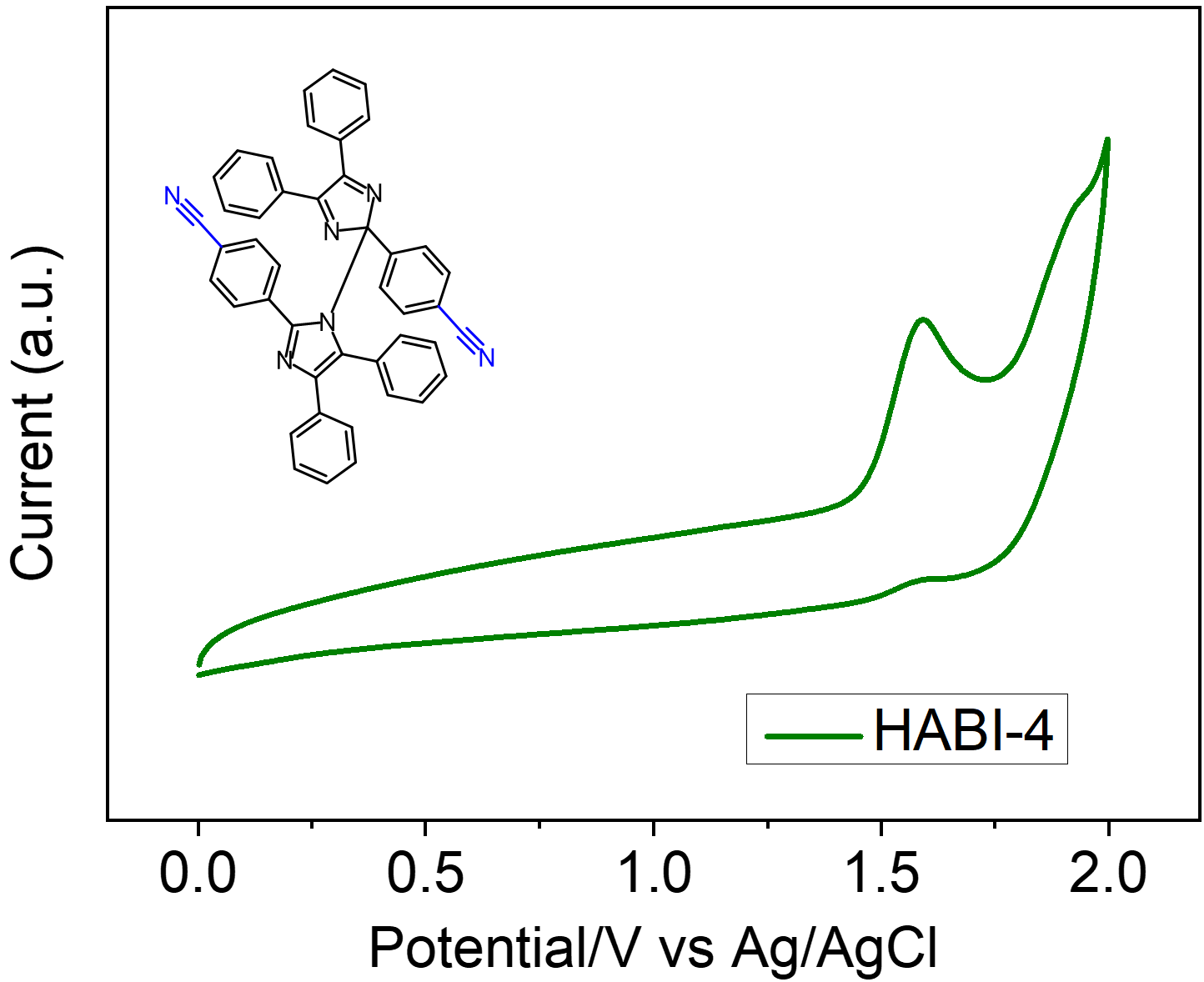


**Figure S5.** The cyclic voltammetry curve of the acetonitrile solution of **HABI-4** (1×10^-3^ mol L^-1^), used to determine the *E*_ox_.


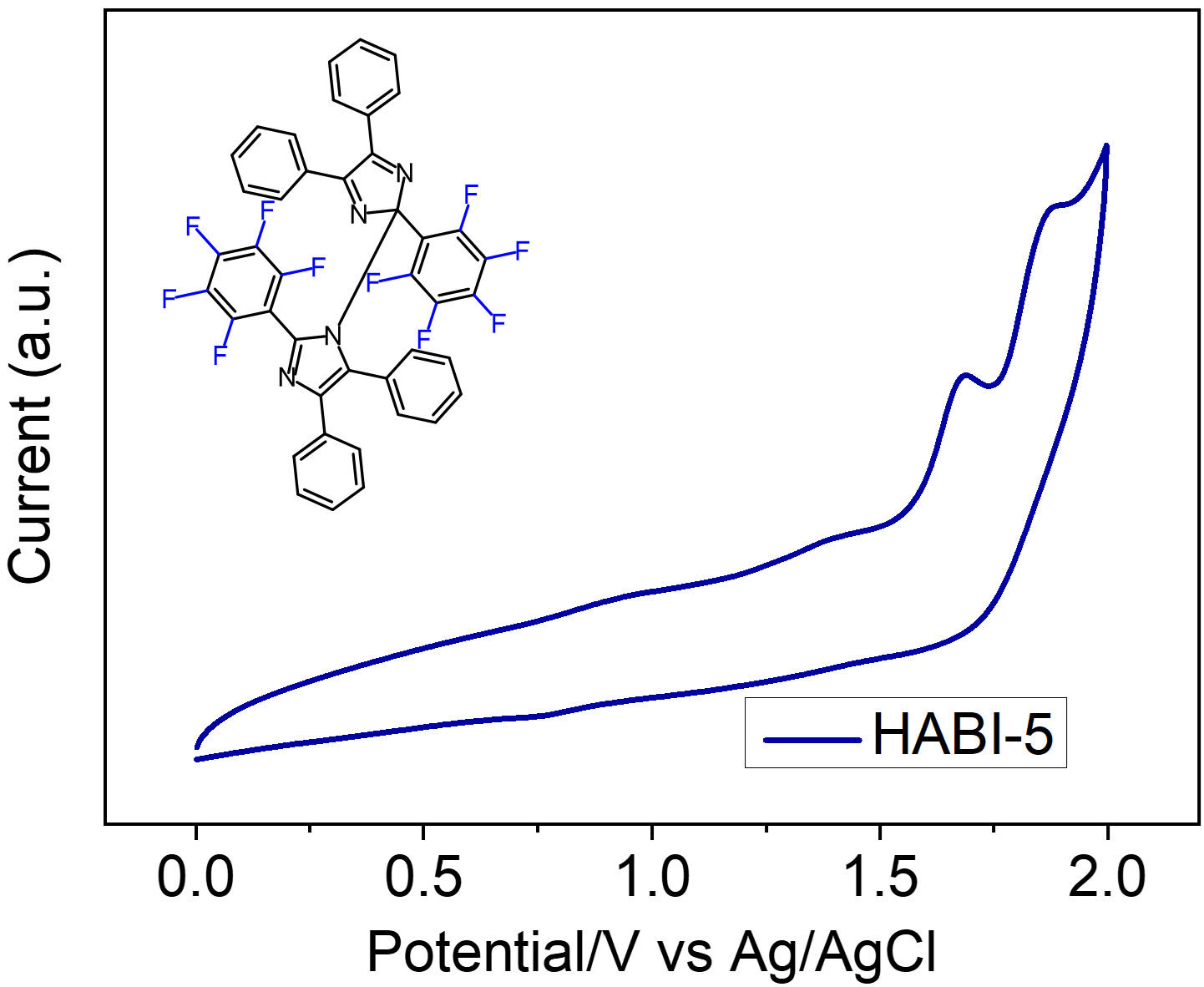


**Figure S6.** The cyclic voltammetry curve of the acetonitrile solution of **HABI-5** (1×10^-3^ mol L^-1^), used to determine the *E*_ox_.


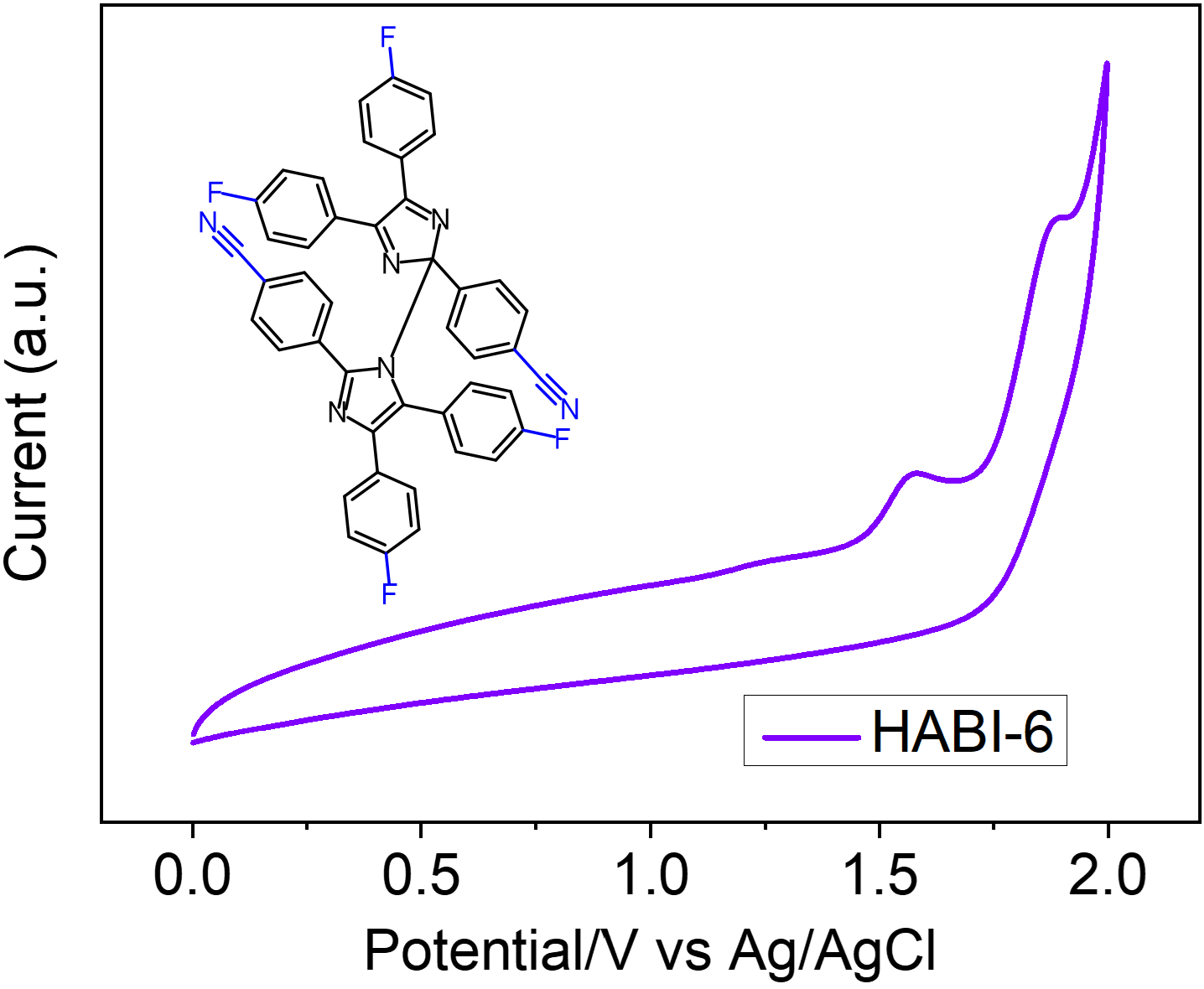


**Figure S7.** The cyclic voltammetry curve of the acetonitrile solution of **HABI-6** (1×10^-3^ mol L^-1^), used to determine the *E*_ox_.


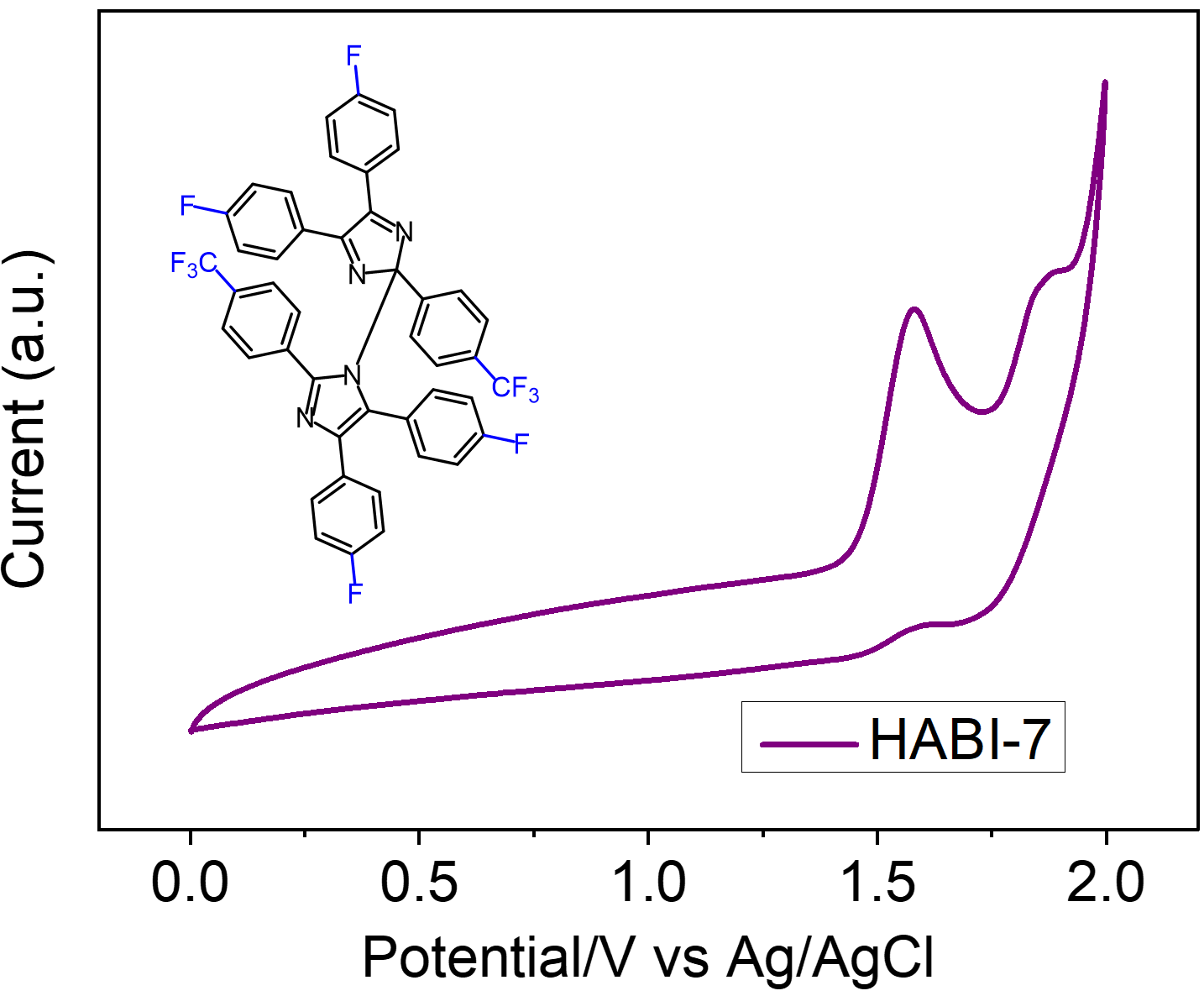


**Figure S8.** The cyclic voltammetry curve of the acetonitrile solution of **HABI-7** (1×10^-3^ mol L^-1^), used to determine the *E*_ox_.


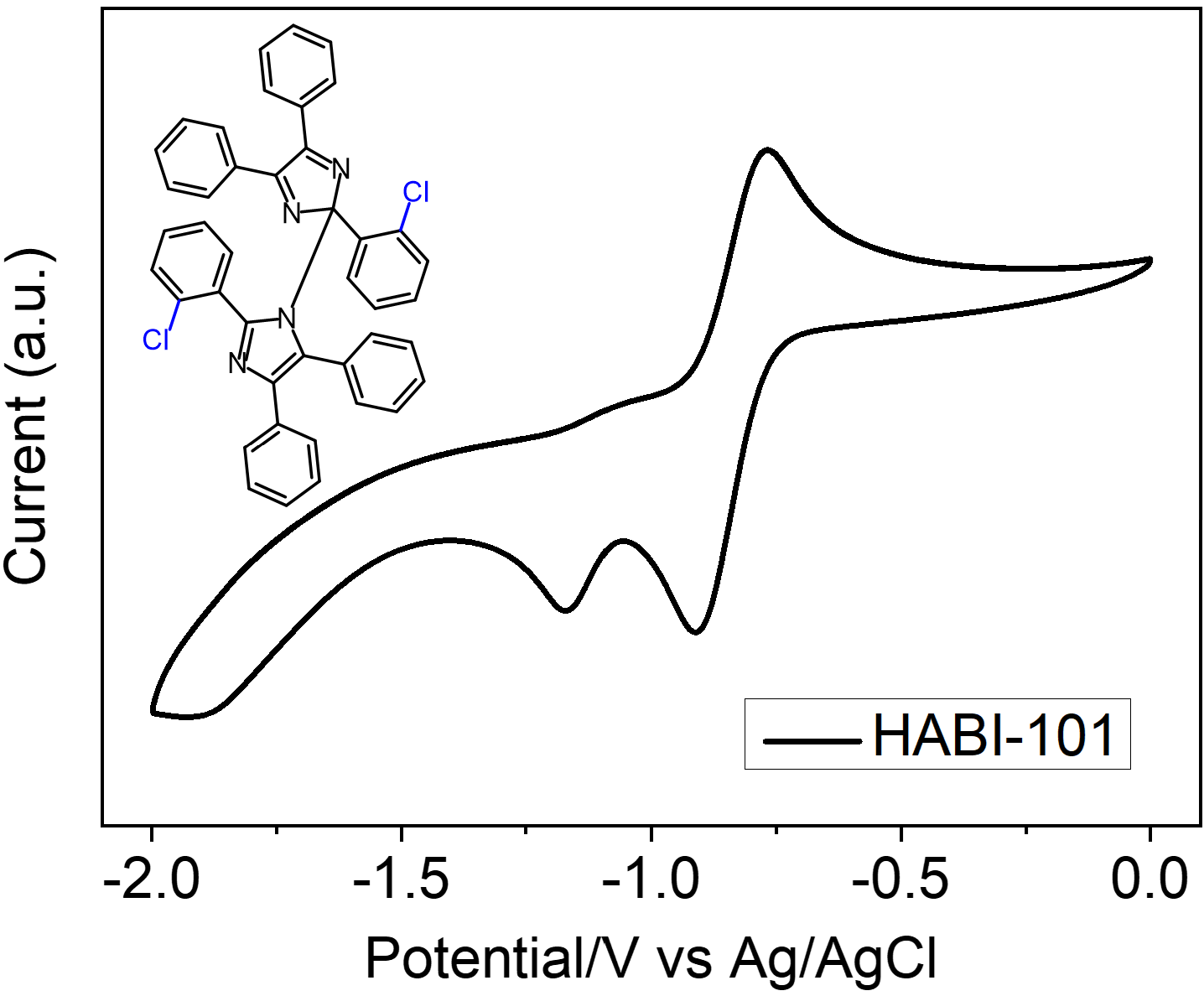


**Figure S9.** The cyclic voltammetry curve of the acetonitrile solution of **HABI-101** (1×10^-3^ mol L^-1^), used to determine the *E*_red_.


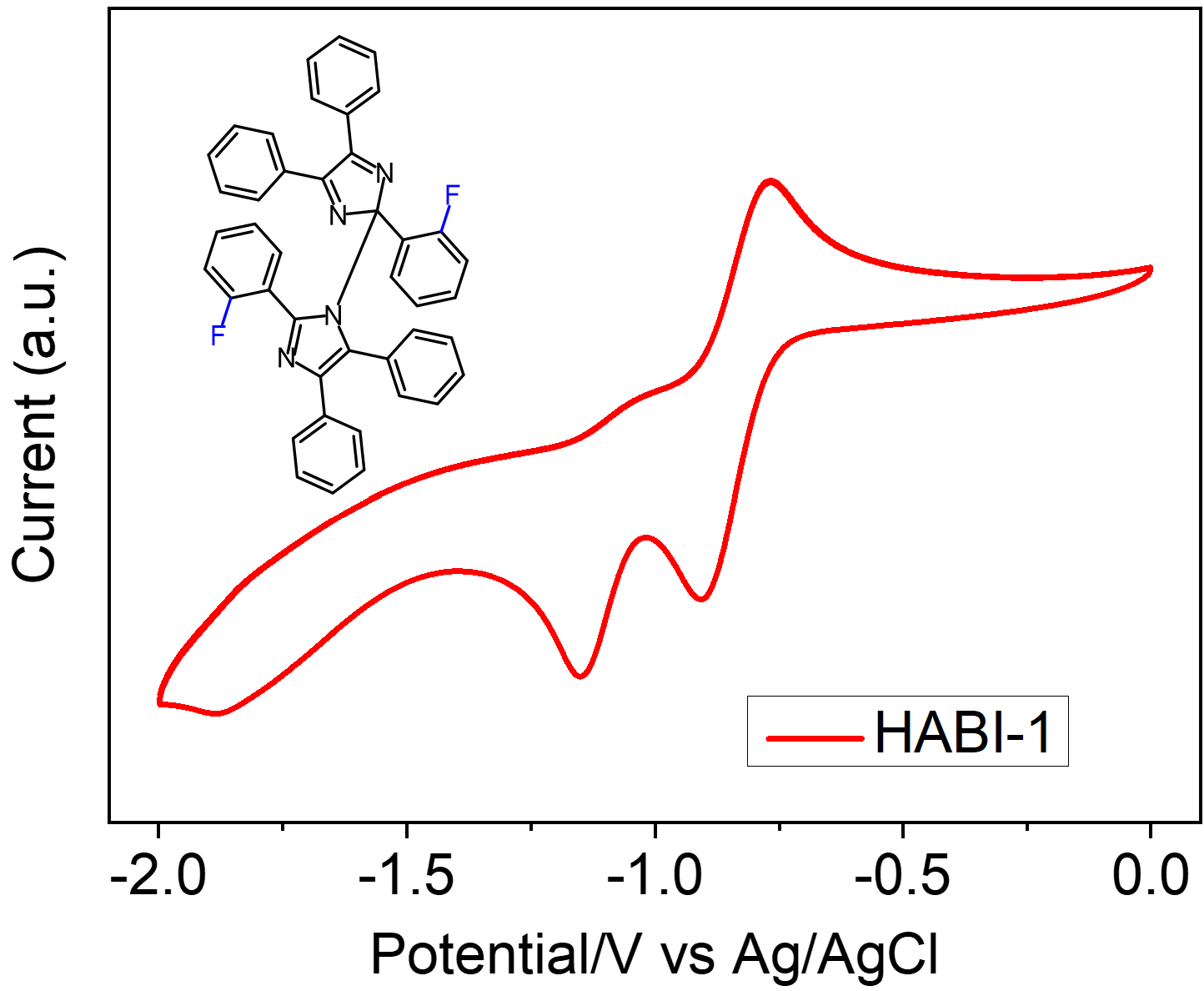


**Figure S10.** The cyclic voltammetry curve of the acetonitrile solution of **HABI-1** (1×10^-3^ mol L^-1^), used to determine the *E*_red_.


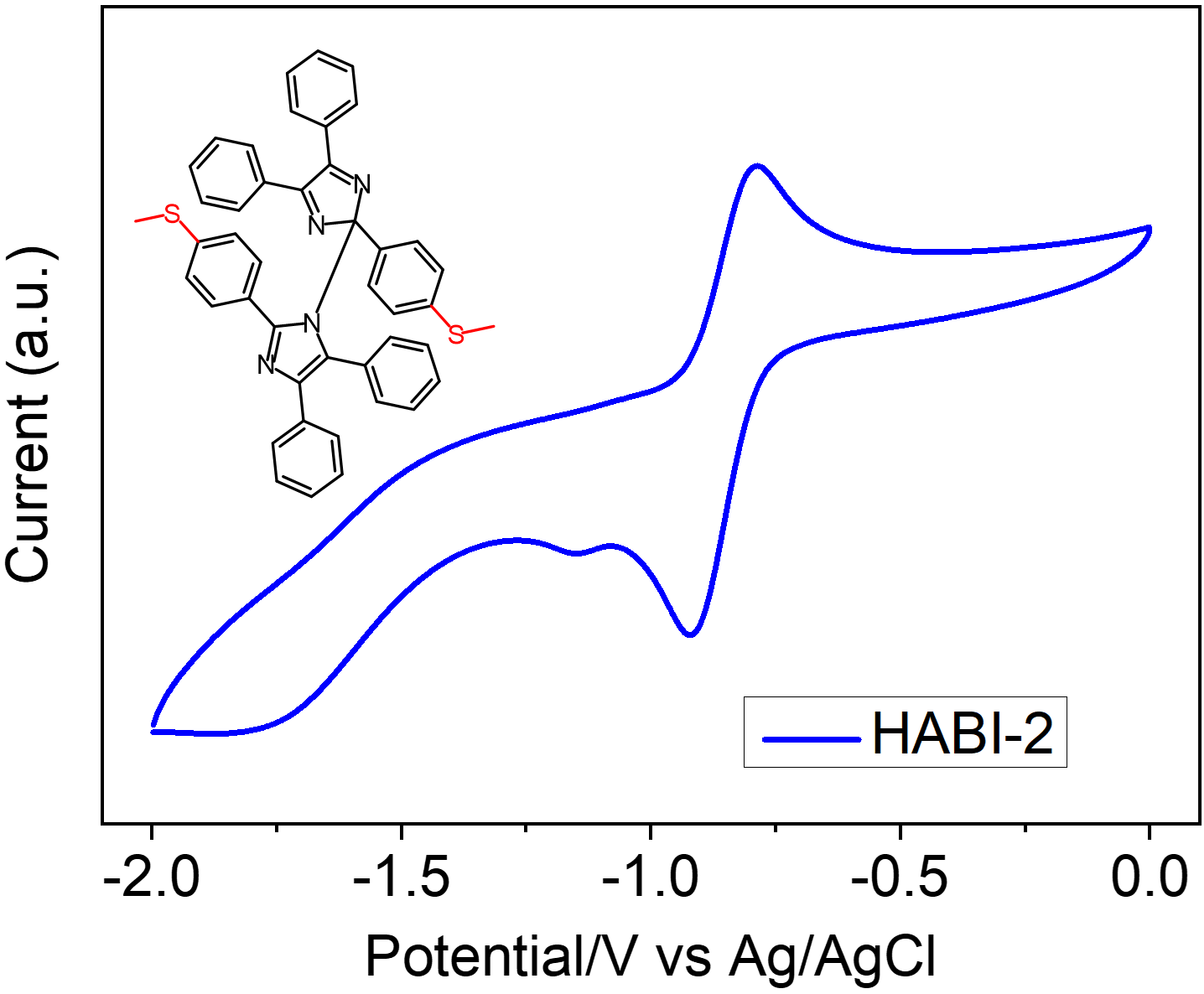


**Figure S11.** The cyclic voltammetry curve of the acetonitrile solution of **HABI-2** (1×10^-3^ mol L^-1^), used to determine the *E*_red_.


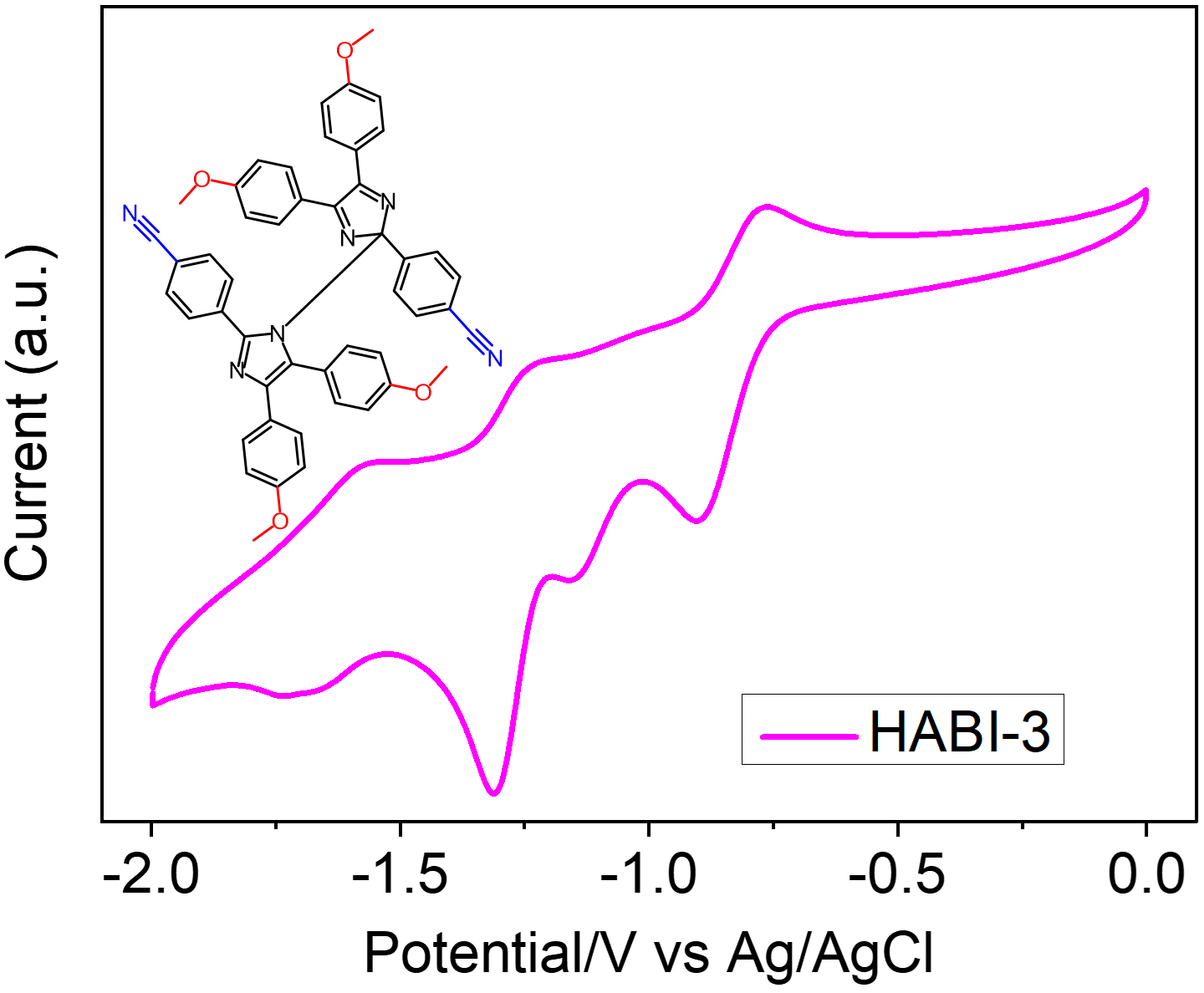


**Figure S12.** The cyclic voltammetry curve of the acetonitrile solution of **HABI-3** (1×10^-3^ mol L^-1^), used to determine the *E*_red_.


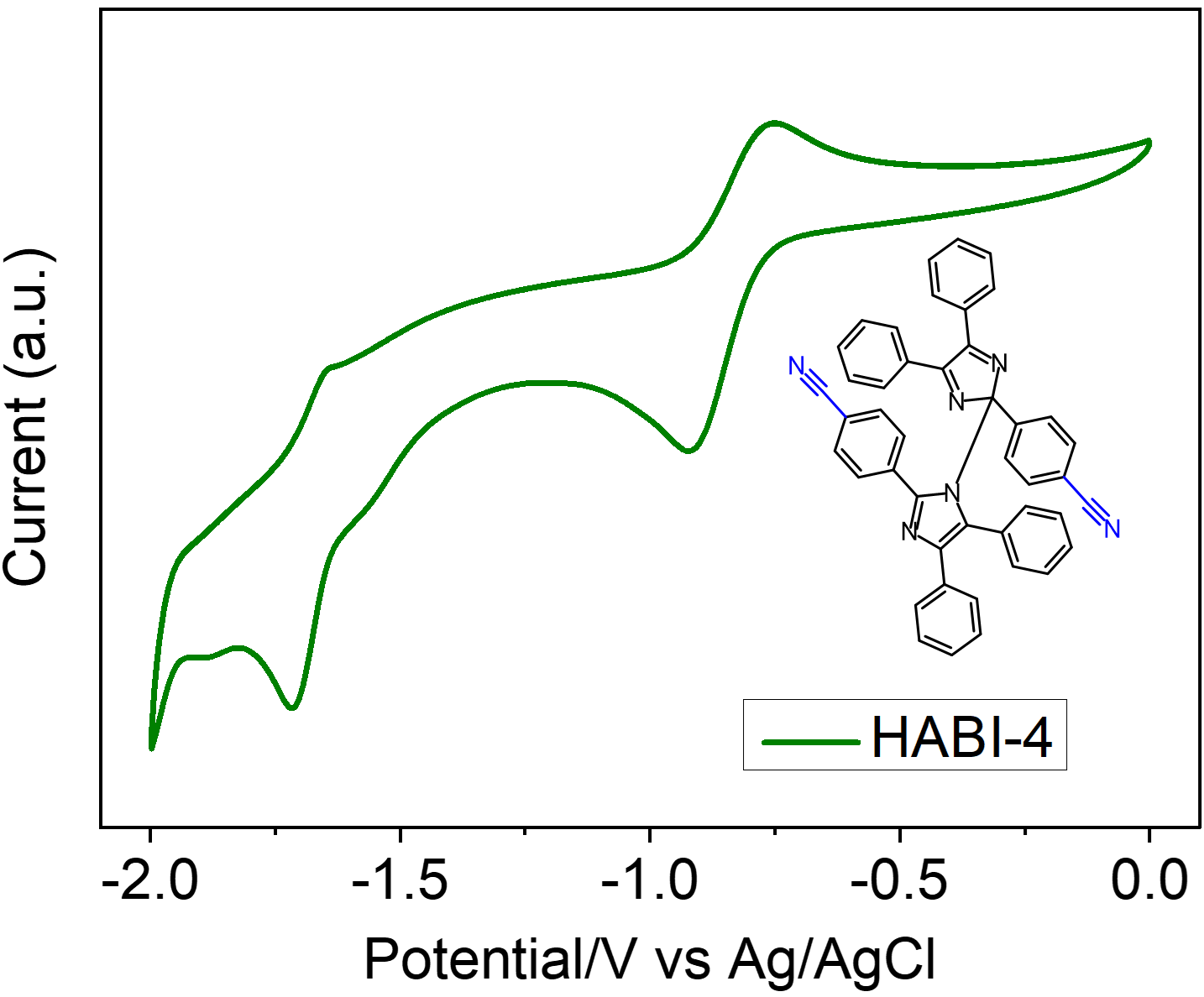


**Figure S13.** The cyclic voltammetry curve of the acetonitrile solution of **HABI-4** (1×10^-3^ mol L^-1^), used to determine the *E*_red_.


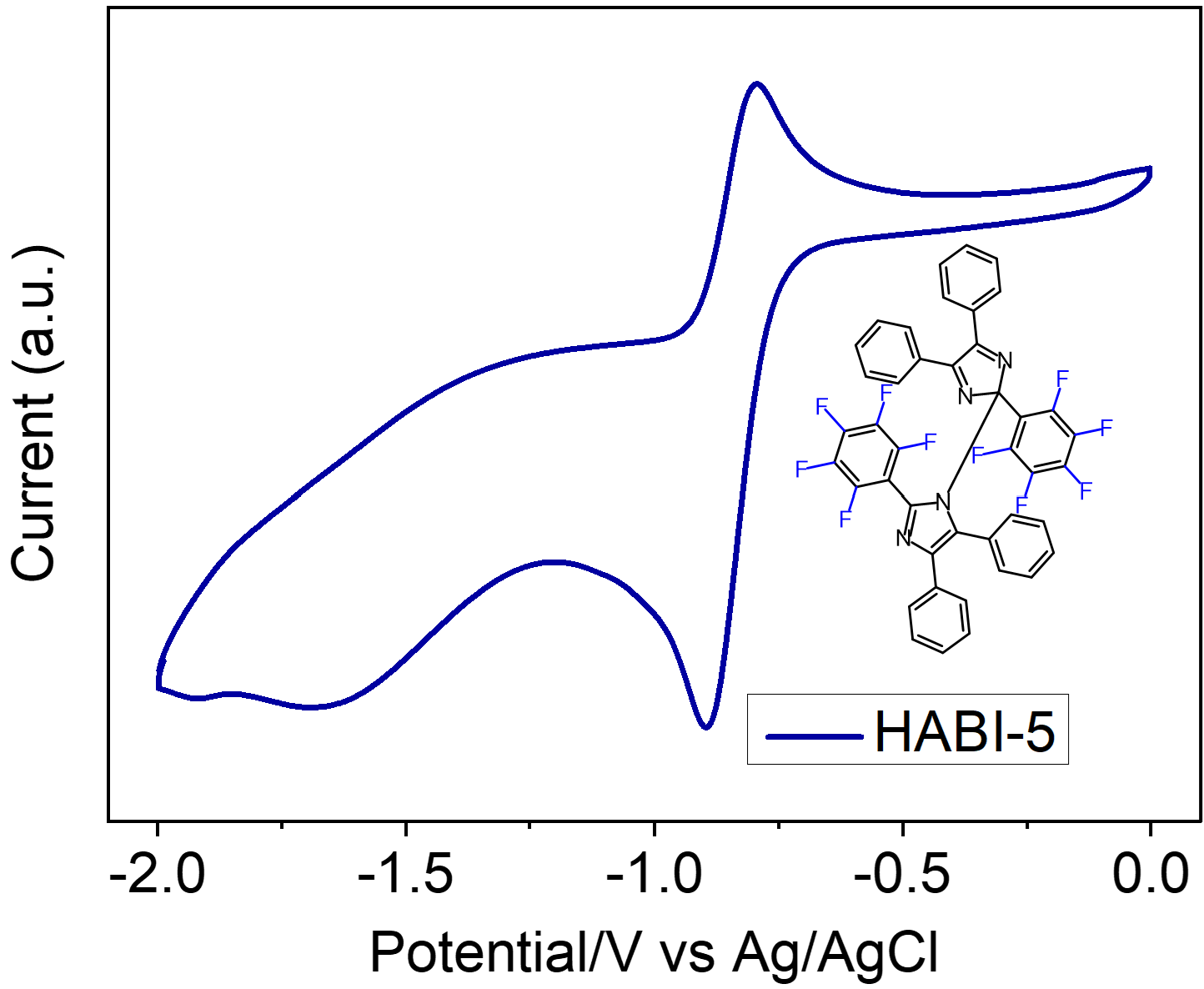


**Figure S14.** The cyclic voltammetry curve of the acetonitrile solution of **HABI-5** (1×10^-3^ mol L^-1^), used to determine the *E*_red_.


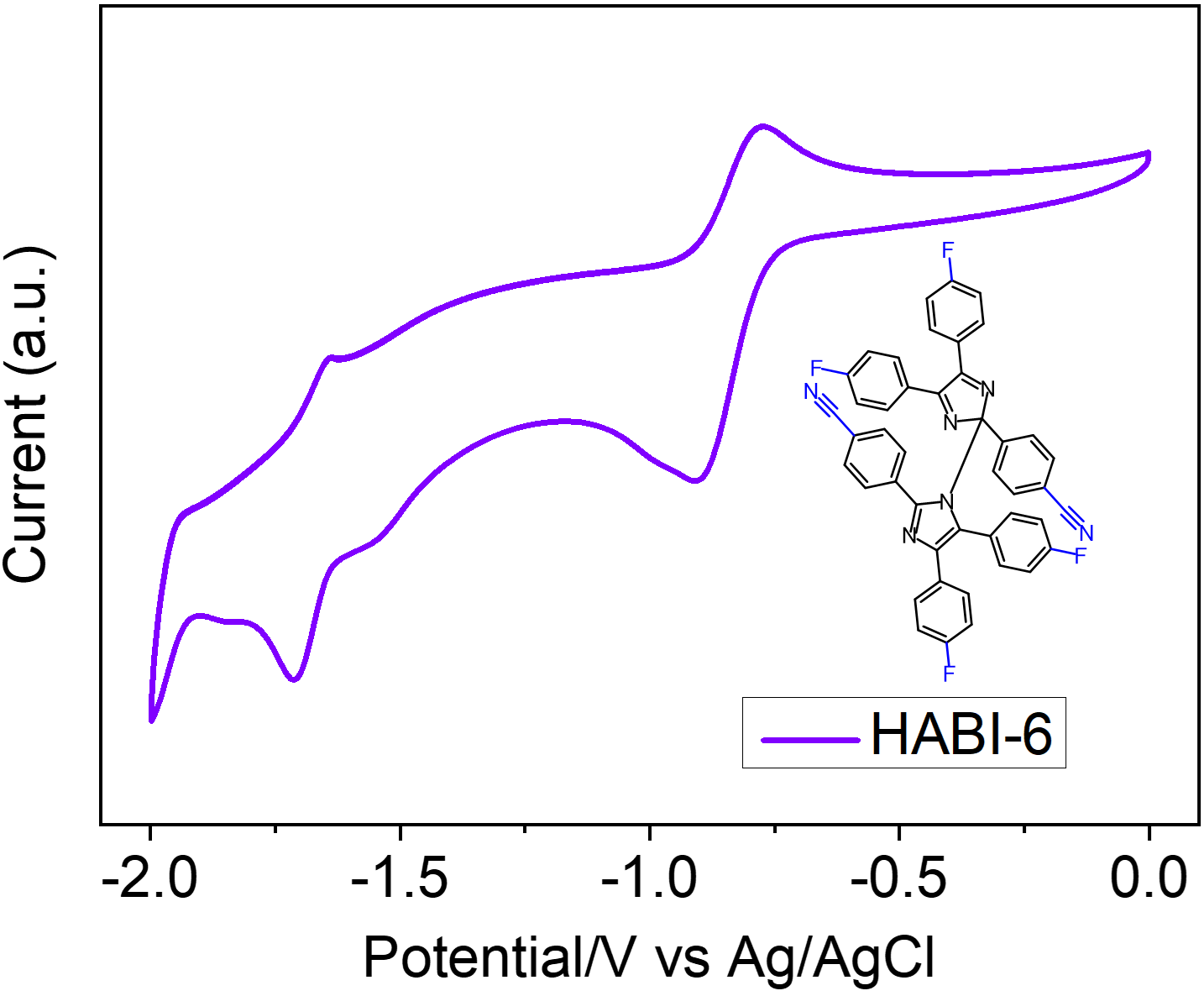


**Figure S15.** The cyclic voltammetry curve of the acetonitrile solution of **HABI-6** (1×10^-3^ mol L^-1^), used to determine the *E*_red_.


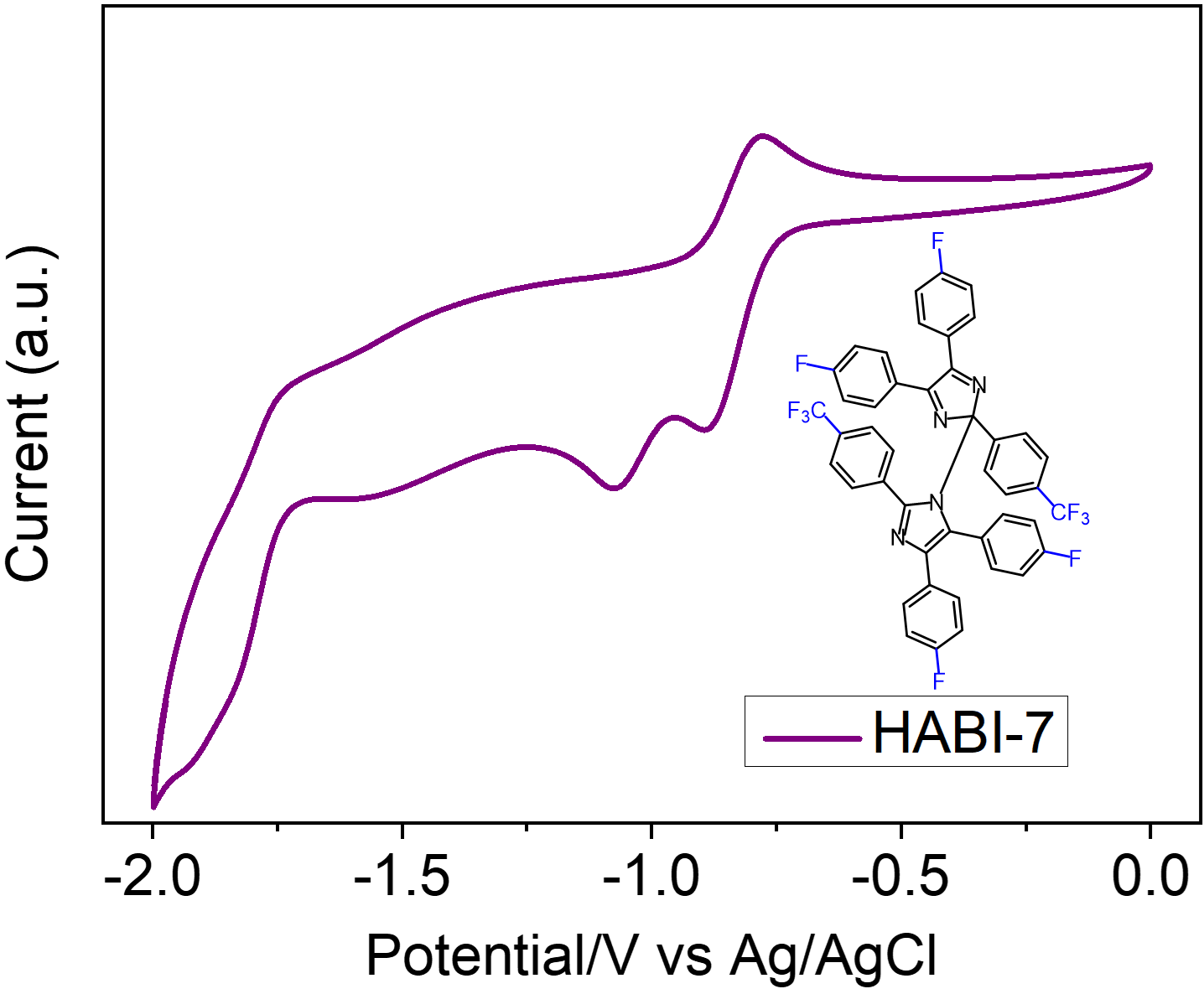


**Figure S16.** The cyclic voltammetry curve of the acetonitrile solution of **HABI-7** (1×10^-3^ mol L^-1^), used to determine the *E*_red_.


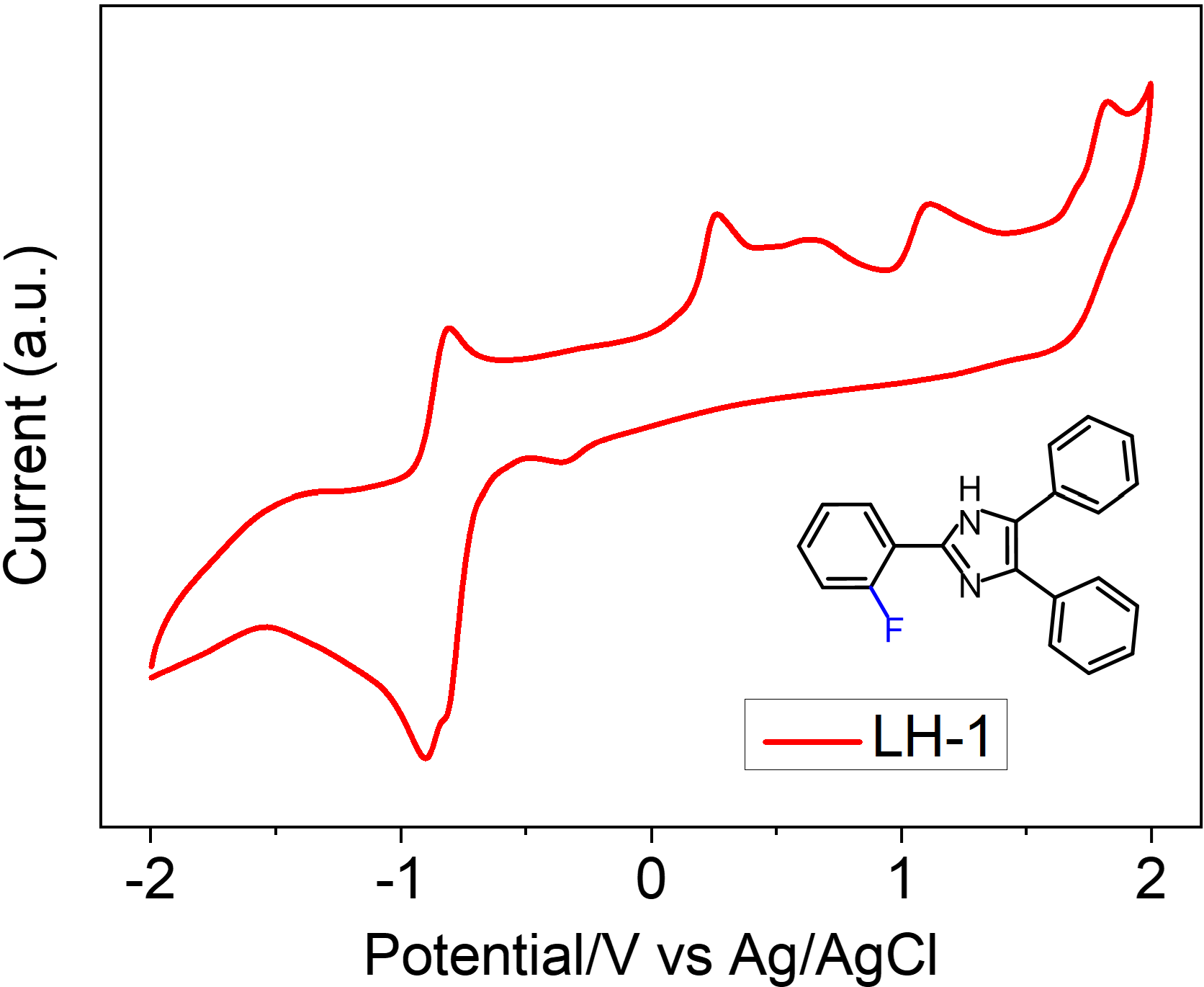


**Figure S17.** The cyclic voltammetry curve of the acetonitrile solution of **LH-1** (1×10^-3^ mol L^-1^), used to determine the *E*_red_ and *E*_ox_.


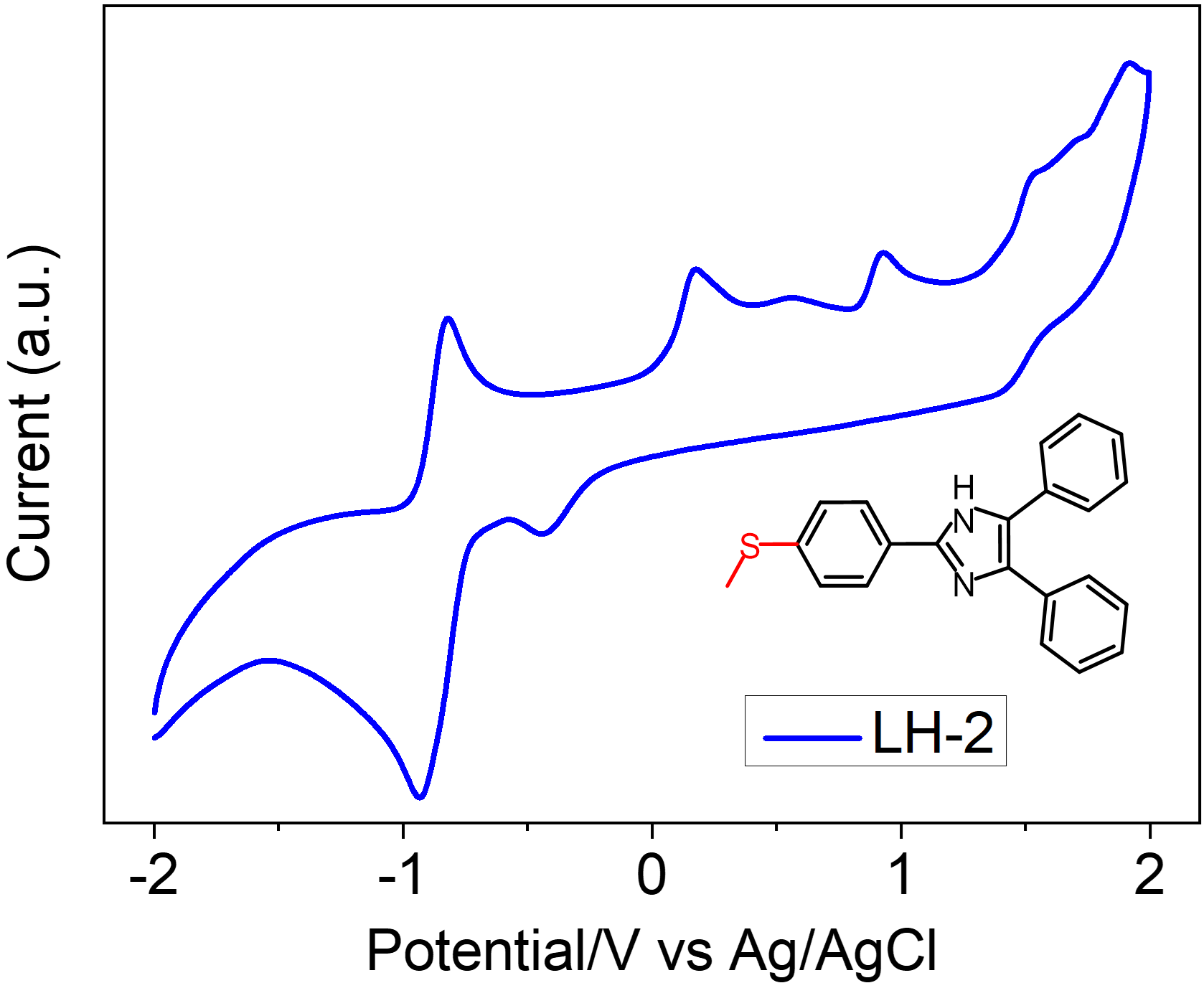


**Figure S18.** The cyclic voltammetry curve of the acetonitrile solution of **LH-2** (1×10^-3^ mol L^-1^), used to determine the *E*_red_ and *E*_ox_.


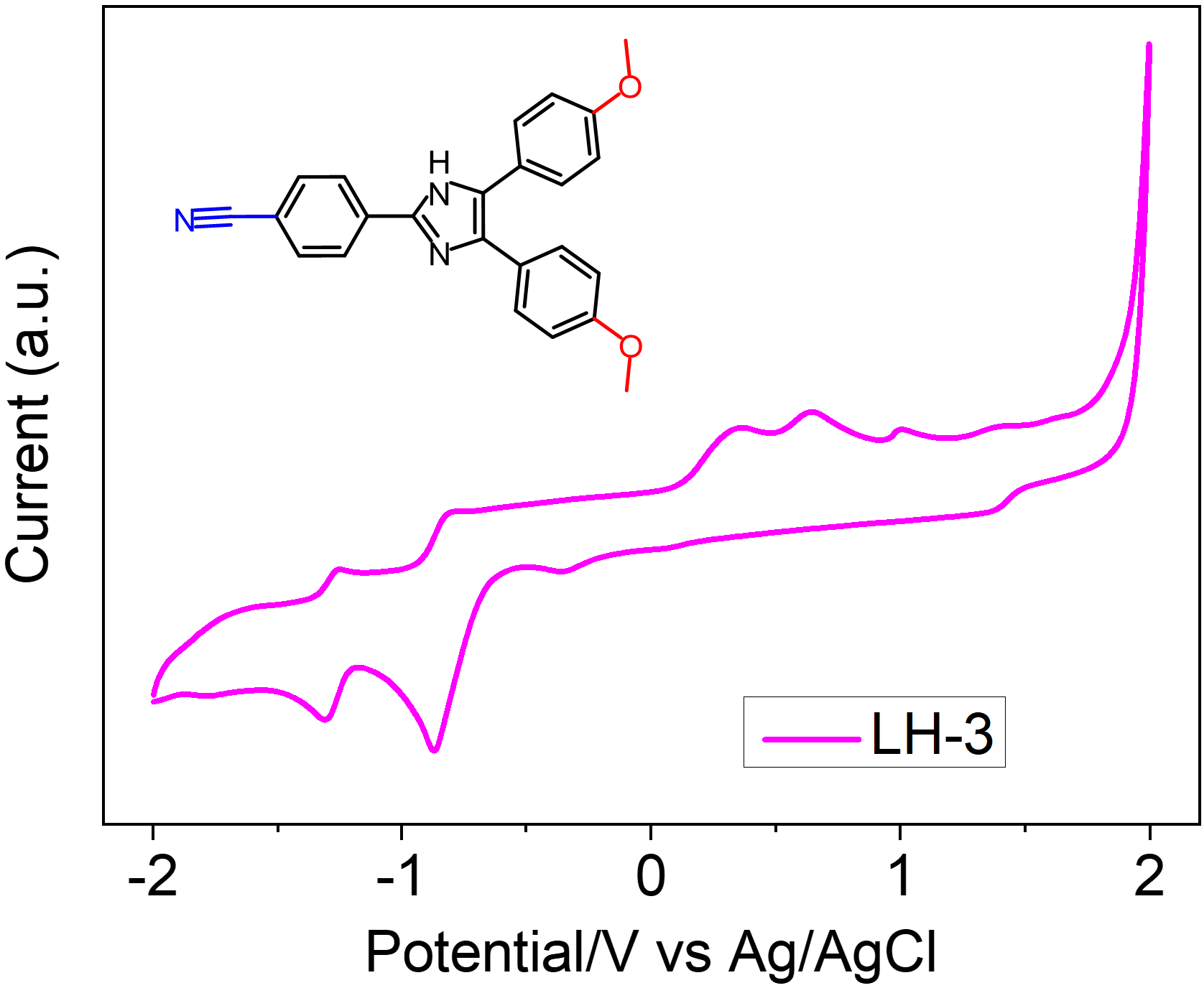


**Figure S19.** The cyclic voltammetry curve of the acetonitrile solution of **LH-3** (1×10^-3^ mol L^-1^), used to determine the *E*_red_ and *E*_ox_.


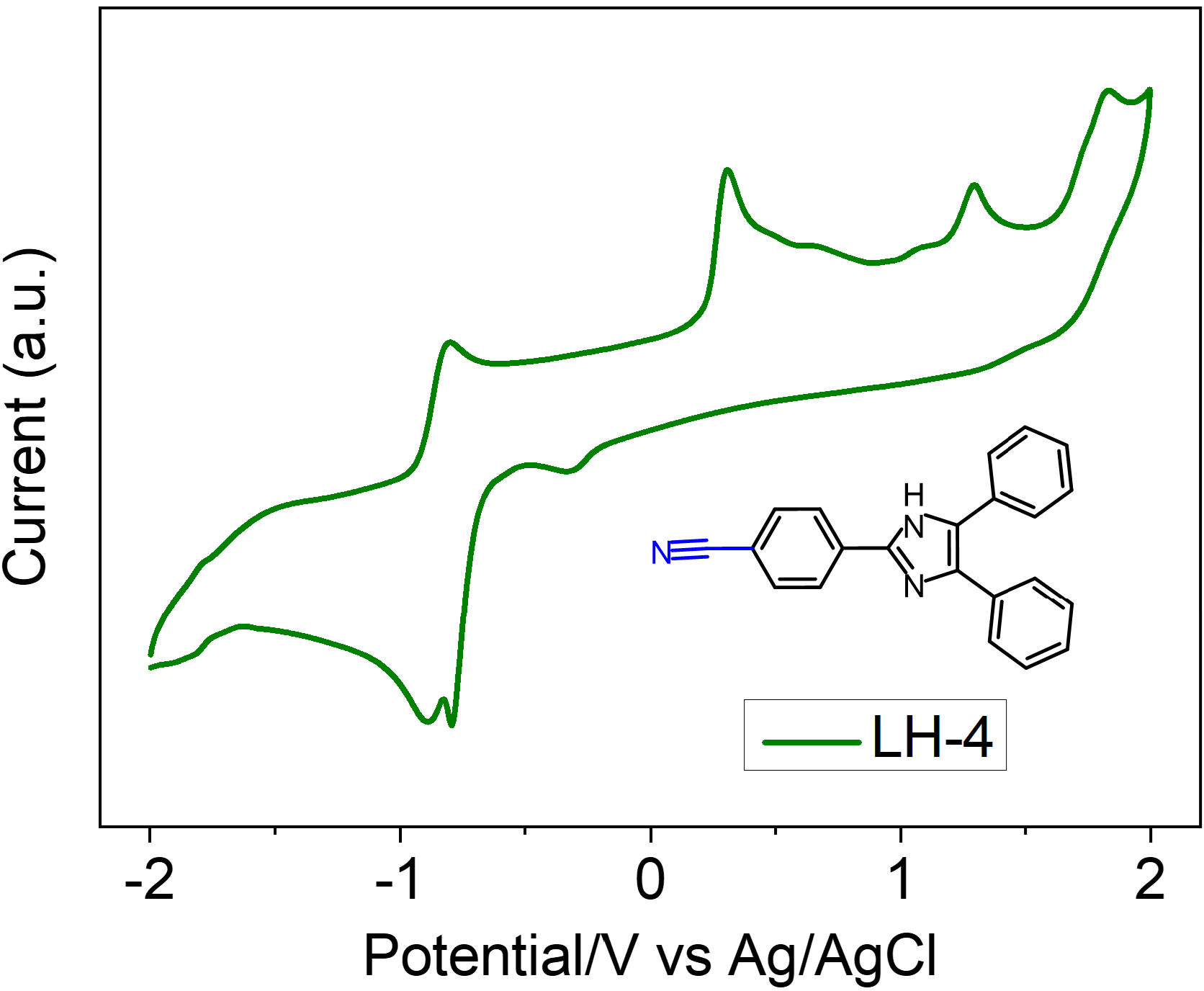


**Figure S20.** The cyclic voltammetry curve of the acetonitrile solution of **LH-4** (1×10^-3^ mol L^-1^), used to determine the *E*_red_ and *E*_ox_.


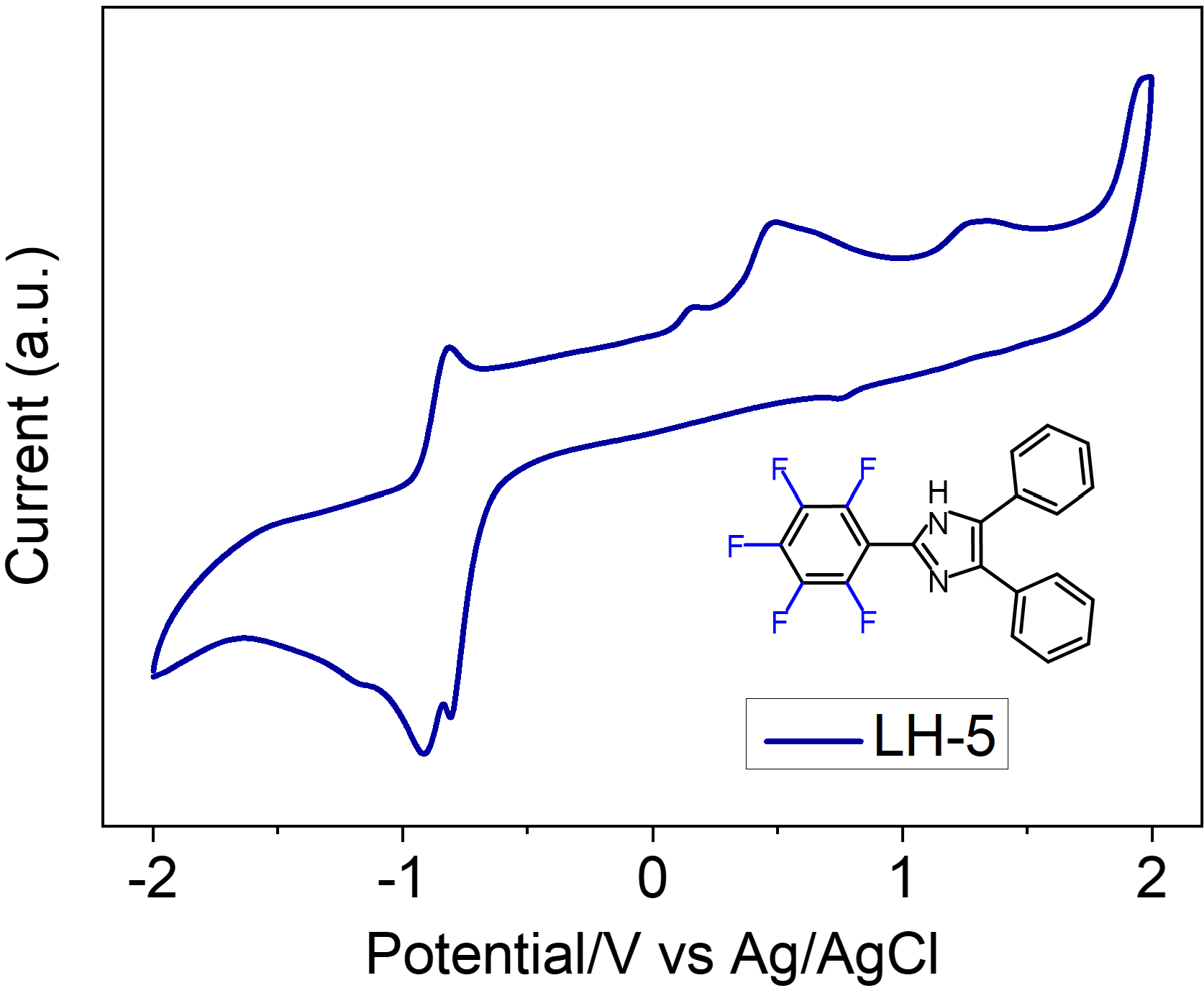


**Figure S21.** The cyclic voltammetry curve of the acetonitrile solution of **LH-5** (1×10^-3^ mol L^-1^), used to determine the *E*_red_ and *E*_ox_.


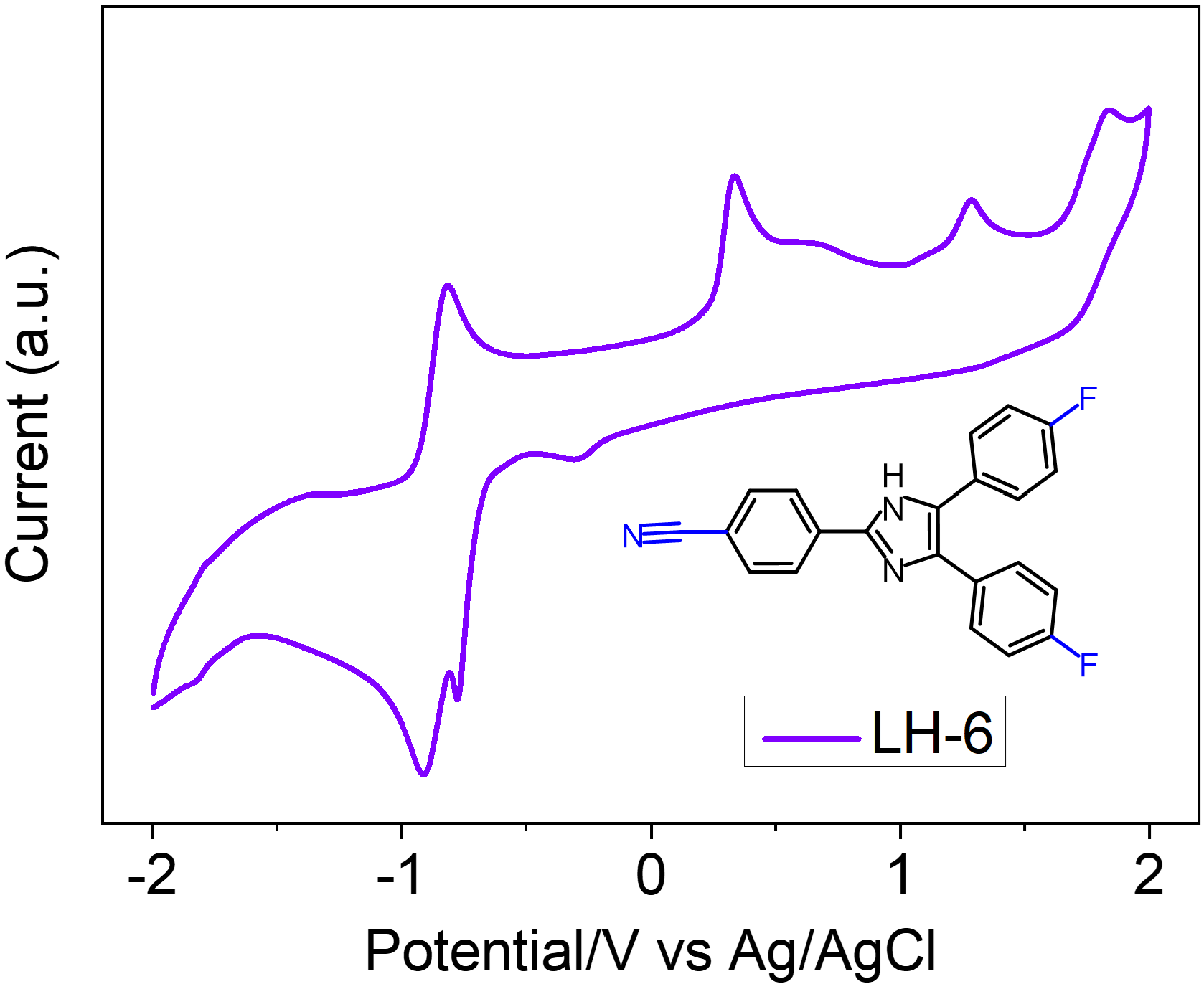


**Figure S22.** The cyclic voltammetry curve of the acetonitrile solution of **LH-6** (1×10^-3^ mol L^-1^), used to determine the *E*_red_ and *E*_ox_.


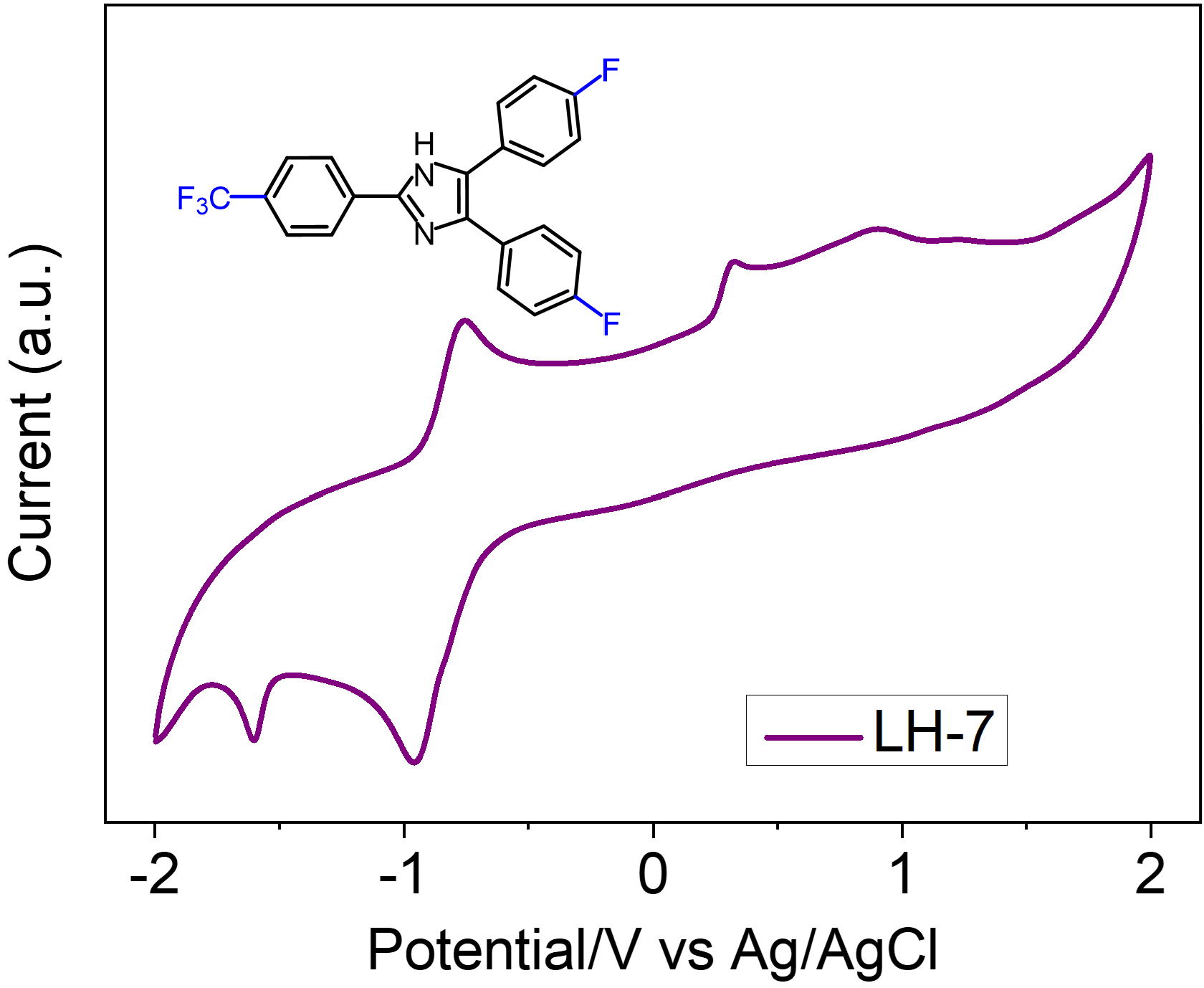


**Figure S23.** The cyclic voltammetry curve of the acetonitrile solution of **LH-7** (1×10^-3^ mol L^-1^), used to determine the *E*_red_ and *E*_ox_.

## Optical Properties of Sens and HABIs


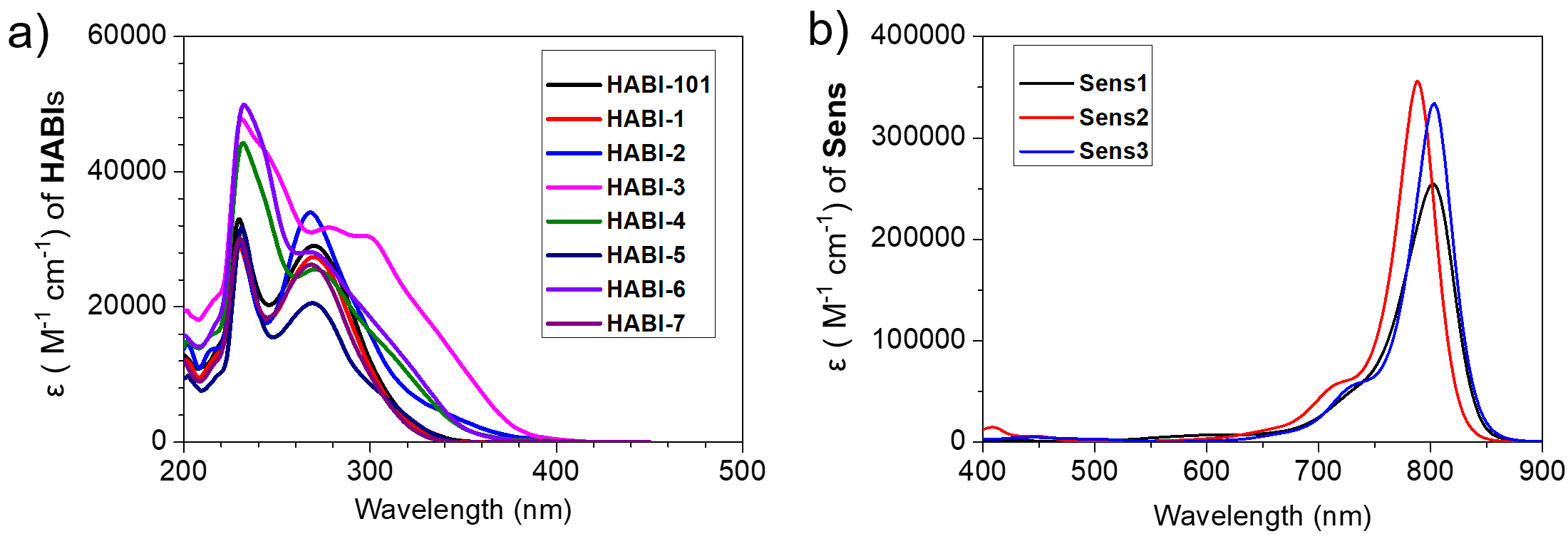


**Figure S24**. Absorption curves of (a) **HABI**s and (b) **Sens** in anhydrous dichloromethane (1×10^-5^ mol L^-1^).

## Photopolymerization Experiments


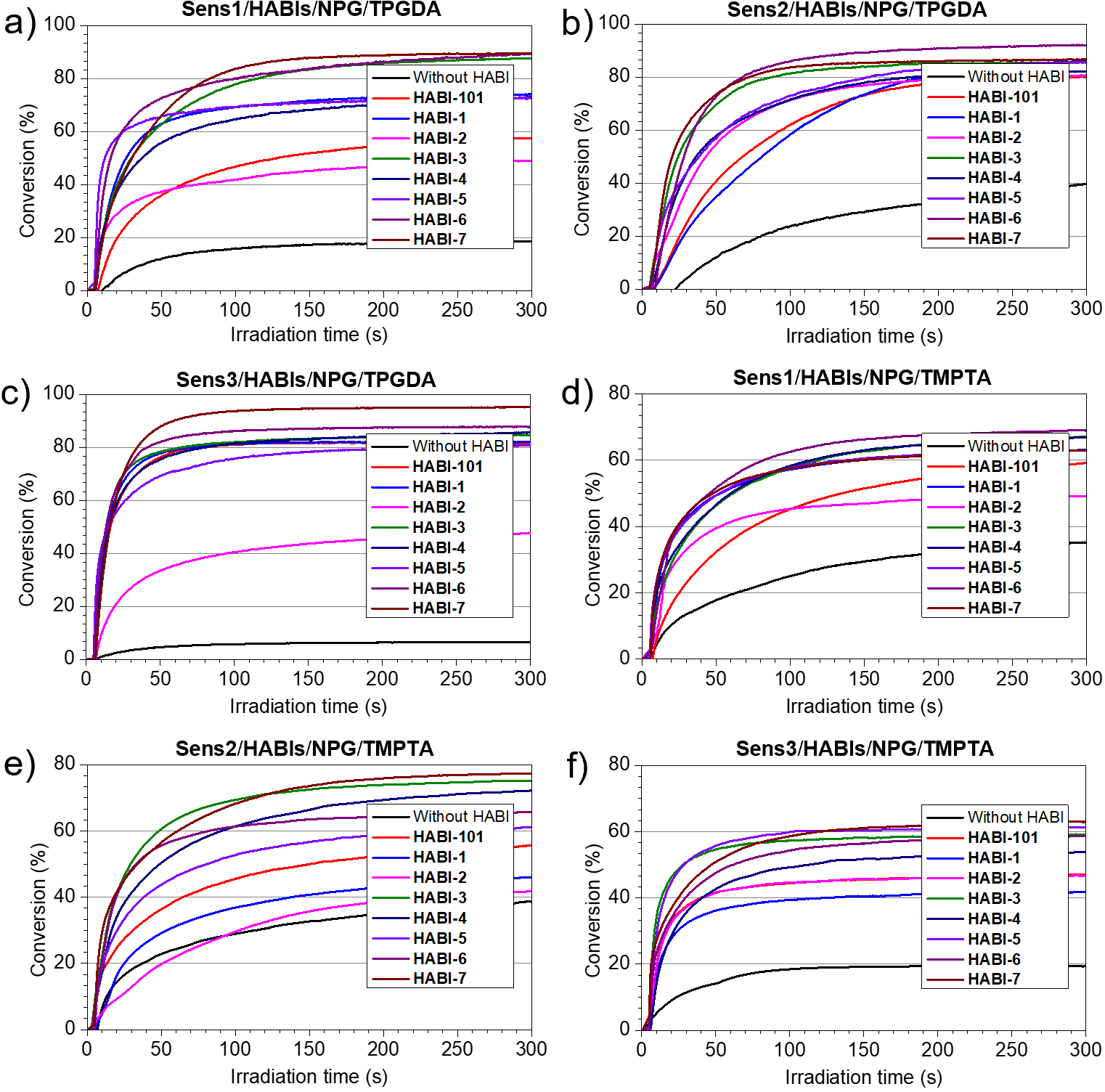


**Figure S25**. Double bond conversion of **TPGDA** and **TMPTA** with different **Sens** (0.2 mol% to **monomer**s) and **HABI**s (1 mol% to **monomer**s) in the presence of the **NPG** (2 mol% to **monomer**s) applying a NIR laser source emitting at 808 nm with an excitation density of 714 mW·cm^-2^; (a) **Sens1/HABI**s**/NPG/TPGDA** systems, (b) **Sens2/HABI**s**/NPG/TPGDA** systems, (c) **Sens3/HABI**s**/NPG/TPGDA** systems, (d) **Sens1/HABI**s**/NPG/TMPTA** systems, (e) **Sens2/HABI**s**/NPG/TMPTA** systems, (f) **Sens3/HABI**s**/NPG/TMPTA** systems.

**Table S2** Final double bond conversions of **TPGDA** and **TMPTA** with different **Sens** (0.2 mol% to **monomer**s) and **HABI**s (1 mol% to **monomer**s) in the presence of the **NPG** (2 mol% to **monomer**s) applying a NIR laser source emitting at 808 nm with an excitation density of 714 mW·cm^-2^ (exposure time: 5 min).

| **HABI**s | Final double bond conversions (%) | | | | | |
| --- | --- | --- | --- | --- | --- | --- |
|  | **Sens**/**HABI**s/**NPG**/**TPGDA** | | | **Sens**/**HABI**s/**NPG**/**TMPTA** | | |
|  | **Sens1** | **Sens2** | **Sens3** | **Sens1** | **Sens2** | **Sens3** |
| Without **HABI**s | 18.5 | 39.6 | 6.5 | 35.2 | 38.4 | 19.4 |
| **HABI-101** | 57.6 | 80.1 | 81.4 | 59.2 | 55.6 | 47.0 |
| **HABI-1** | 74.0 | 85.9 | 82.1 | 63.1 | 46.1 | 41.8 |
| **HABI-2** | 49.0 | 80.6 | 47.7 | 49.2 | 41.7 | 46.7 |
| **HABI-3** | 87.6 | 85.8 | 84.7 | 67.1 | 75.2 | 59.2 |
| **HABI-4** | 73.1 | 82.2 | 85.7 | 66.9 | 72.1 | 53.9 |
| **HABI-5** | 72.6 | 85.6 | 80.8 | 63.0 | 61.2 | 61.4 |
| **HABI-6** | 89.4 | 92.1 | 87.7 | 68.9 | 65.2 | 58.7 |
| **HABI-7** | 89.5 | 86.7 | 95.3 | 63.0 | 77.4 | 63.0 |


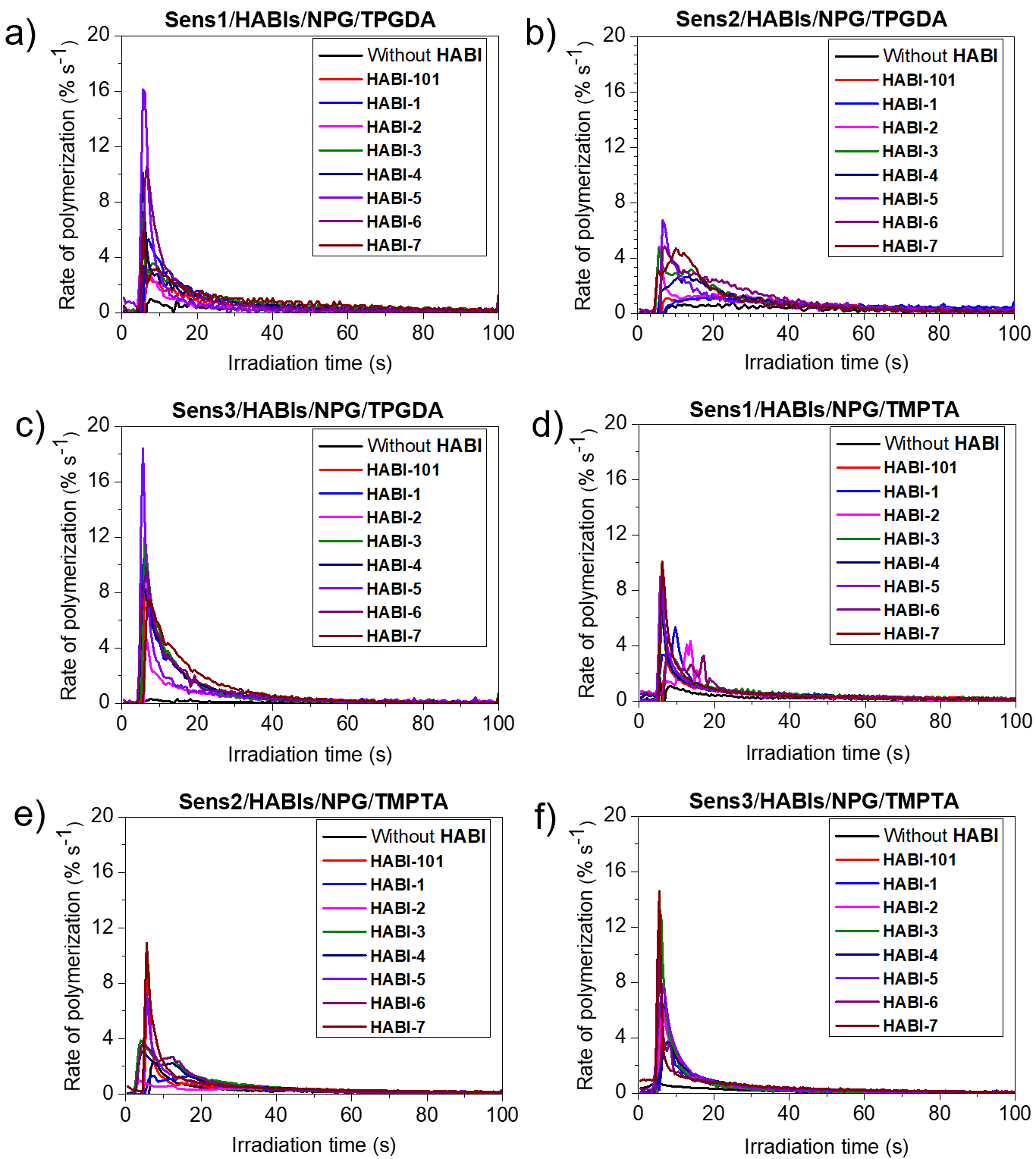


**Figure S26**. Double bond conversion of **TPGDA** and **TMPTA** with different **Sens** (0.2 mol% to **monomers**) and **HABI**s (1 mol% to **monomer**s) in the presence of the **NPG** (2 mol% to **monomer**s) applying a NIR laser source emitting at 808 nm with an excitation density of 714 mW·cm^-2^; (a) **Sens1/HABI**s**/NPG/TPGDA** systems, (b) **Sens2/HABI**s**/NPG/TPGDA** systems, (c) **Sens3/HABI**s**/NPG/TPGDA** systems, (d) **Sens1/HABI**s**/NPG/TMPTA** systems, (e) **Sens2/HABI**s**/NPG/TMPTA** systems, (f) **Sens3/HABI**s**/NPG/TMPTA** systems.

**Table S3** Rate maximums of photopolymerization of **TPGDA** and **TMPTA** with different **Sens** (0.2 mol% to **monomer**s) and **HABIs** (1 mol% to **monomer**s) in the presence of the **NPG** (2 mol% to **monomer**s) applying a NIR laser source emitting at 808 nm with an excitation density of 714 mW·cm^-2^ (exposure time: 5 min).

| **HABI**s | Rate maximums of photopolymerization (% s^-1^) | | | | | |
| --- | --- | --- | --- | --- | --- | --- |
|  | **Sens**/**HABI**s/**NPG**/**TPGDA** | | | **Sens**/**HABI**s/**NPG**/**TMPTA** | | |
|  | **Sens1** | **Sens2** | **Sens1** | **Sens2** | **Sens1** | **Sens2** |
| Without **HABI**s | 0.98 | 0.55 | 0.29 | 1.07 | 2.31 | 0.74 |
| **HABI-101** | 2.44 | 1.32 | 9.59 | 2.74 | 8.88 | 5.85 |
| **HABI-1** | 5.28 | 1.16 | 10.94 | 5.35 | 1.33 | 6.83 |
| **HABI-2** | 5.85 | 4.80 | 4.60 | 4.32 | 1.01 | 6.58 |
| **HABI-3** | 7.06 | 4.67 | 12.14 | 6.36 | 3.86 | 13.45 |
| **HABI-4** | 10.08 | 2.60 | 8.46 | 7.30 | 3.06 | 3.66 |
| **HABI-5** | 16.15 | 6.72 | 18.41 | 9.08 | 7.32 | 7.54 |
| **HABI-6** | 10.52 | 4.83 | 9.81 | 4.62 | 3.65 | 10.10 |
| **HABI-7** | 5.89 | 4.67 | 7.46 | 10.05 | 10.92 | 14.61 |


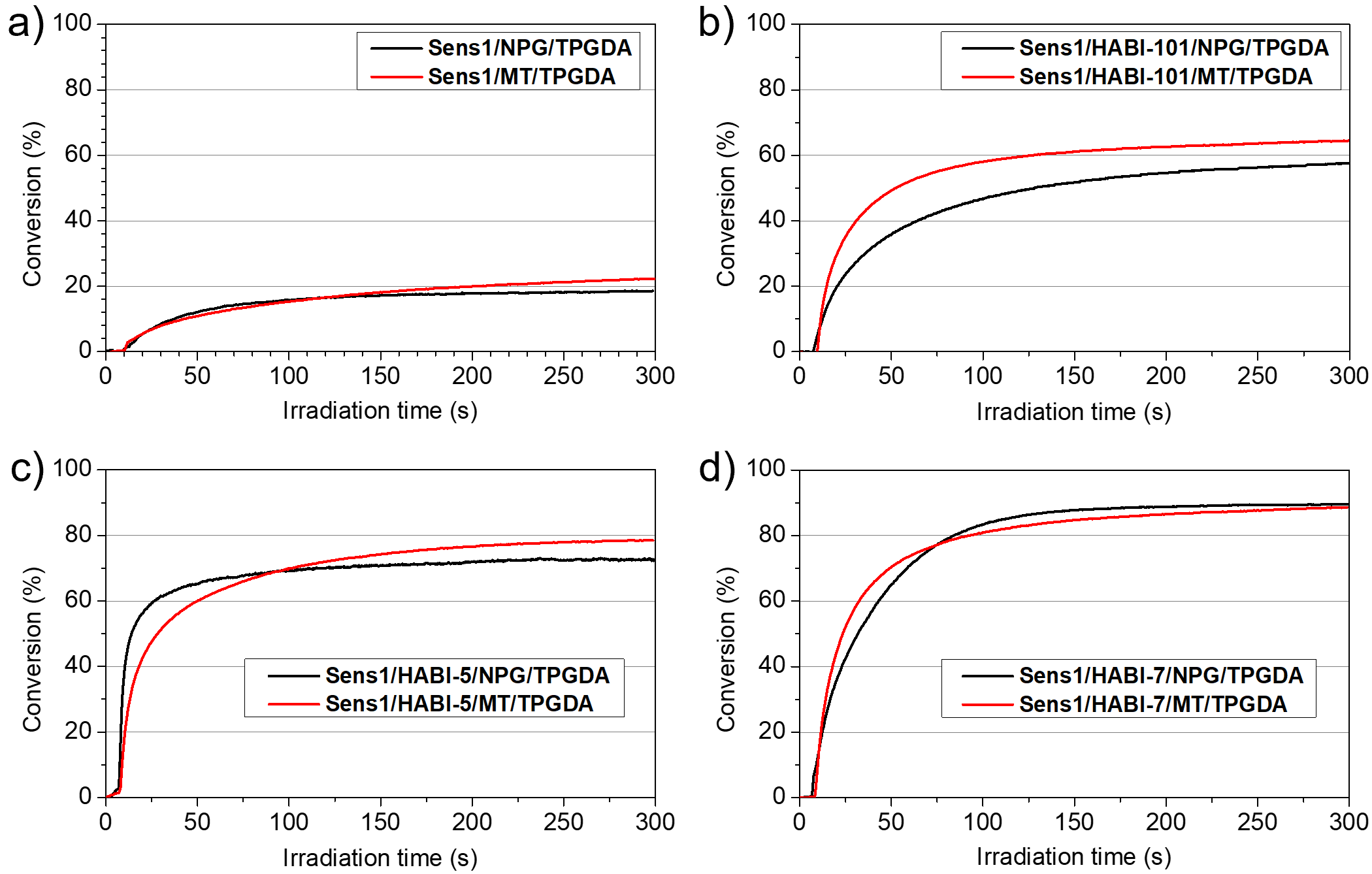


**Figure S27**. Double bond conversion of **TPGDA** with **Sens1** (0.2 mol% to **TPGDA**) and **HABI**s (1 mol% to **monomer**s) in the presence of the donors (**NPG** or **MT**, 2 mol% to **TPGDA**) applying a NIR laser source emitting at 808 nm with an excitation density of 714 mW·cm^-2^; (a) **Sens1/donor**s**/TPGDA** systems, (b) **Sens1/HABI-101/**donors**/TPGDA** systems, (c) **Sens1/HABI-5/donor**s**/TPGDA** systems, (d) **Sens1/HABI-7/donor**s**/TMPTA** systems.


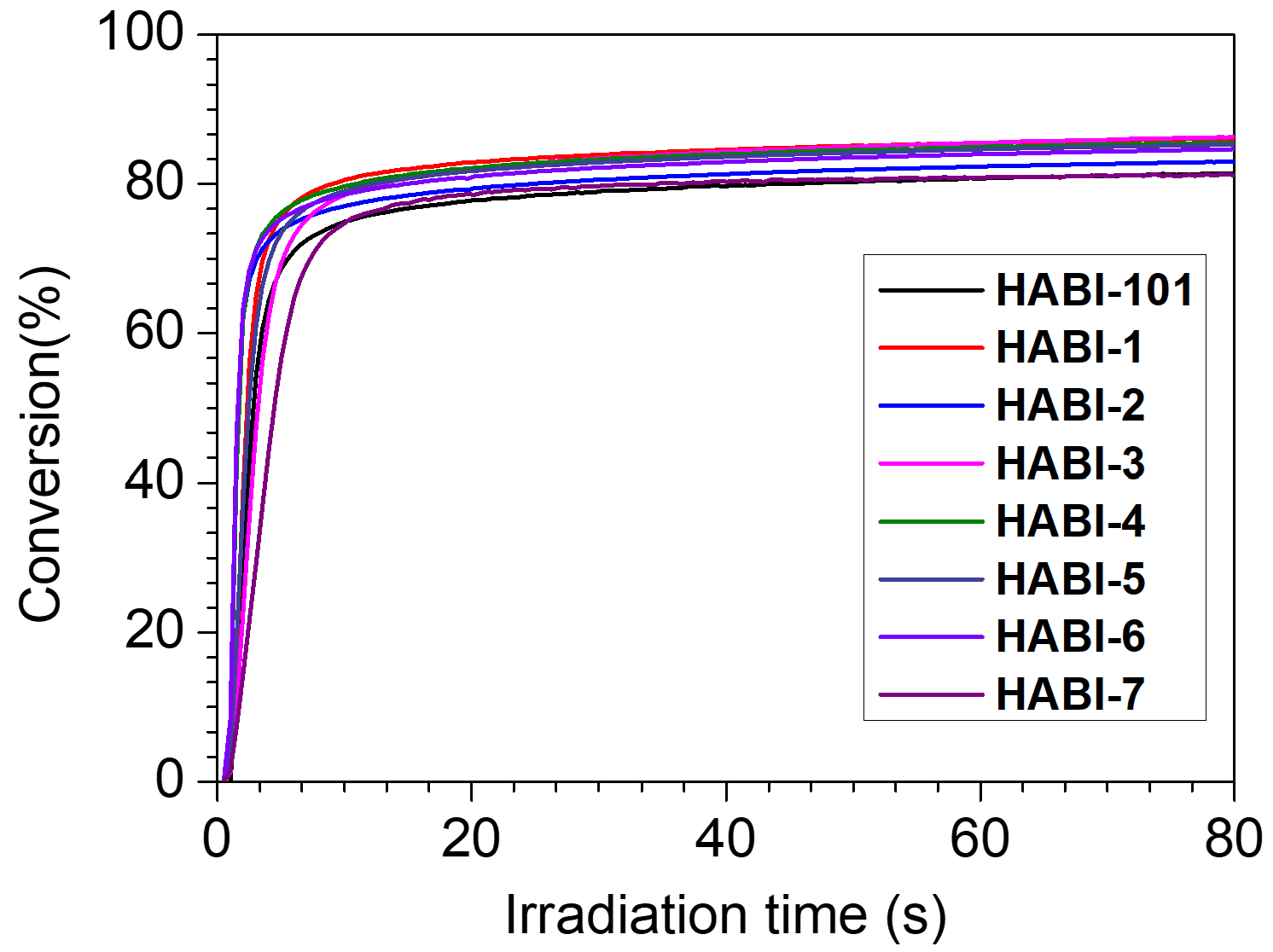


**Figure S28**. Double bond conversion of **TPGDA** with **HABI**s (1 mol% to **TPGDA**) in the presence of the **NPG** (2 mol% to **TPGDA**) applying a LED source emitting at 365 nm with an excitation density of 50 mW·cm^-2^.

## Quantum Chemical Calculations


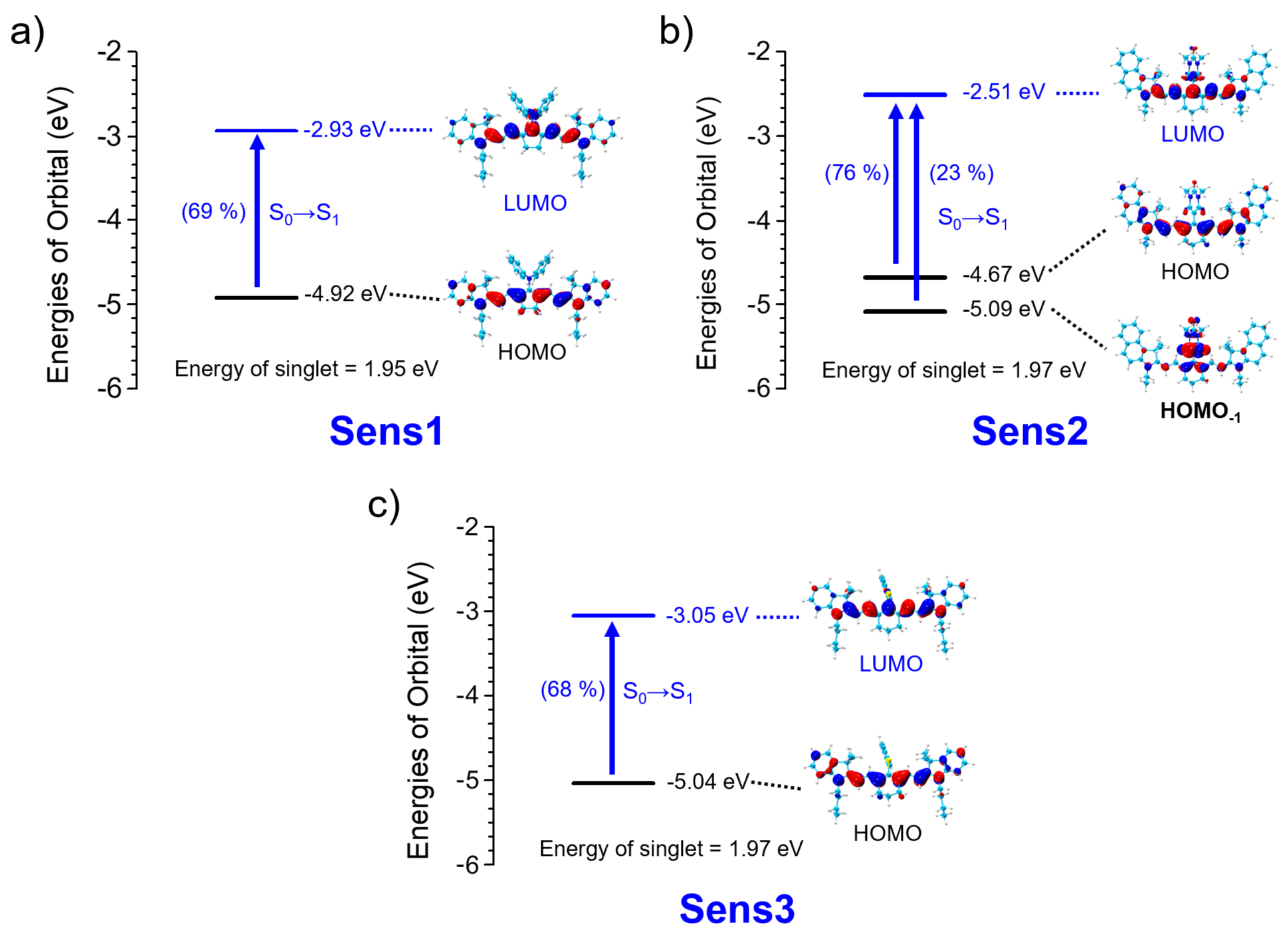


**Figure S29**. Frontier molecular orbits and transition methods of S_0_→S_1_ for (a) **Sens1**, (b) **Sens2** and (c) **Sens3** calculated the TD/B3LYP/6-31G(d) (isovalue = 0.03).


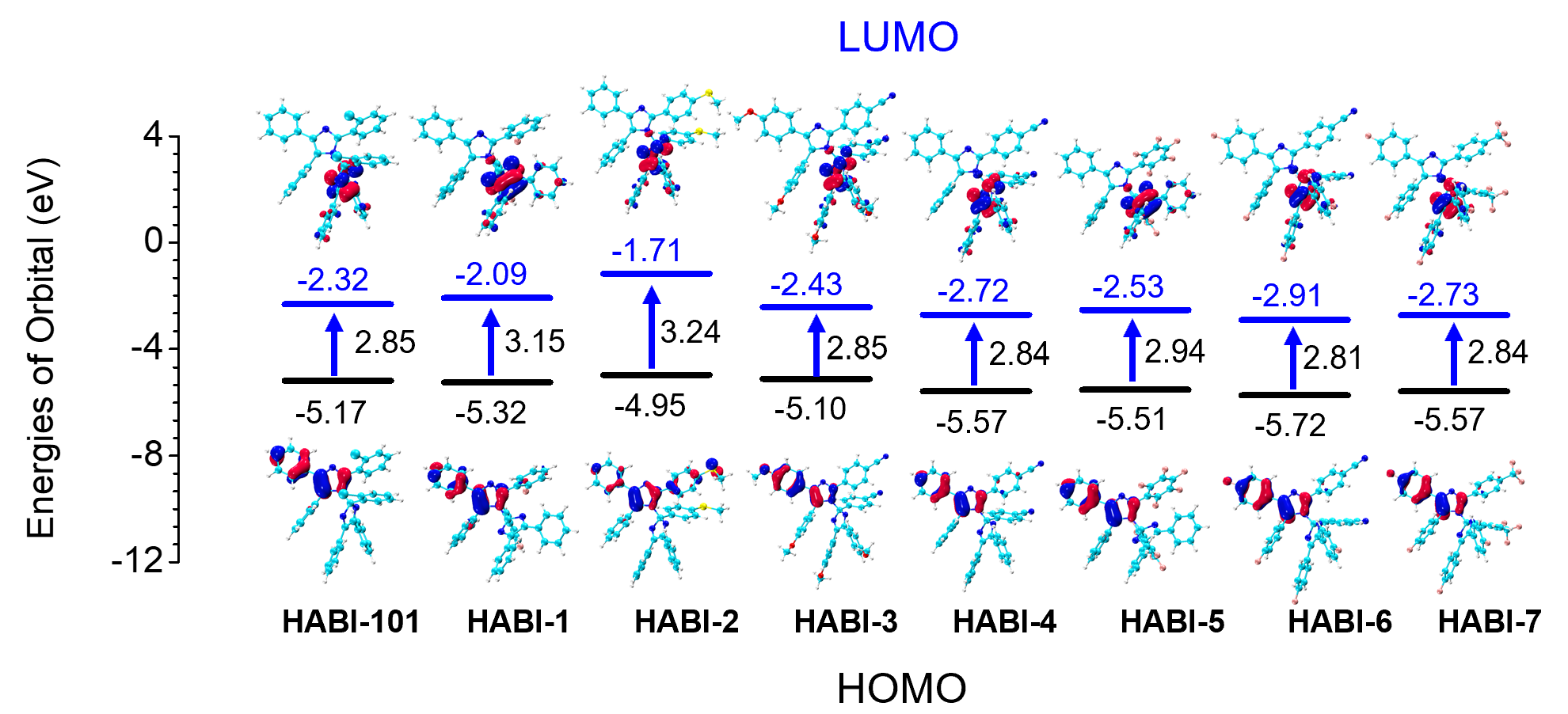


**Figure S30**. Frontier molecular orbits and energies of **HABI**s calculated the B3LYP/6-31G(d) (isovalue = 0.03).


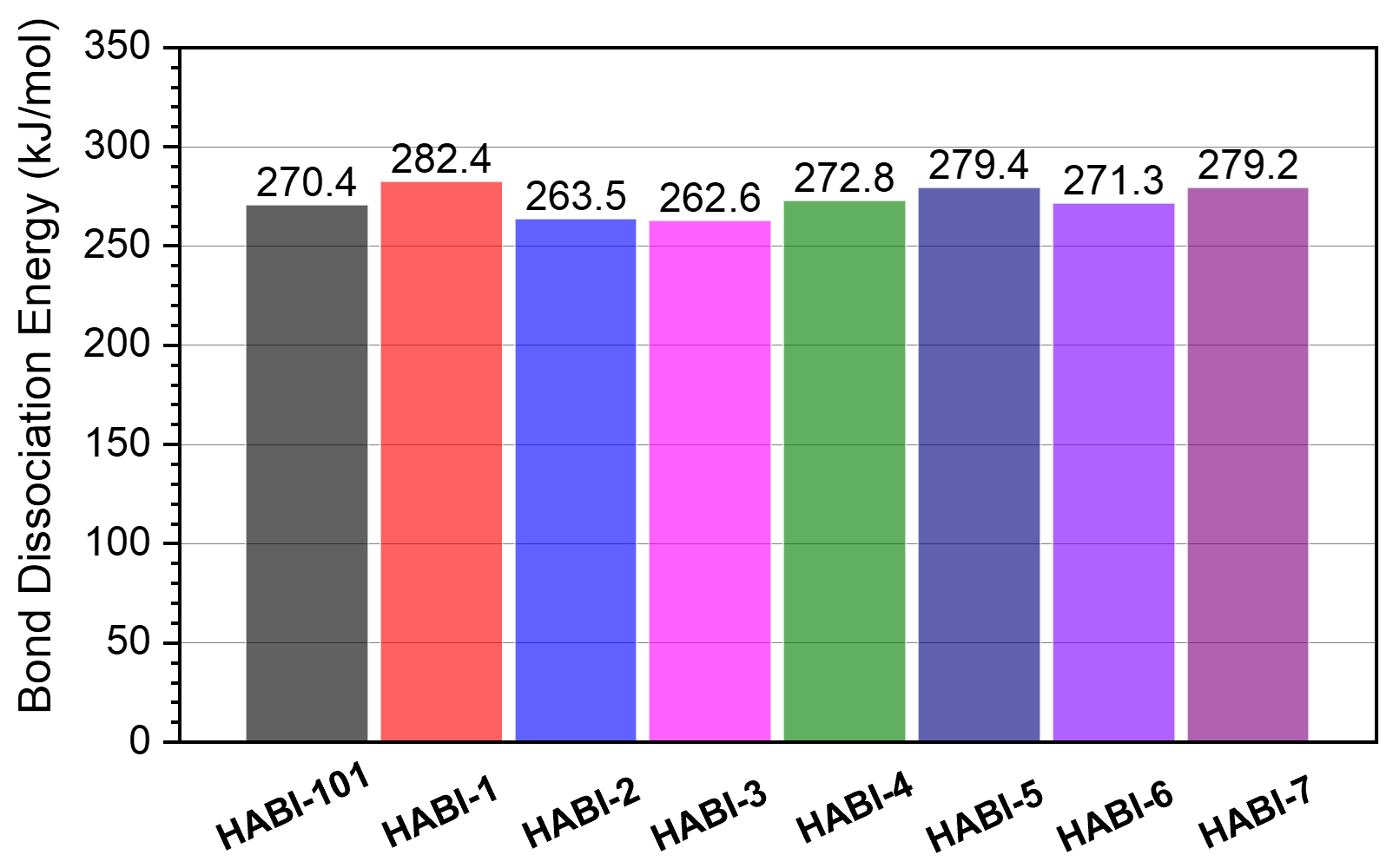


**Figure S31**. Bond dissociation energy (BDE) of **HABI**s cleavage site calculated by the B3LYP/6-31G(d) and UB3LYP/6-31G(d).


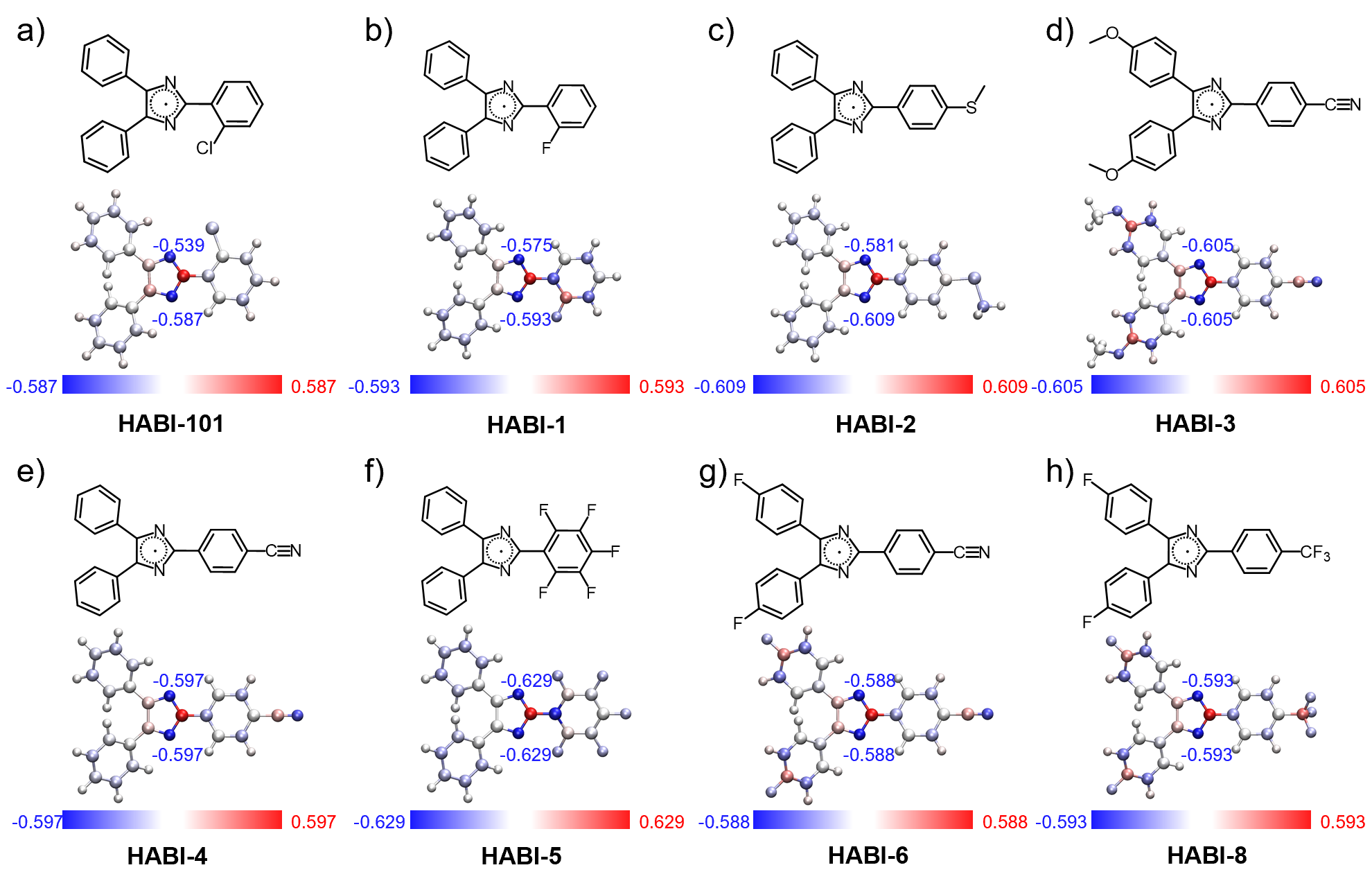


**Figure S32**. Charge distribution of **L•** generated by **HABI**s calculated by the UB3LYP/6-31G(d).


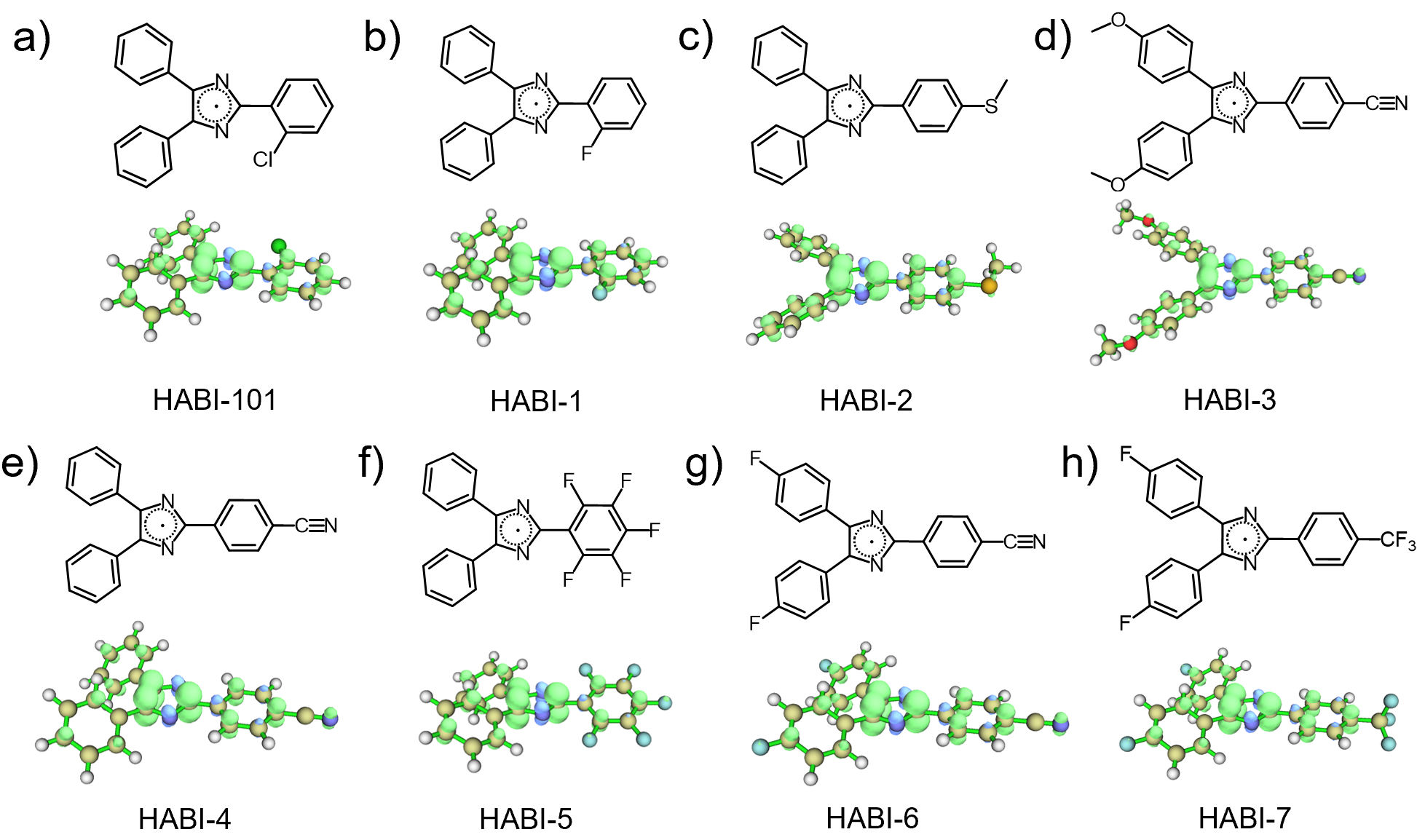


**Figure S33**. Spin density of **L•** generated by **HABI**s calculated by the UB3LYP/6-31G(d).

## Photochemical Properties of Sens/HABI/NPG Systems


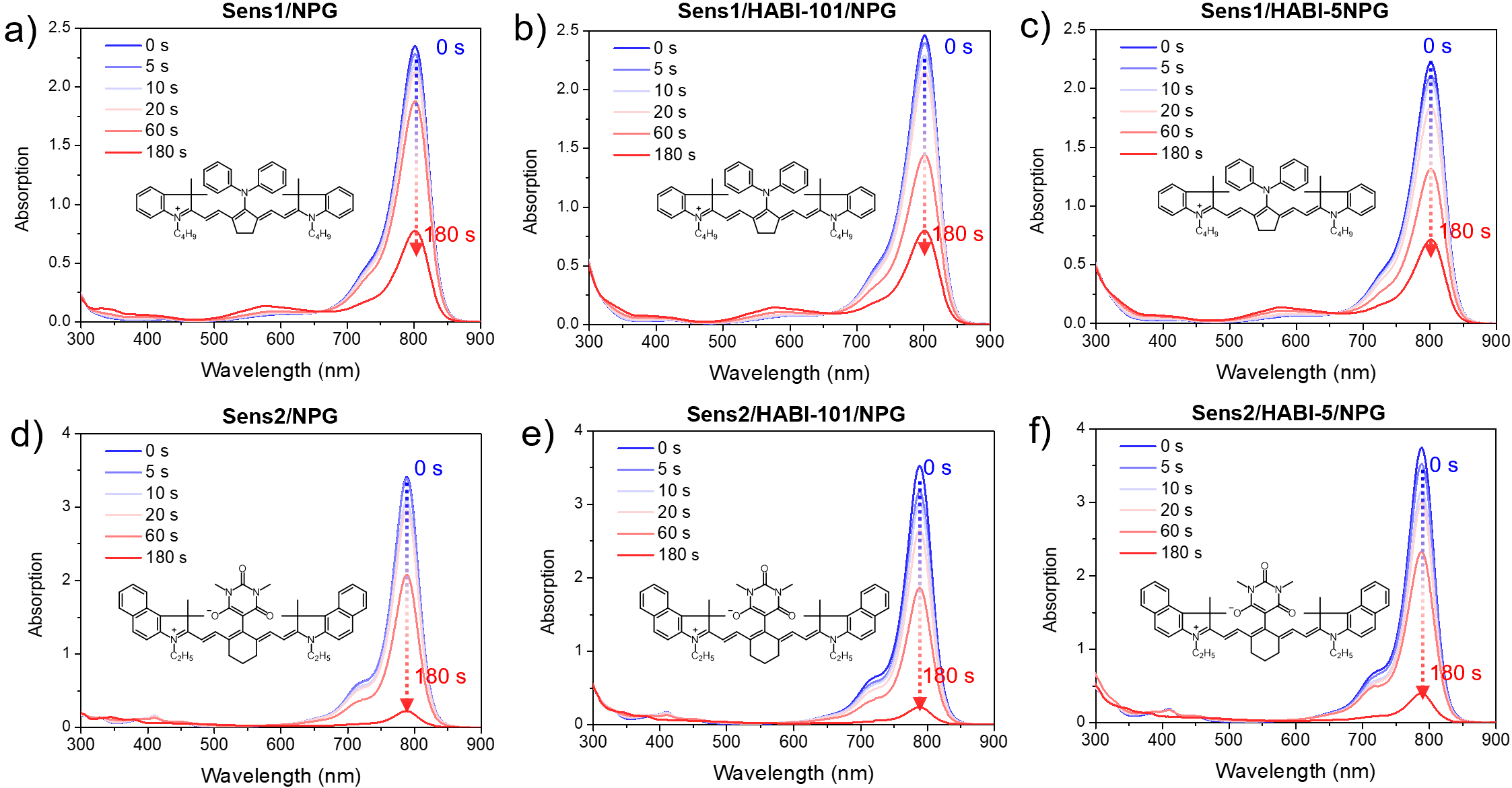


**Figure S34**. Photolysis curves of **Sens/NPG** and **Sens/HABI**s**/NPG** systems in anhydrous dichloromethane upon irradiation at 808 nm laser having an intensity of 714 mW cm^-2^ (concentration = 1.0 × 10^−5^ mol L^−1^ for **Sens**, 2 × 10^-5^ mol L^-1^ for **HABI**s and 4 × 10^-5^ mol L^-1^ for **NPG**); (a) **Sens1/NPG** system, (b) **Sens1/HABI-101/NPG** system, (c) **Sens1/HABI-5/NPG** system, (d) **Sens2/NPG** system, (e) **Sens2/HABI-101/NPG** system, (f) **Sens2/HABI-5/NPG** system.


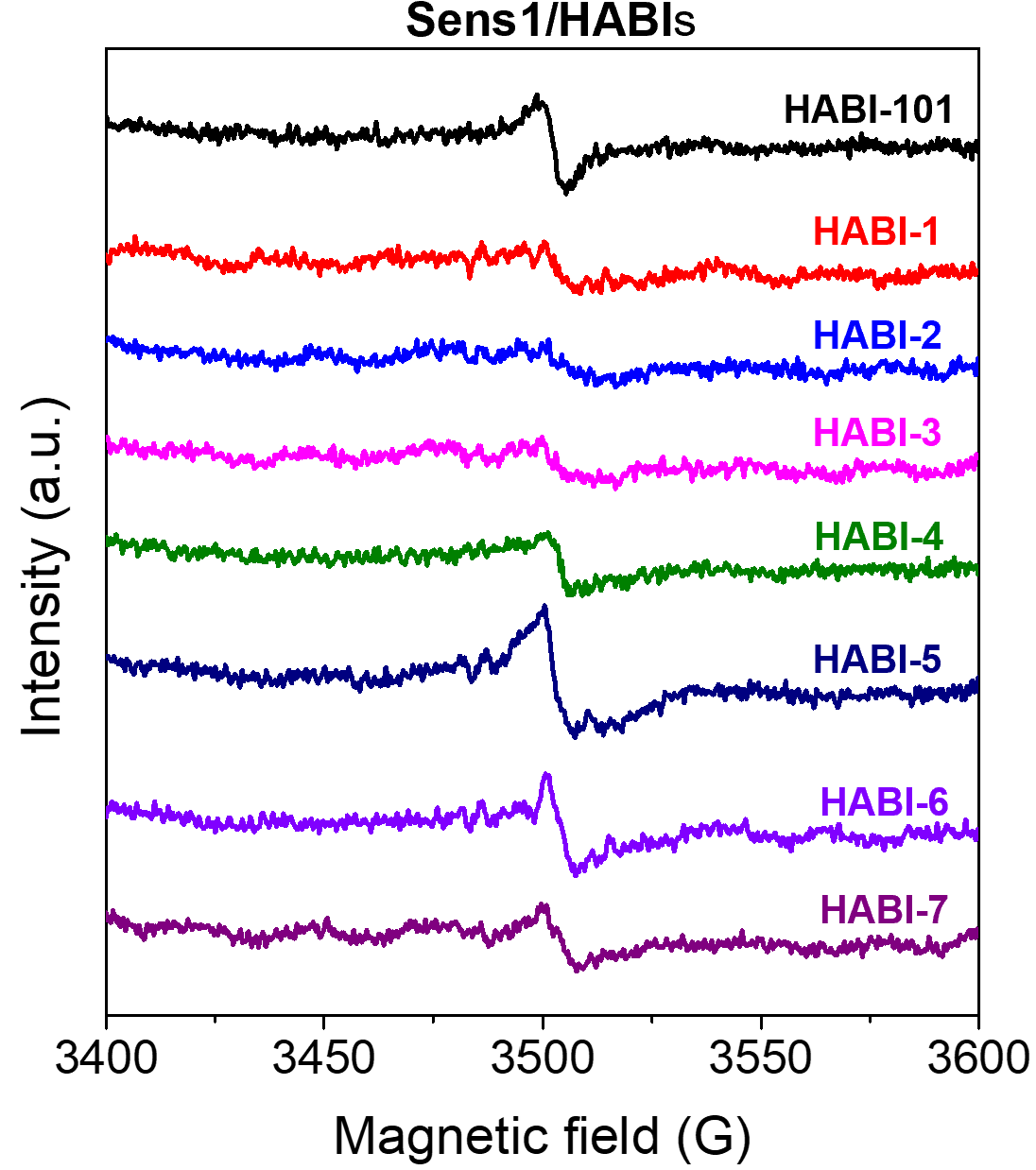


**Figure S35**. ESR generated by dichloromethane solution of **Sens1/HABI**s systems under irradiation at 808 nm laser source with an exposure density of 714 mW·cm^-2^, (concentration = 1.0 × 10^−3^ M for **Sens1**, 5.0 × 10^−3^ M for **HABI**s).


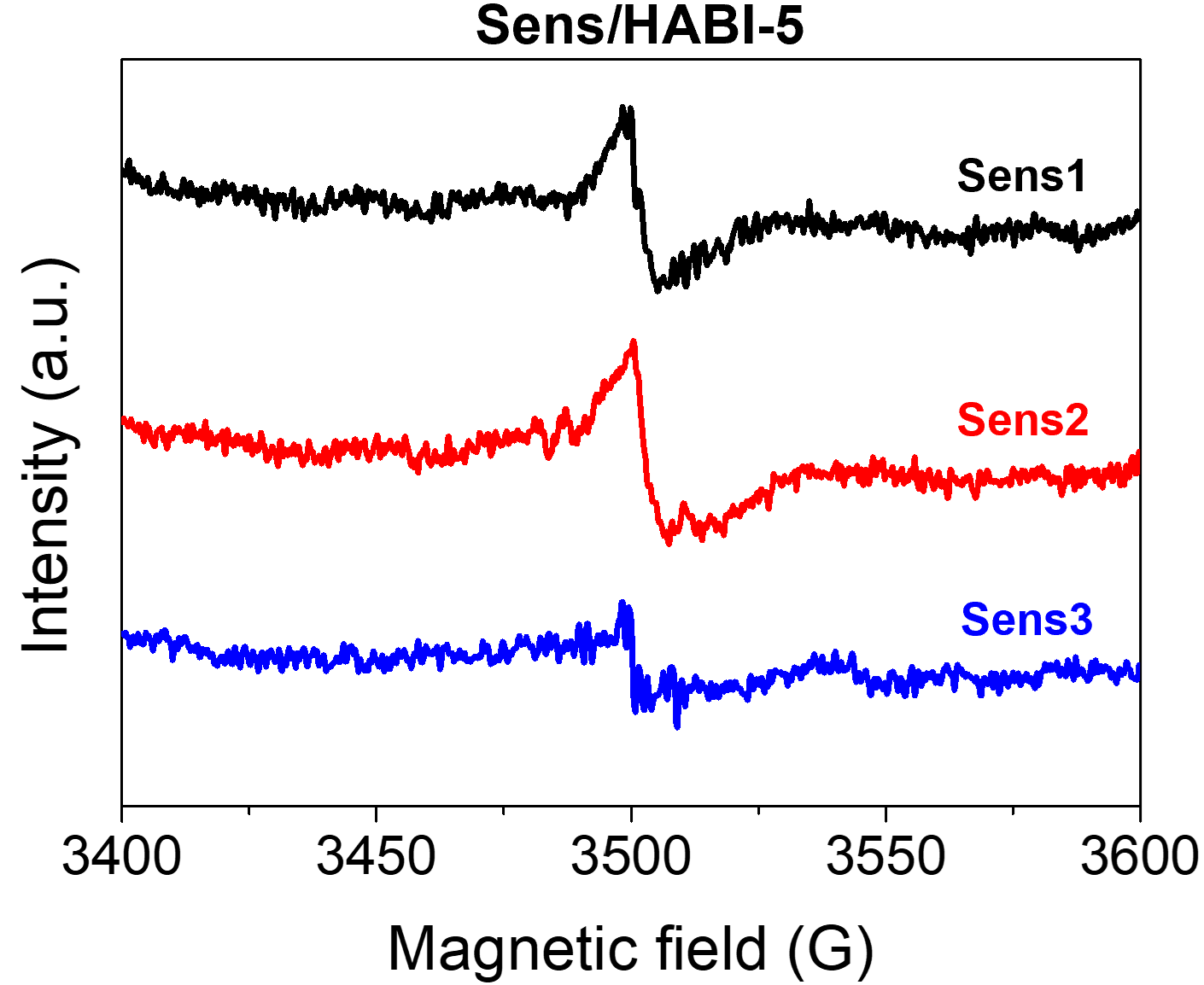


**Figure S36**. ESR generated by dichloromethane solution of **Sens/HABI-5** systems under irradiation at 808 nm laser source with an exposure density of 714 mW·cm^-2^, (concentration = 1.0 × 10^−3^ M for **Sens**, 5.0 × 10^−3^ M for **HABI-5**).


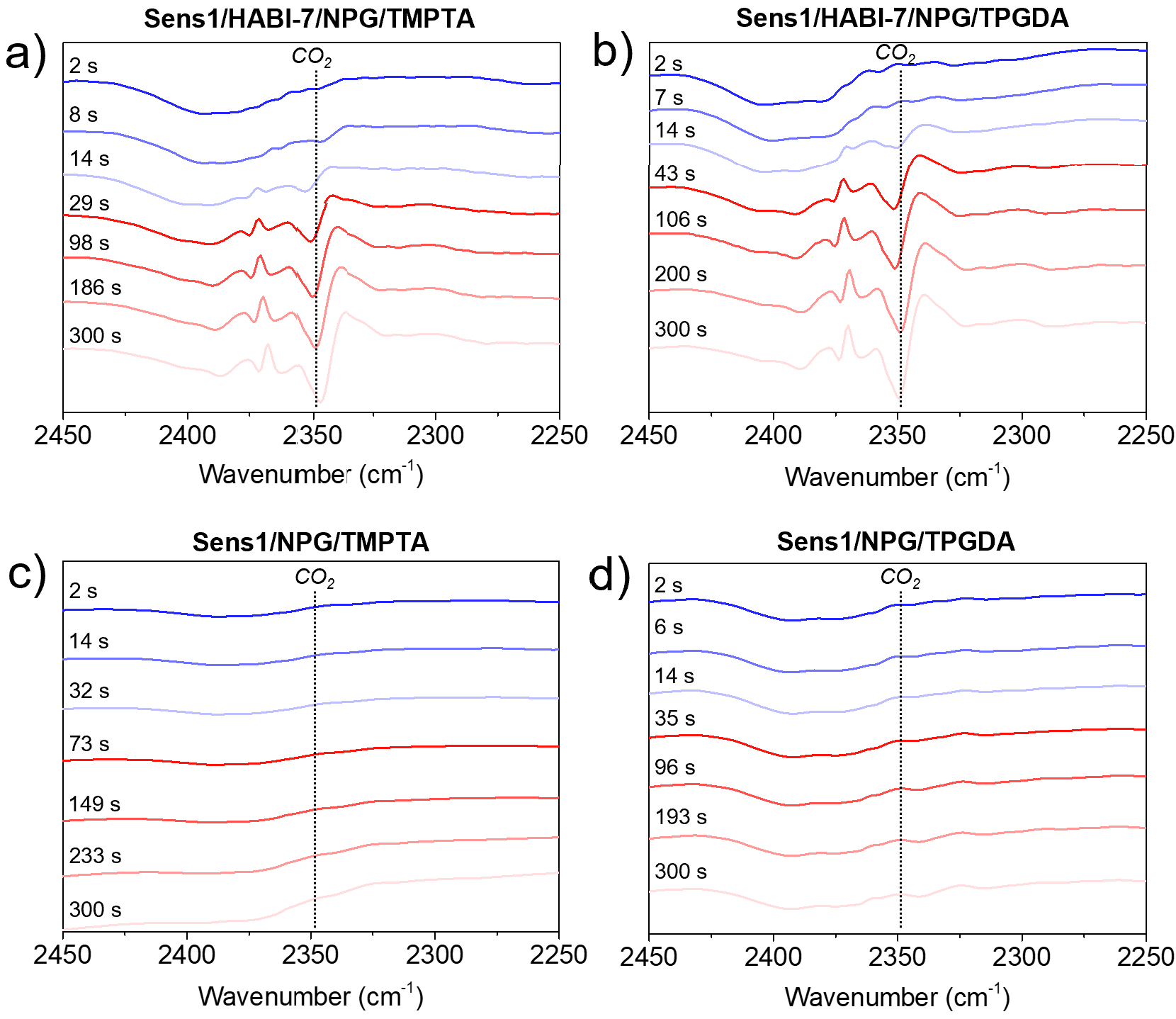


**Figure S37**. Variation of the IR peak signal intensity of CO_2_ during the photopolymerization process by testing the **Sens1**/**NPG**/**monomer**s (0.2 mol% for **Sens1** and 2 mol% for **NPG** to **monomer**s) and **Sens1**/**HABI-7**/**NPG**/**monomer**s (0.2 mol% for **Sens1,** 1 mol% for **HABI-7** and 2 mol% for **NPG** to **monomer**s) systems upon irradiation at 808 nm laser (714 mW cm^−2^).

## Effect of Heat on Photopolymerization Reaction


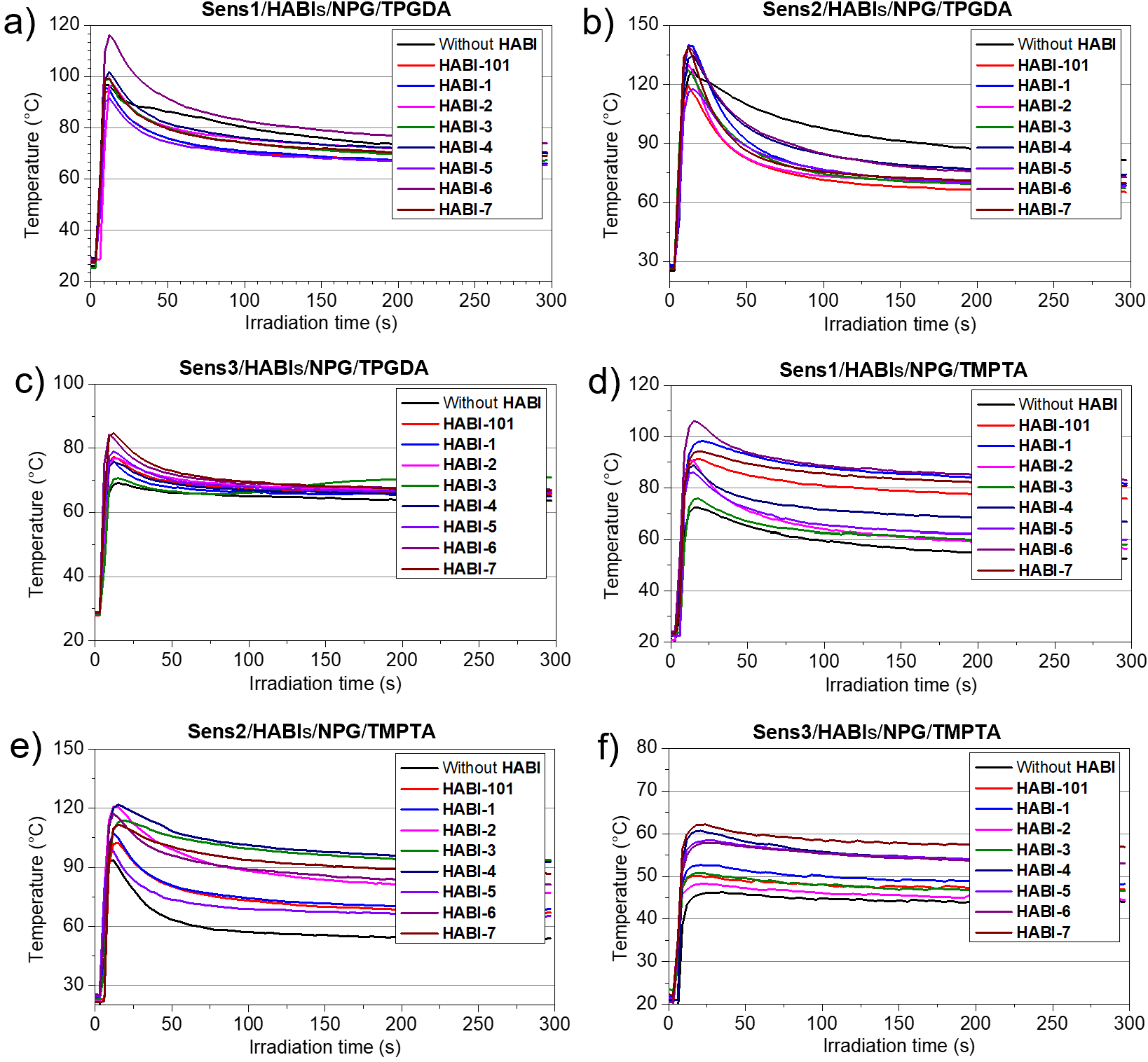


**Figure S38**. The temperature generated by **Sens** (0.2 mol% to **monomer**s) in the samples (thickness: about 30 μm) comprising **HABI**s (1 mol% to **monomer**s), **NPG** (2 mol% to **monomer**s) and **monomer**s. The samples were irradiated with the laser device at 808 nm having an intensity of 714 mW cm^-2^; (a) **Sens1/HABI**s**/NPG/TPGDA** systems, (b) **Sens2/HABI**s**/NPG/TPGDA** systems, (c) **Sens3/HABI**s**/NPG/TPGDA** systems, (d) **Sens1/HABI**s**/NPG/TMPTA** systems, (e) **Sens2/HABI**s**/NPG/TMPTA** systems, (f) **Sens3/HABI**s**/NPG/TMPTA** systems.

**Table S4** Temperature maximum (T_m_) during photopolymerization of **Sens**/**HABI**s/**NPG**/**monomer**s systems.

| **HABI**s | Temperature maximum (℃) | | | | | |
| --- | --- | --- | --- | --- | --- | --- |
|  | **Sens**/**HABI**s/**NPG**/**TPGDA** | | | **Sens**/**HABI**s/**NPG**/**TMPTA** | | |
|  | **Sens1** | **Sens2** | **Sens1** | **Sens2** | **Sens1** | **Sens2** |
| Without **HABI**s | 96.6 | 127.7 | 69.1 | 72.3 | 93.6 | 46.3 |
| **HABI-101** | 94.4 | 119.5 | 77.3 | 91.3 | 102.5 | 50.1 |
| **HABI-1** | 95.8 | 140.0 | 76.2 | 98.4 | 107.3 | 52.7 |
| **HABI-2** | 96.4 | 130.3 | 76.8 | 90.9 | 120.7 | 48.3 |
| **HABI-3** | 99.2 | 127.3 | 70.6 | 75.9 | 113.8 | 50.8 |
| **HABI-4** | 101.9 | 134.8 | 75.7 | 88.9 | 121.8 | 60.7 |
| **HABI-5** | 91.5 | 117.8 | 79.0 | 86.0 | 99.4 | 58.4 |
| **HABI-6** | 116.4 | 139.5 | 84.2 | 106.3 | 117.1 | 57.8 |
| **HABI-7** | 99.4 | 139.1 | 84.7 | 94.3 | 111.8 | 62.0 |


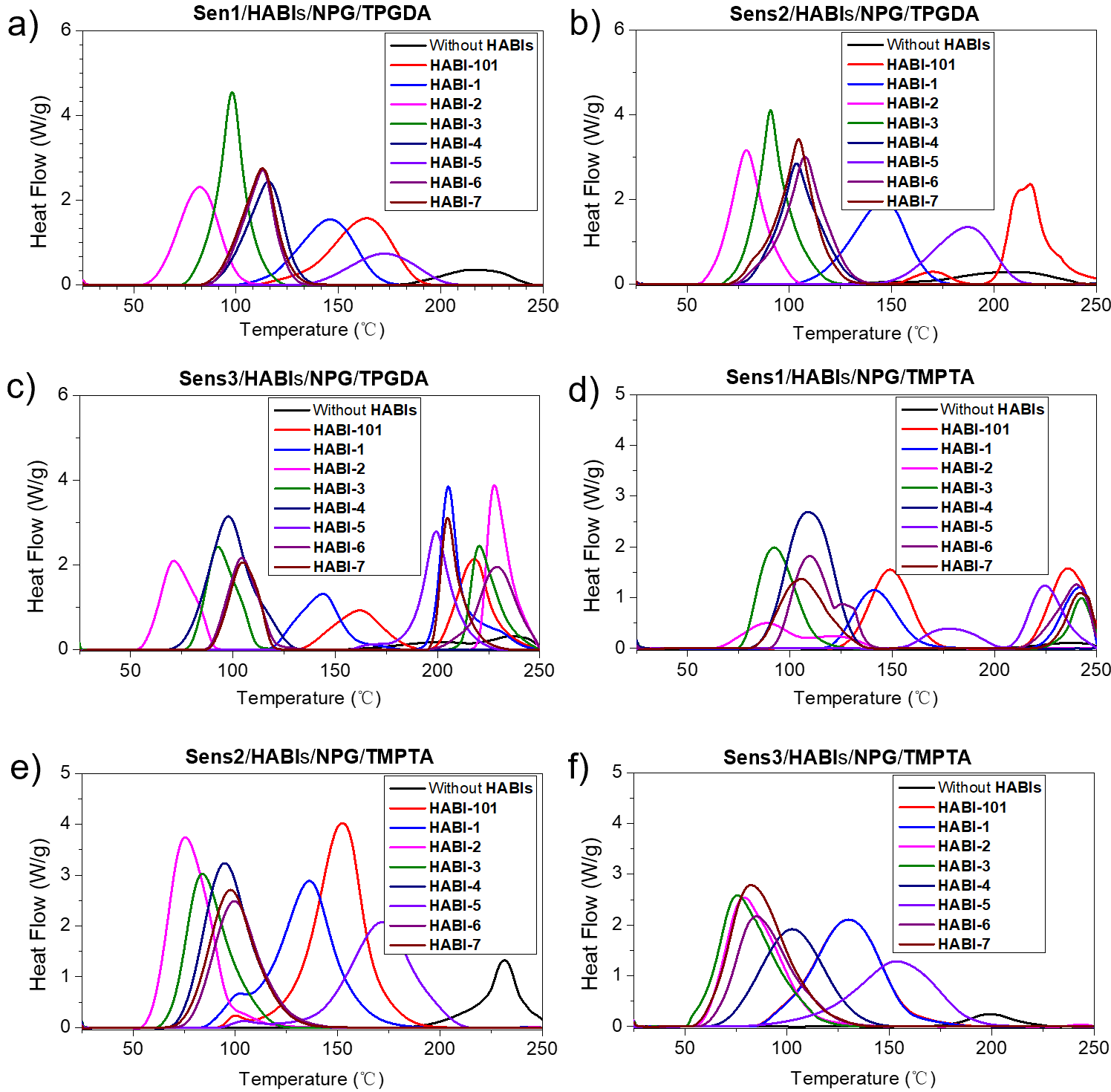


**Figure S39**. DSC of systems comprising **Sens** (0.2 mol% to **monomer**s), **HABI**s (1 mol% to **monomer**s), **NPG** (2 mol% to **monomer**s) and **monomer**s upon heating using a rate of 10 ℃ min^−1^; (a) (a) **Sens1/HABI**s**/NPG/TPGDA** systems, (b) **Sens2/HABI**s**/NPG/TPGDA** systems, (c) **Sens3/HABI**s**/NPG/TPGDA** systems, (d) **Sens1/HABI**s**/NPG/TMPTA** systems, (e) **Sens2/HABI**s**/NPG/TMPTA** systems, (f) **Sens3/HABI**s**/NPG/TMPTA** systems.

**Table S5** The onset of thermal radical polymerization of **Sens/HABI**s**/NPG/monomer**s systems detected by DSC.

| **HABI**s | Onset of thermal radical polymerization (℃) | | | | | |
| --- | --- | --- | --- | --- | --- | --- |
|  | **Sens**/**HABI**s/**NPG**/**TPGDA** | | | **Sens**/**HABI**s/**NPG**/**TMPTA** | | |
|  | **Sens1** | **Sens2** | **Sens1** | **Sens2** | **Sens1** | **Sens2** |
| Without **HABI**s | 185.7 | 154.0 | 167.4 | 215.5 | 189.8 | 173.3 |
| **HABI-101** | 114.2 | 154.7 | 135.0 | 124.2 | 93.5 | 85.7 |
| **HABI-1** | 105.5 | 105.7 | 118.8 | 118.7 | 85.8 | 90.1 |
| **HABI-2** | 56.7 | 57.2 | 54.5 | 66.2 | 55.2 | 56.3 |
| **HABI-3** | 75.2 | 68.5 | 77.8 | 75.3 | 63.0 | 51.2 |
| **HABI-4** | 87.5 | 77.7 | 71.2 | 82.3 | 69.3 | 64.5 |
| **HABI-5** | 137.2 | 148.3 | 160.0 | 155.8 | 96.0 | 86.5 |
| **HABI-6** | 88.2 | 74.8 | 87.5 | 91.3 | 74.0 | 59.0 |
| **HABI-7** | 85.5 | 72.3 | 88.5 | 82.3 | 70.7 | 55.0 |

## LC-MS Data of Sens/HABI-5/NPG System


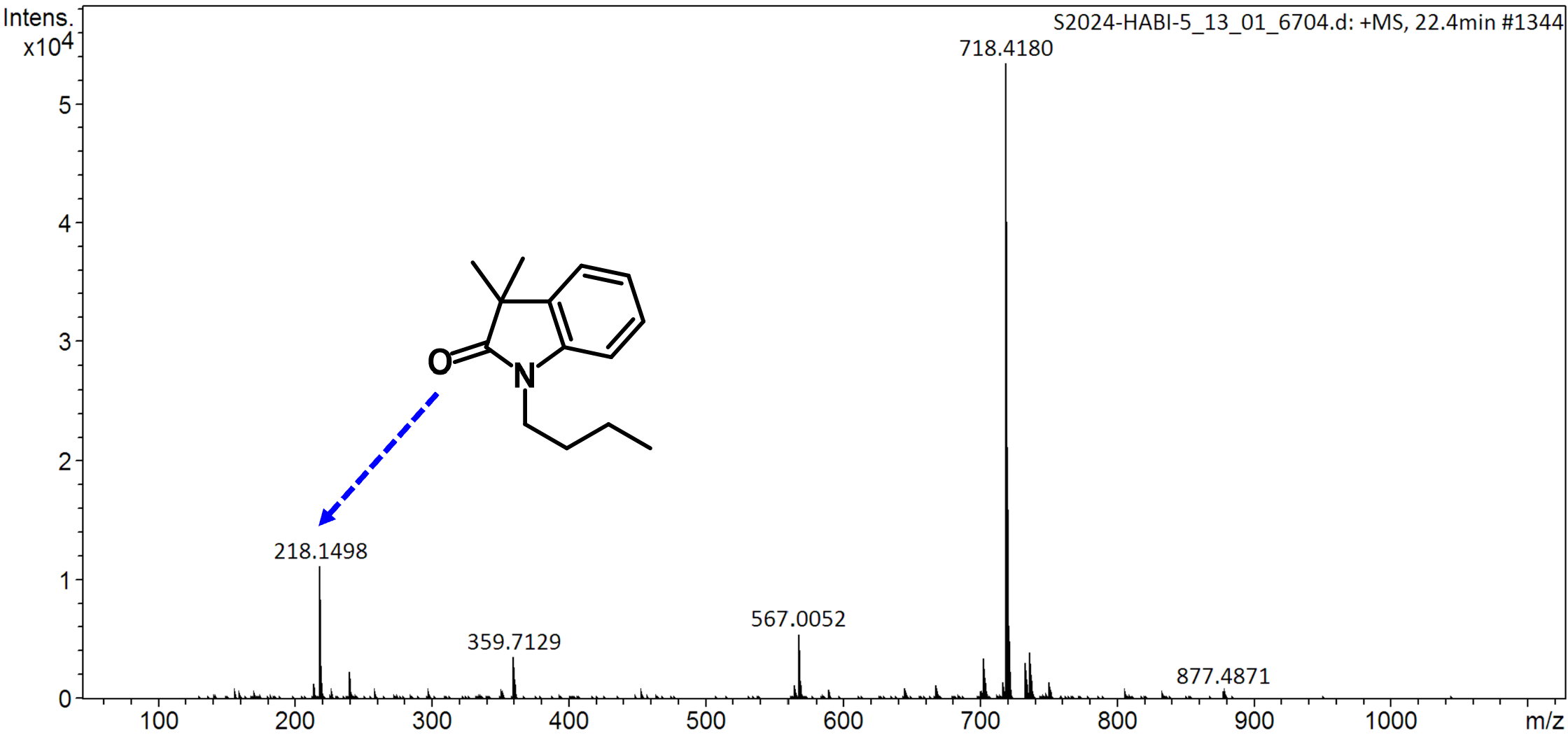


**Figure S40.** LC-MC results of the **Sens1/HABI-5/NPG** system in acetonitrile under 808 nm laser source with an exposure intensity of 714 mW·cm^-2^.


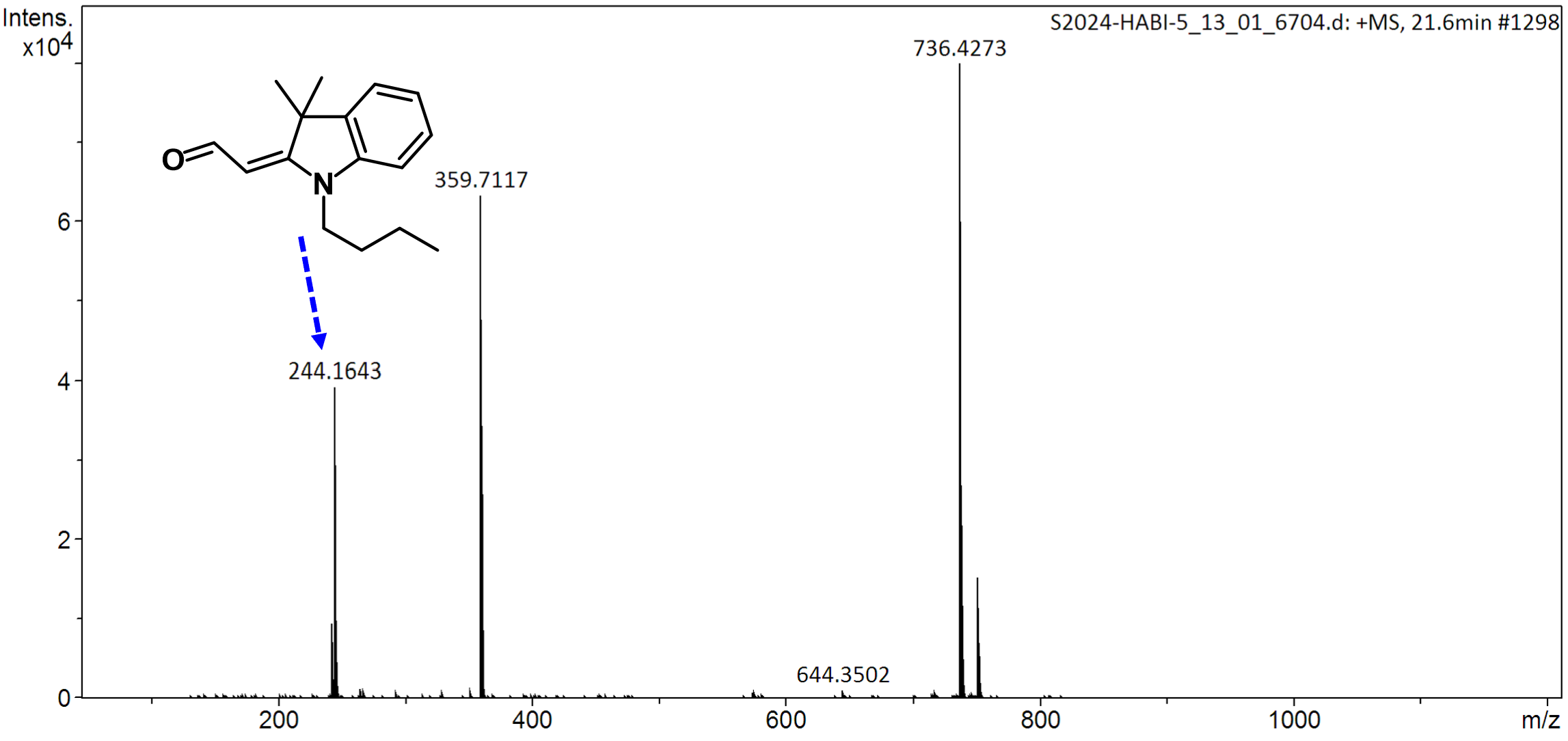


**Figure S41.** LC-MC results of the **Sens1/HABI-5/NPG** system in acetonitrile under 808 nm laser source with an exposure intensity of 714 mW·cm^-2^.


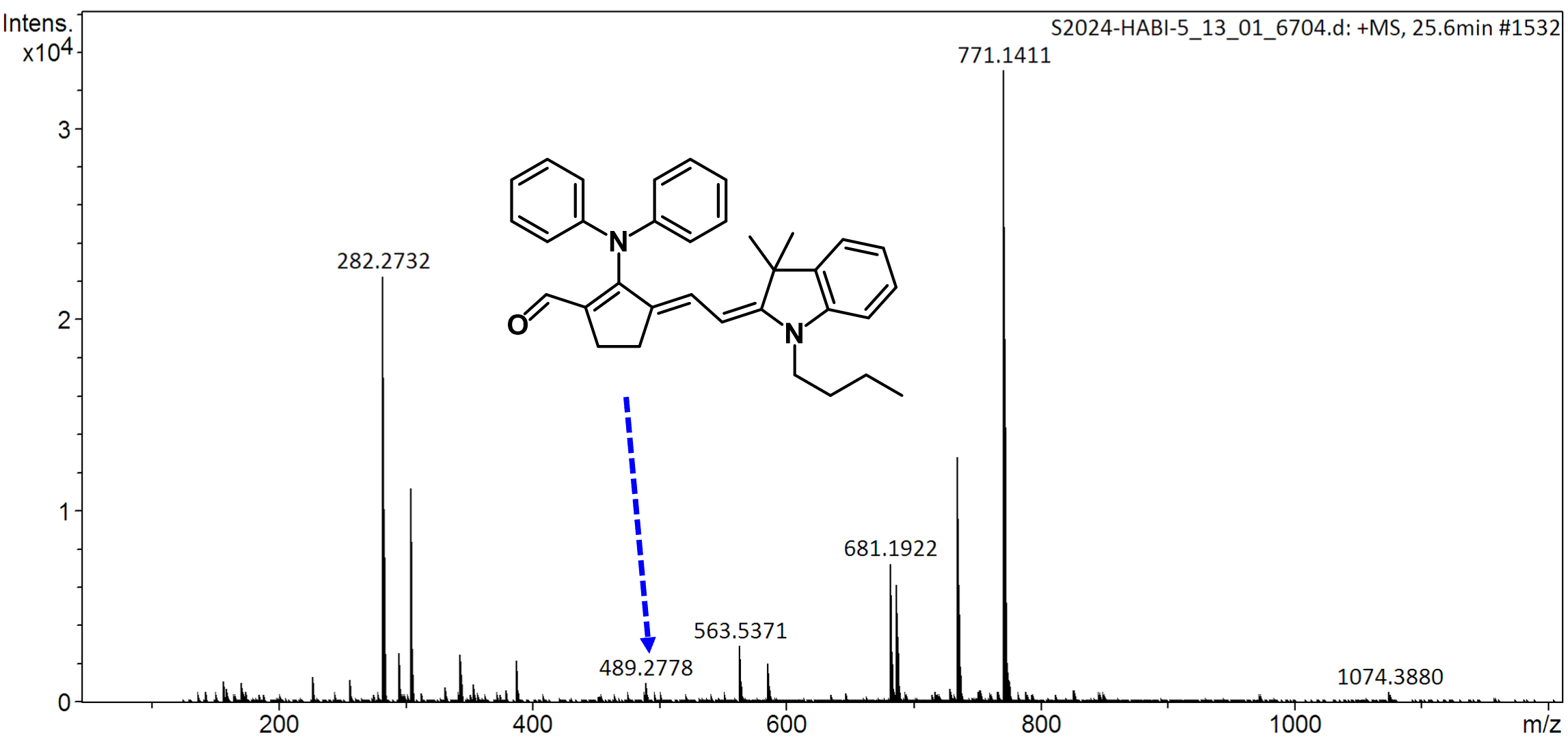


**Figure S42.** LC-MC results of the **Sens1/HABI-5/NPG** system in acetonitrile under 808 nm laser source with an exposure intensity of 714 mW·cm^-2^.


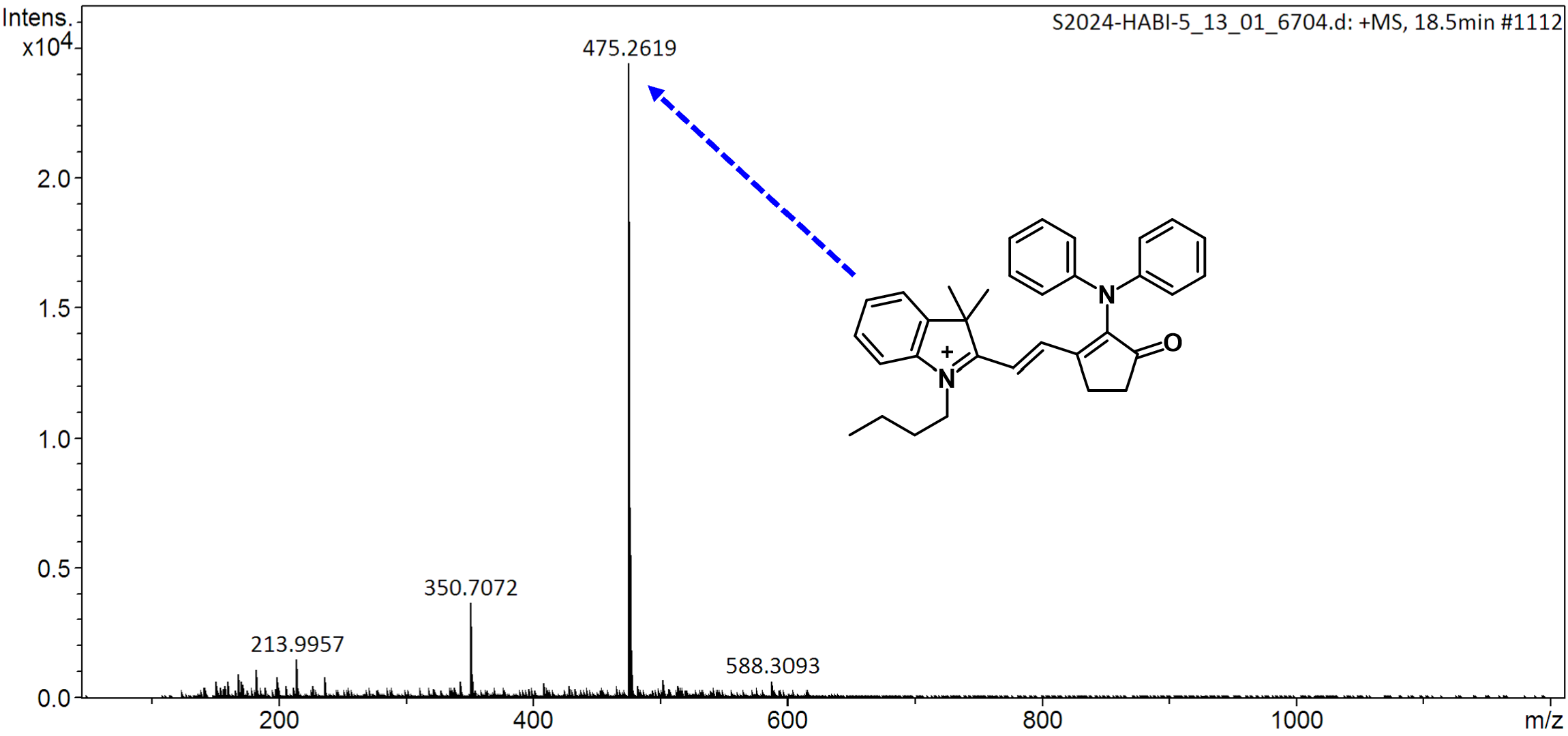


**Figure S43.** LC-MC results of the **Sens1/HABI-5/NPG** system in acetonitrile under 808 nm laser source with an exposure intensity of 714 mW·cm^-2^.


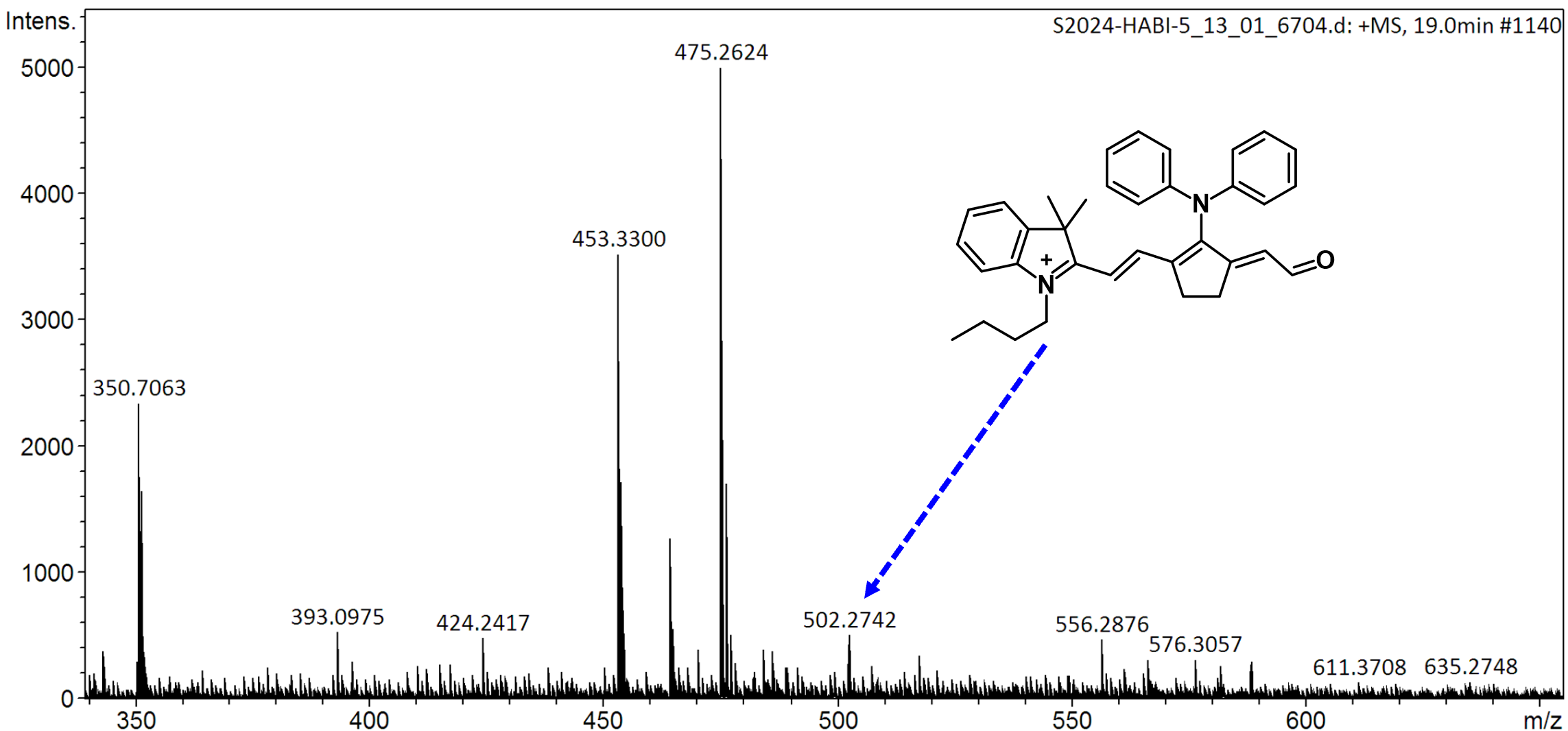


**Figure S44.** LC-MC results of the **Sens1/HABI-5/NPG** system in acetonitrile under 808 nm laser source with an exposure intensity of 714 mW·cm^-2^.


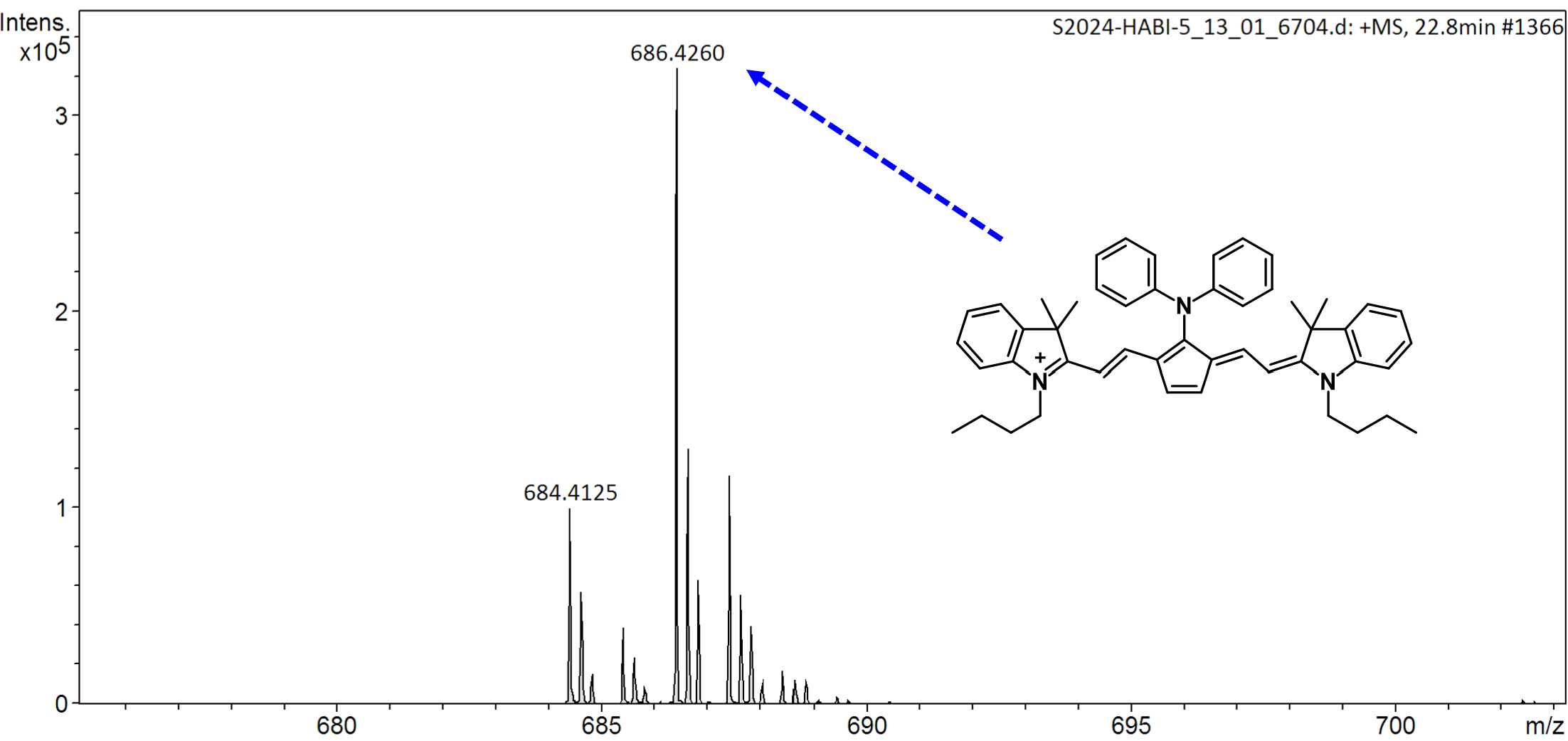


**Figure S45.** LC-MC results of the **Sens1/HABI-5/NPG** system in acetonitrile under 808 nm laser source with an exposure intensity of 714 mW·cm^-2^.


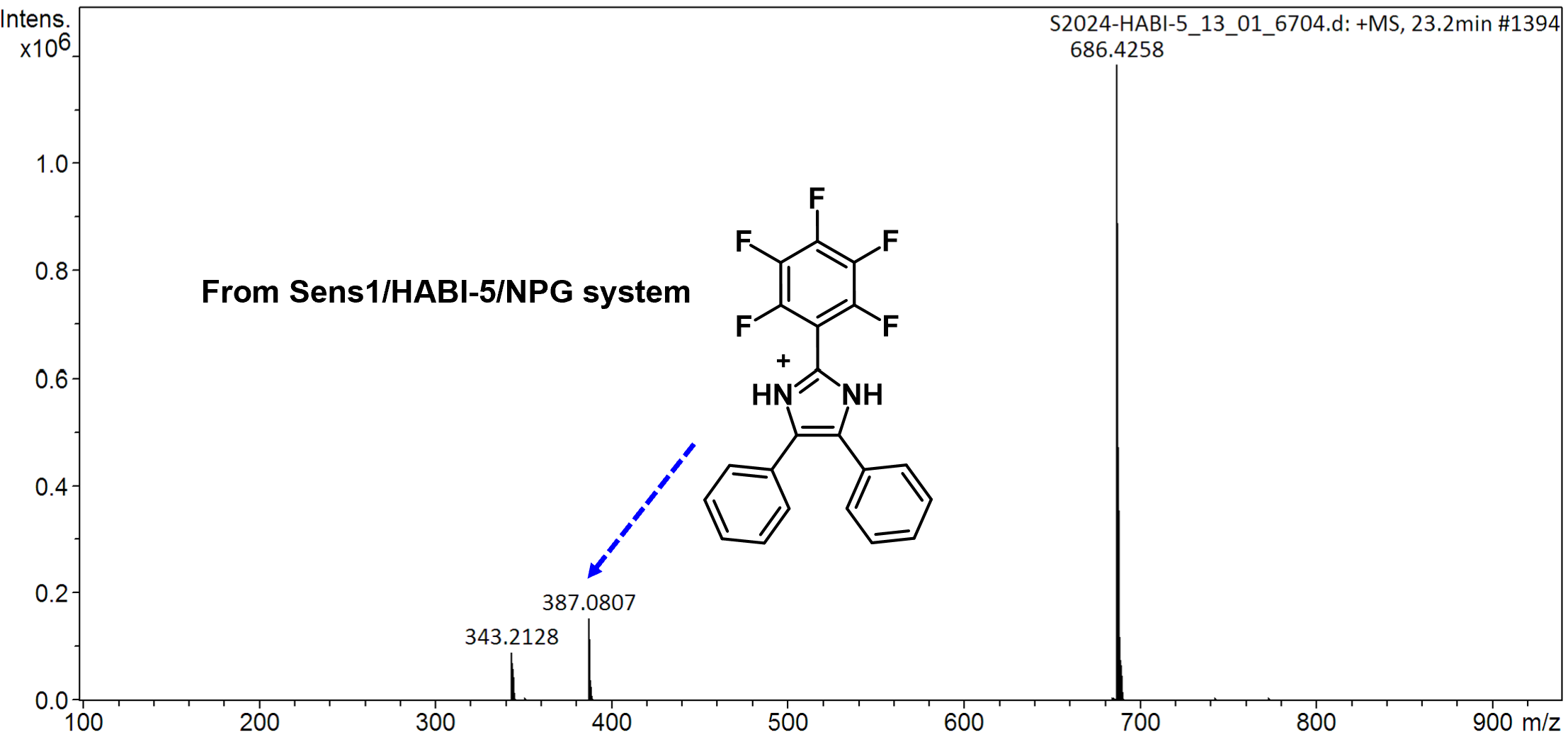


**Figure S46.** LC-MC results of the **Sens1/HABI-5/NPG** system in acetonitrile under 808 nm laser source with an exposure intensity of 714 mW·cm^-2^.


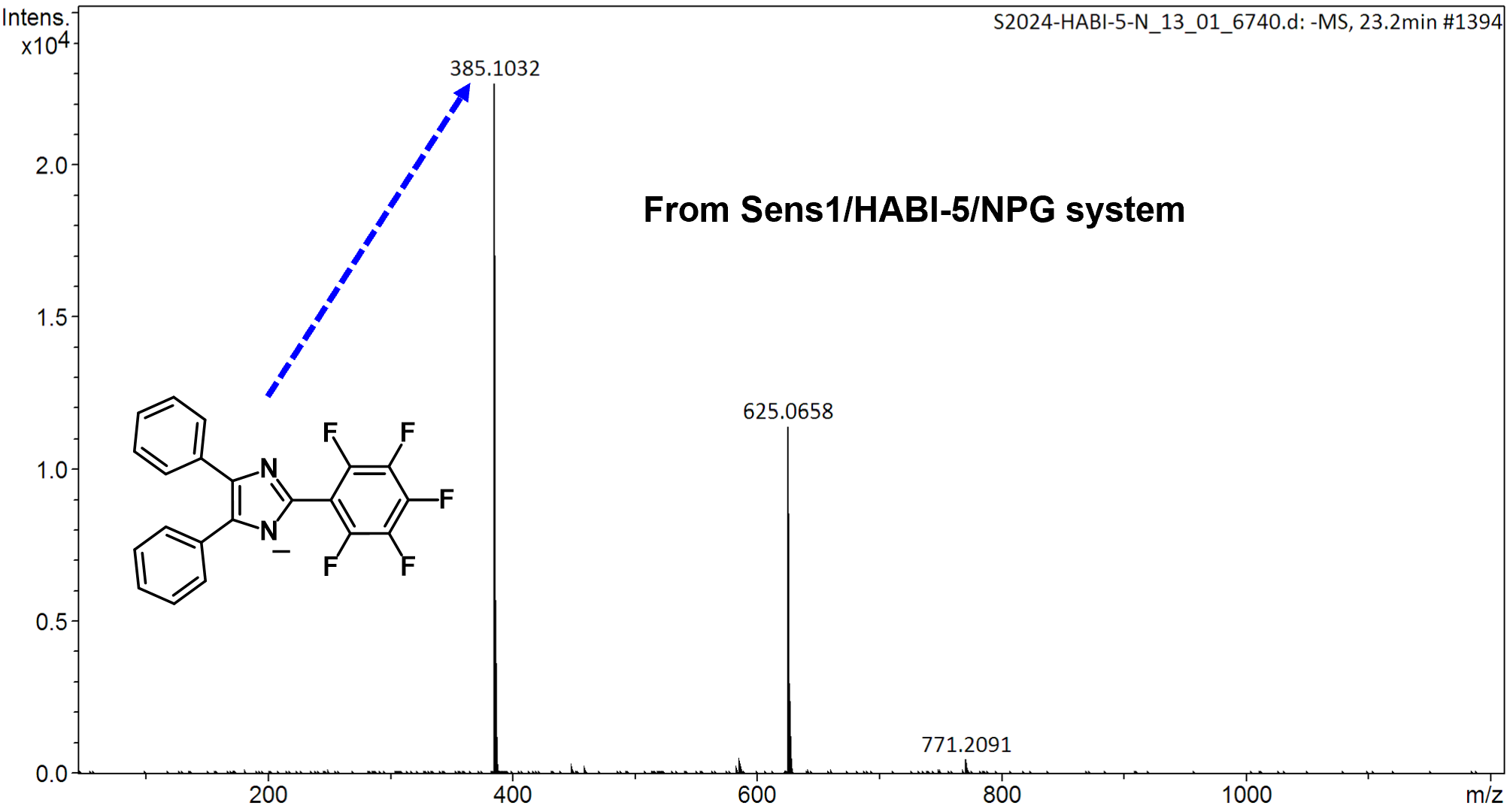


**Figure S47.** LC-MC results of the **Sens1/HABI-5/NPG** system in acetonitrile under 808 nm laser source with an exposure intensity of 714 mW·cm^-2^.


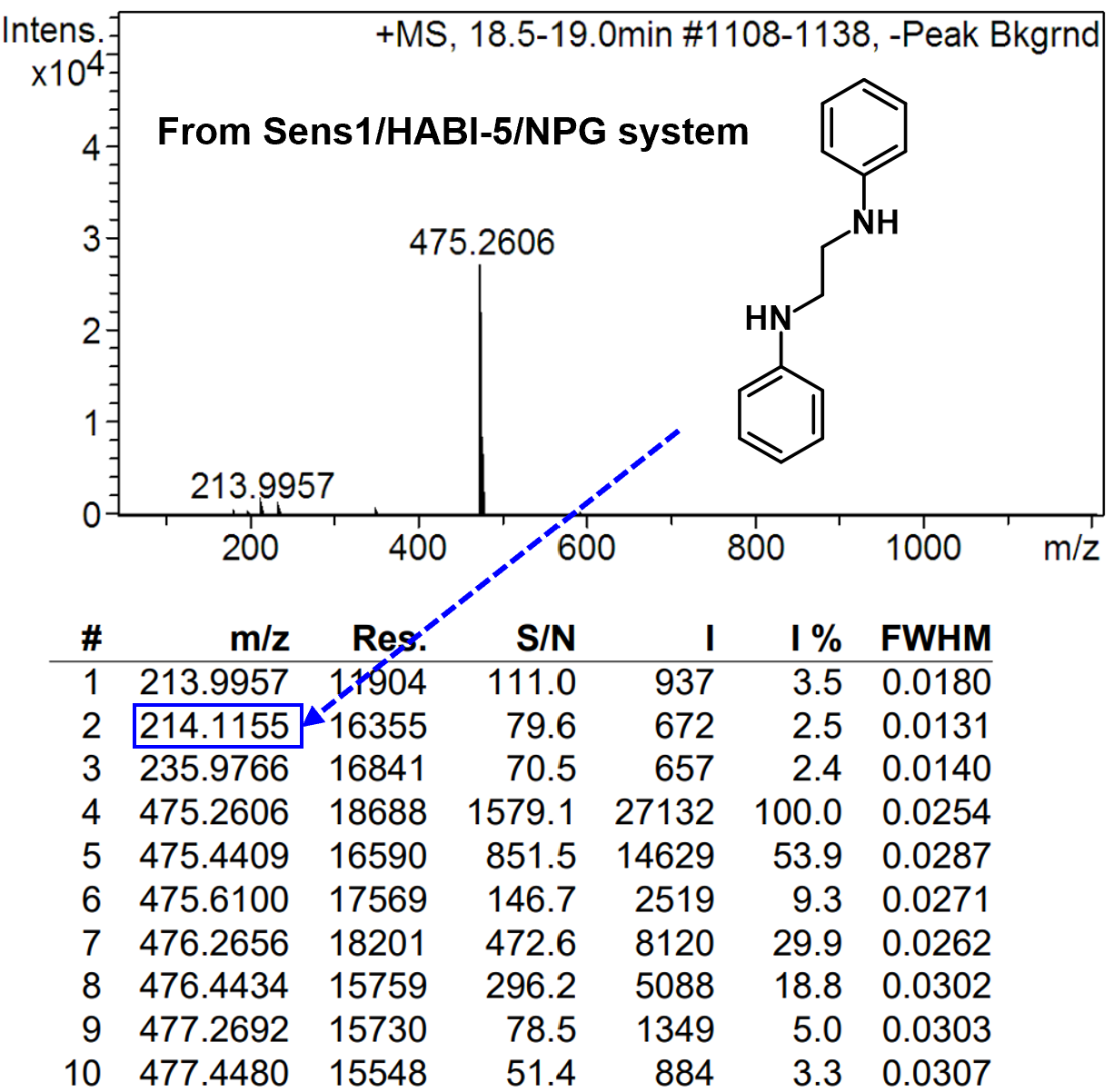


**Figure S48.** LC-MS results of the **Sens1/HABI-5/NPG** system in acetonitrile under 808 nm laser source with an exposure intensity of 714 mW·cm^-2^.


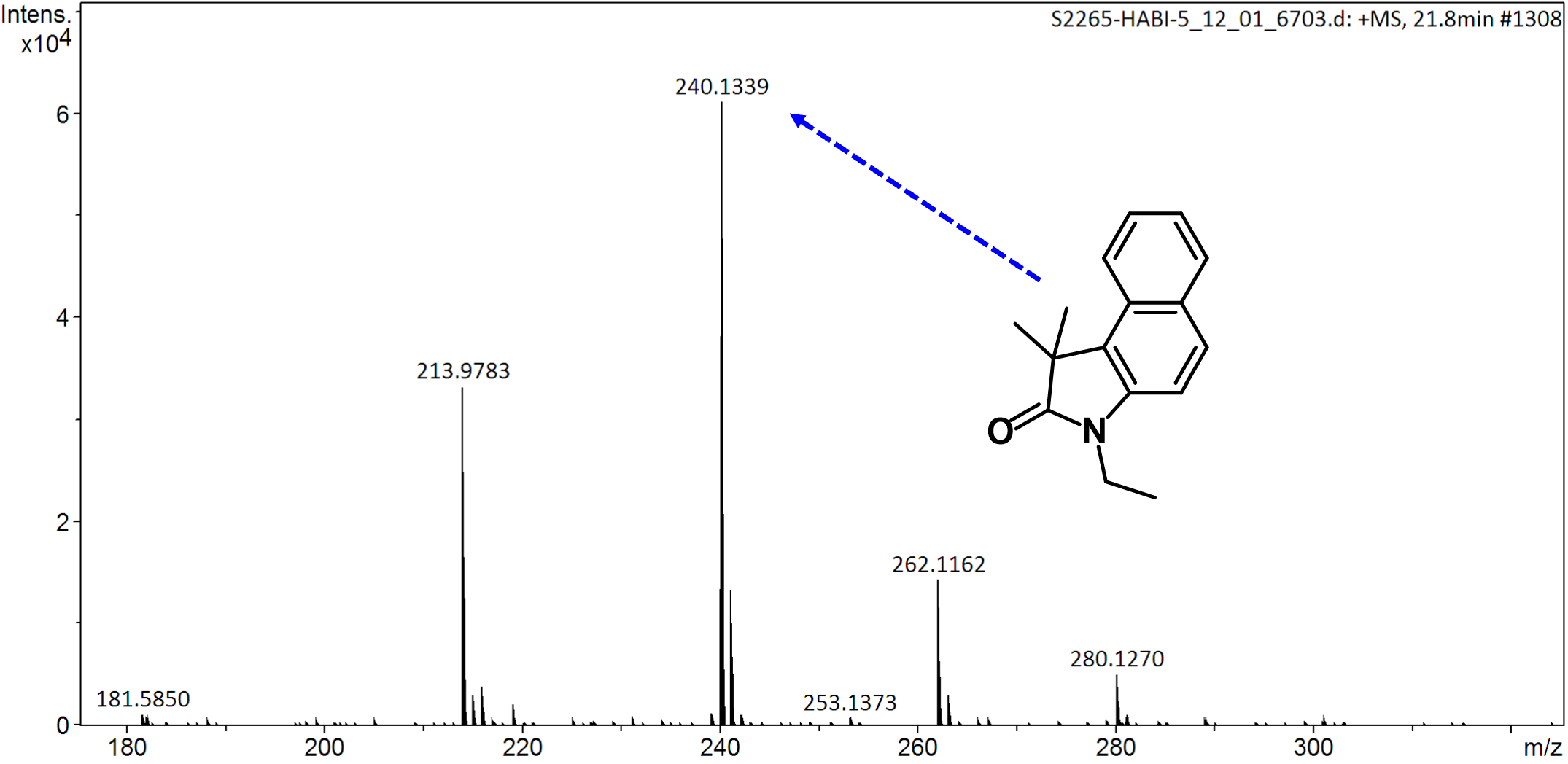


**Figure S49.** LC-MC results of the **Sens2/HABI-5/NPG** system in acetonitrile under 808 nm laser source with an exposure intensity of 714 mW·cm^-2^.


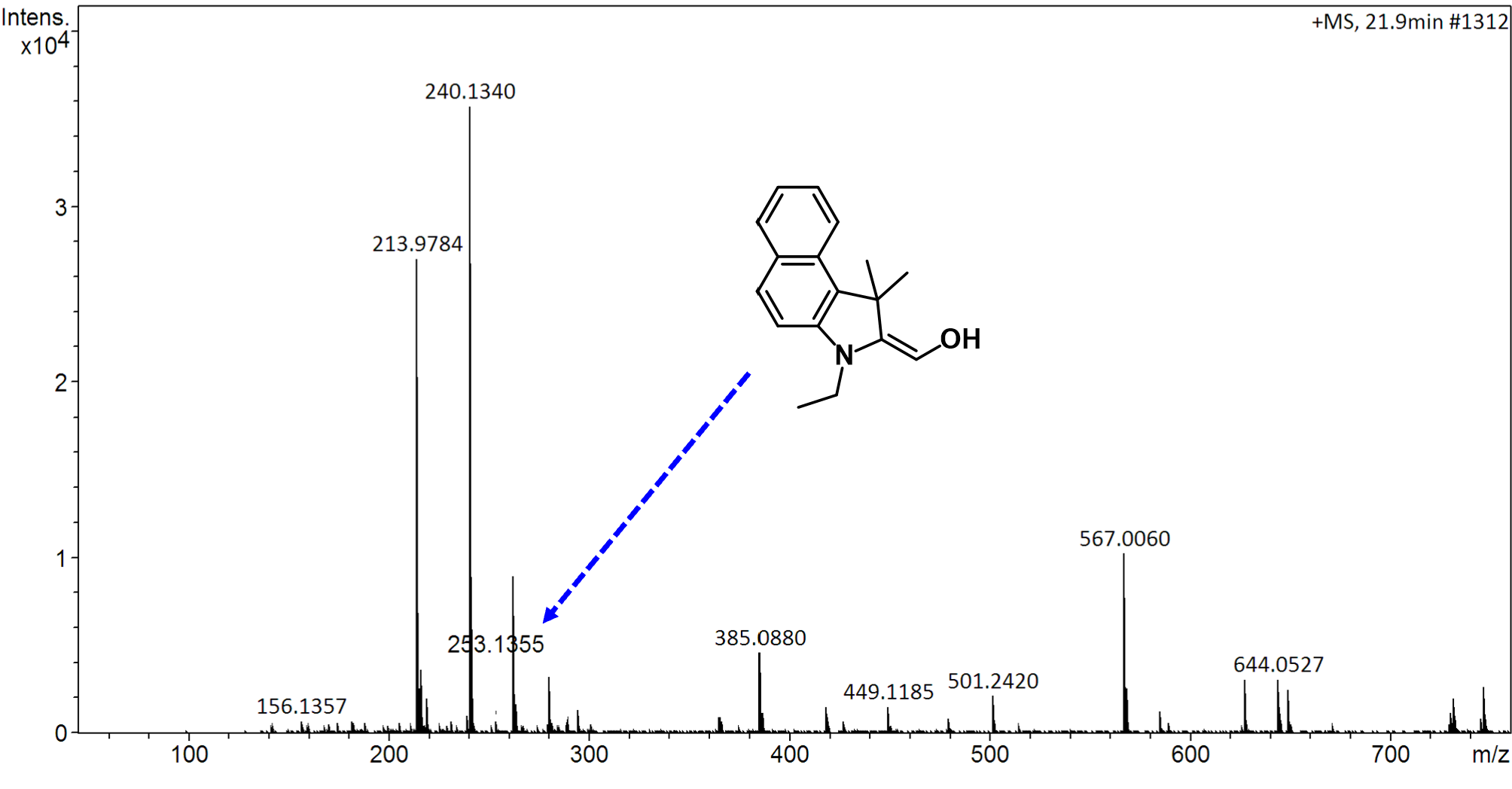


**Figure S50.** LC-MC results of the **Sens2/HABI-5/NPG** system in acetonitrile under 808 nm laser source with an exposure intensity of 714 mW·cm^-2^.


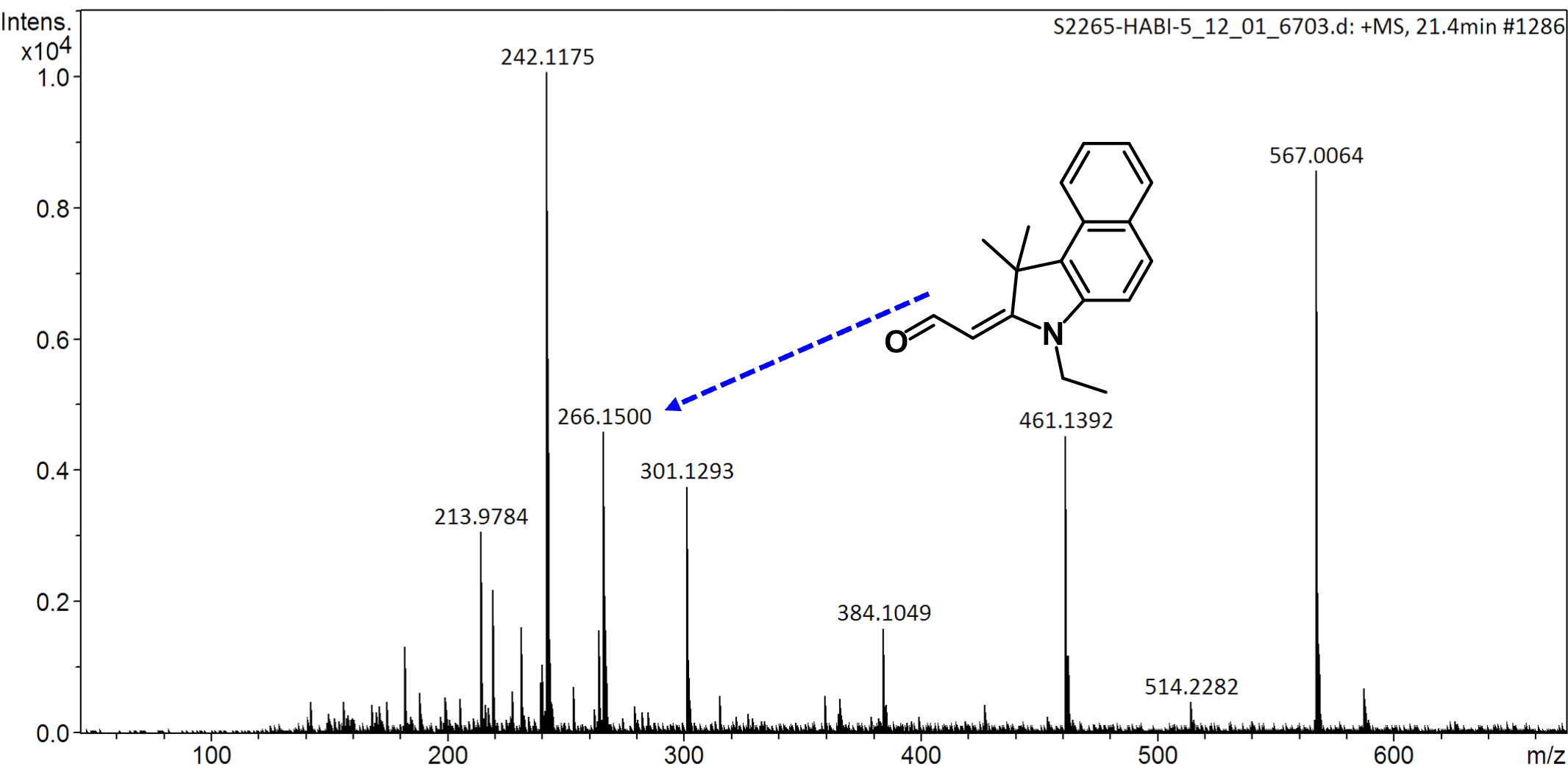


**Figure S51.** LC-MC results of the **Sens2/HABI-5/NPG** system in acetonitrile under 808 nm laser source with an exposure intensity of 714 mW·cm^-2^.


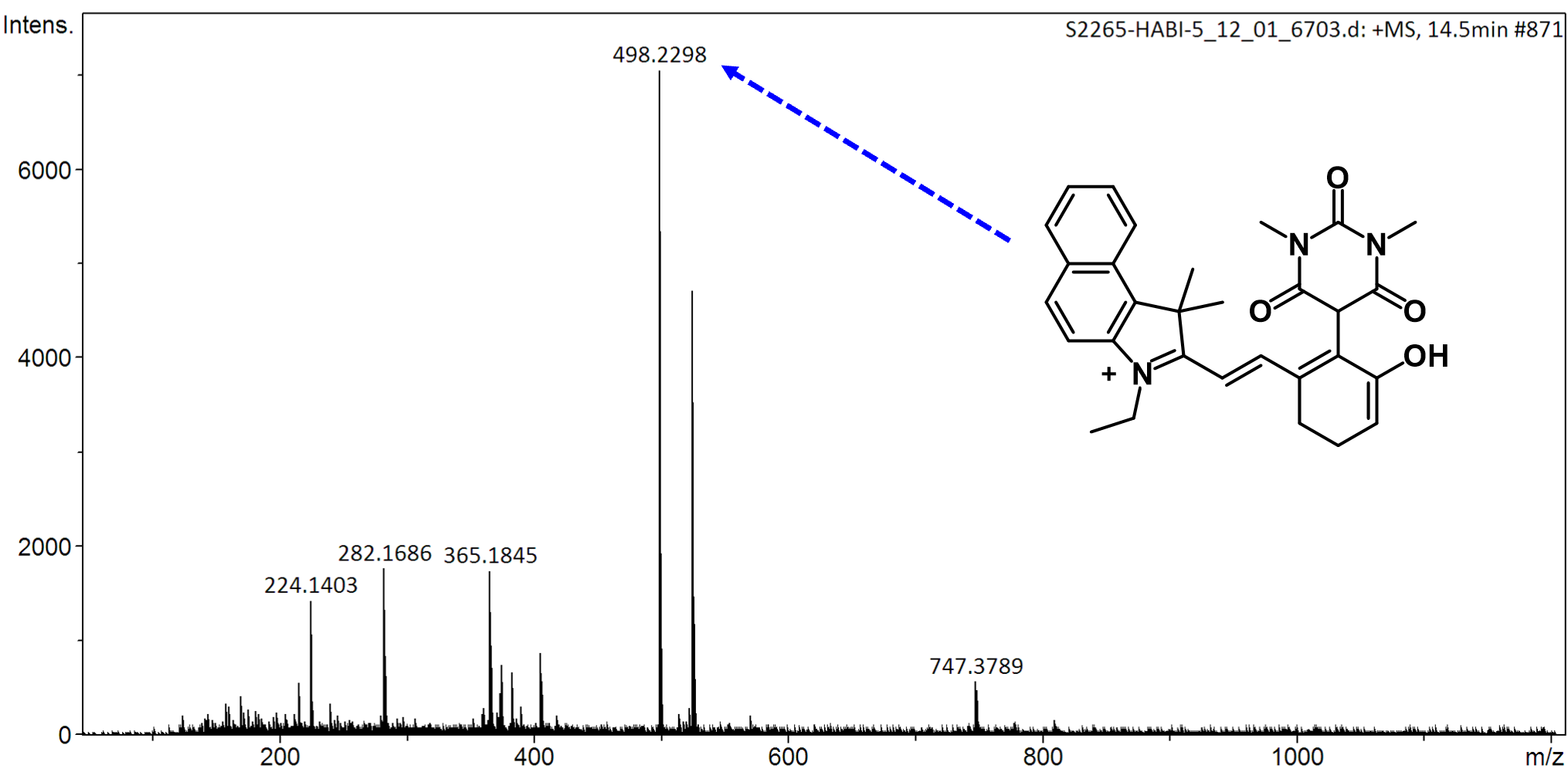


**Figure S52.** LC-MC results of the **Sens2/HABI-5/NPG** system in acetonitrile under 808 nm laser source with an exposure intensity of 714 mW·cm^-2^.


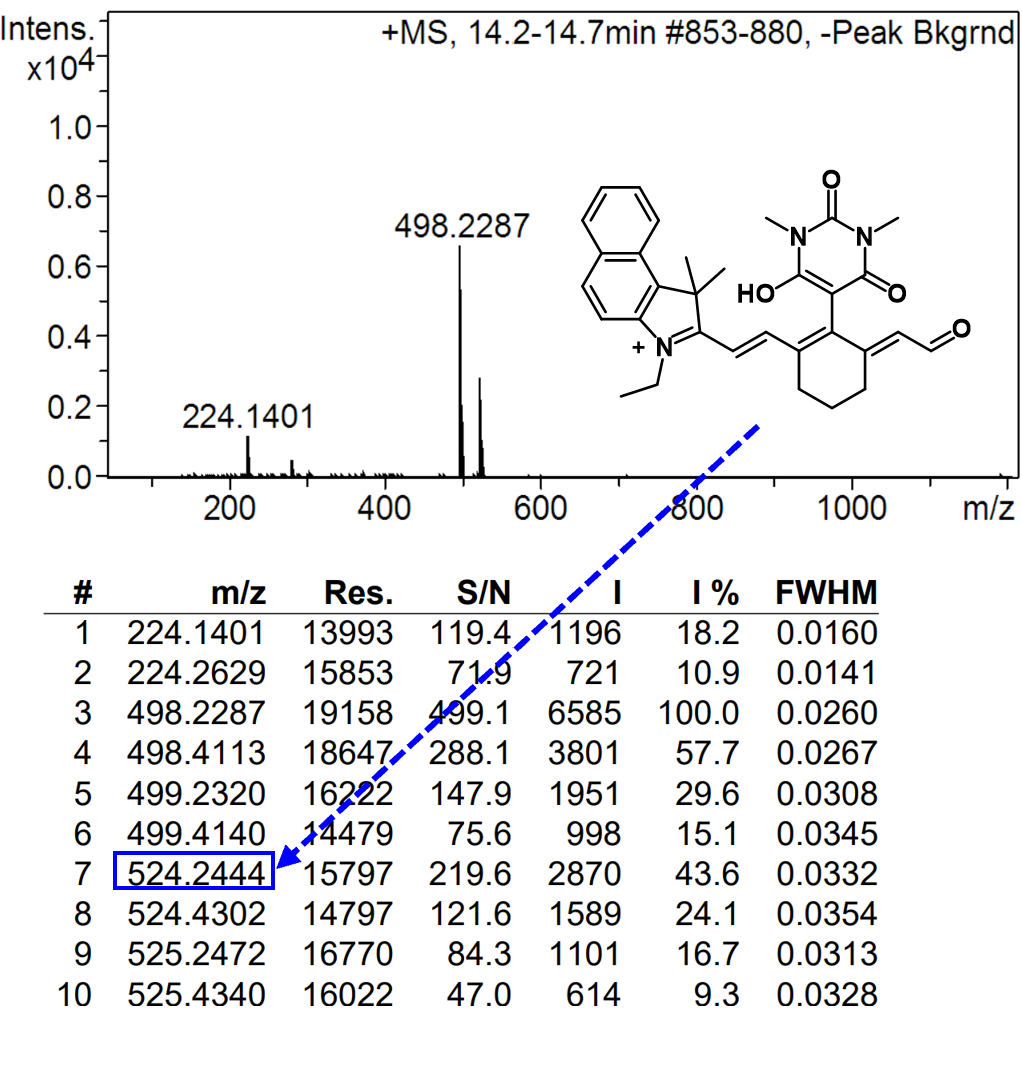


**Figure S53.** LC-MC results of the **Sens2/HABI-5/NPG** system in acetonitrile under 808 nm laser source with an exposure intensity of 714 mW·cm^-2^.


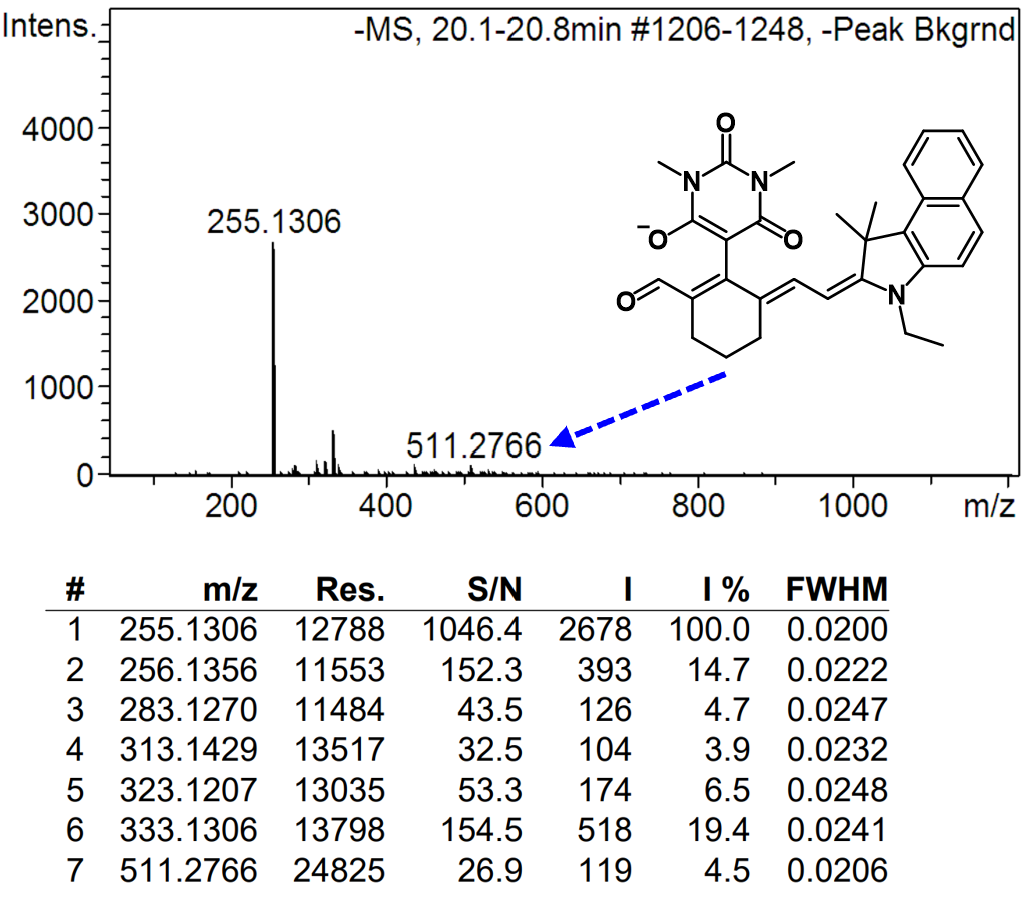


**Figure S54.** LC-MC results of the **Sens2/HABI-5/NPG** system in acetonitrile under 808 nm laser source with an exposure intensity of 714 mW·cm^-2^.


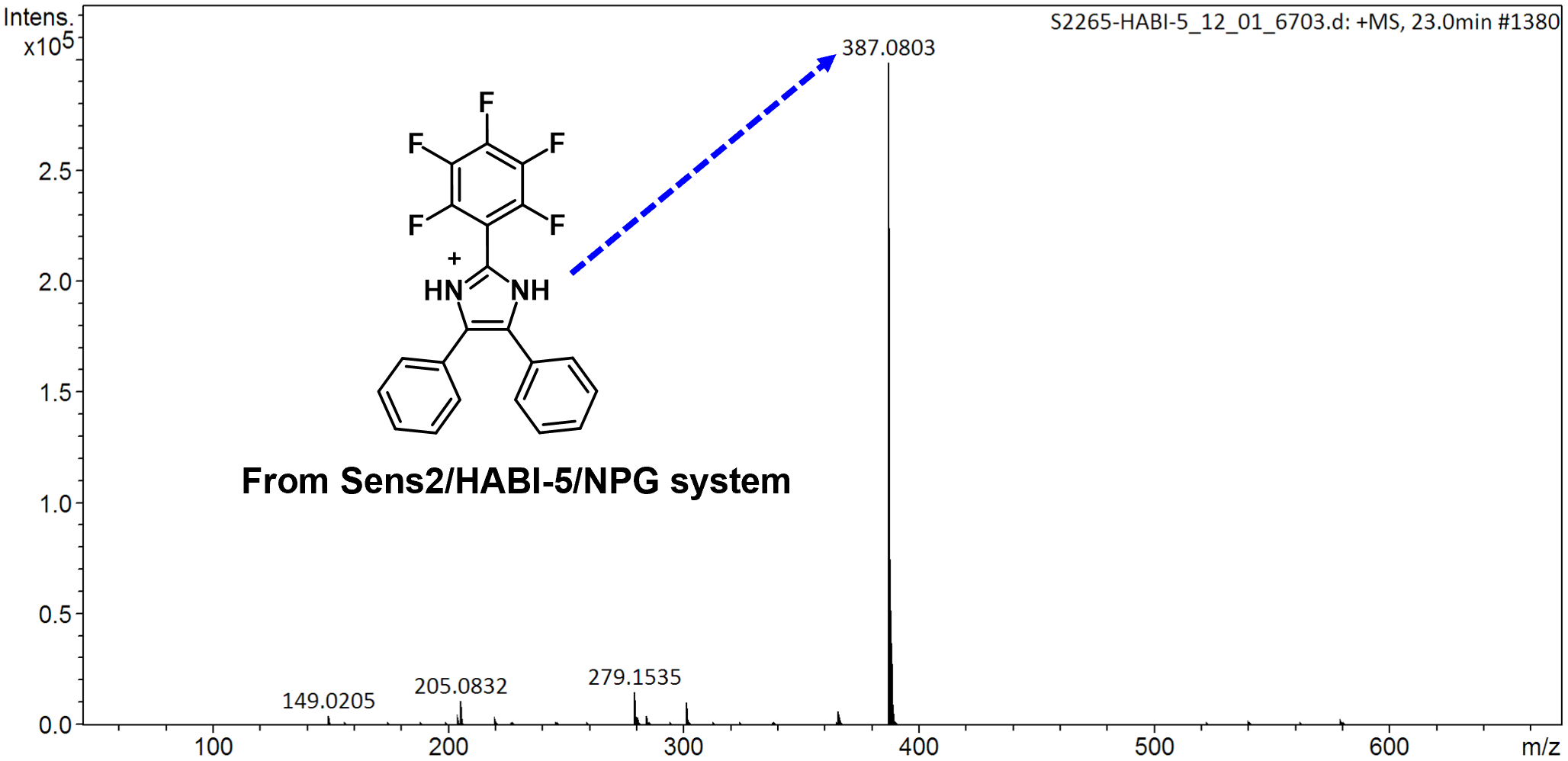


**Figure S55.** LC-MC results of the **Sens2/HABI-5/NPG** system in acetonitrile under 808 nm laser source with an exposure intensity of 714 mW·cm^-2^.


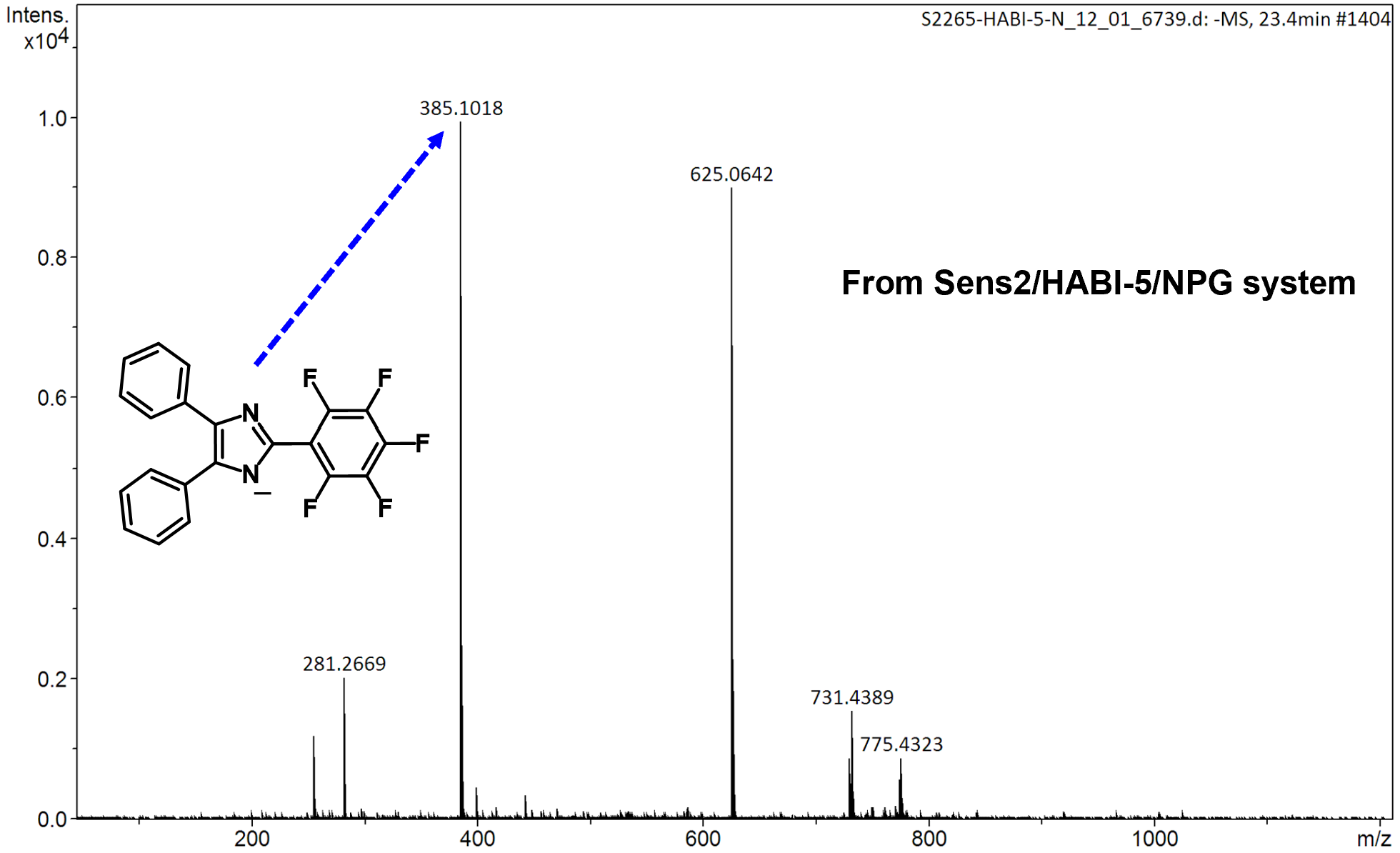


**Figure S56.** LC-MC results of the **Sens2/HABI-5/NPG** system in acetonitrile under 808 nm laser source with an exposure intensity of 714 mW·cm^-2^.


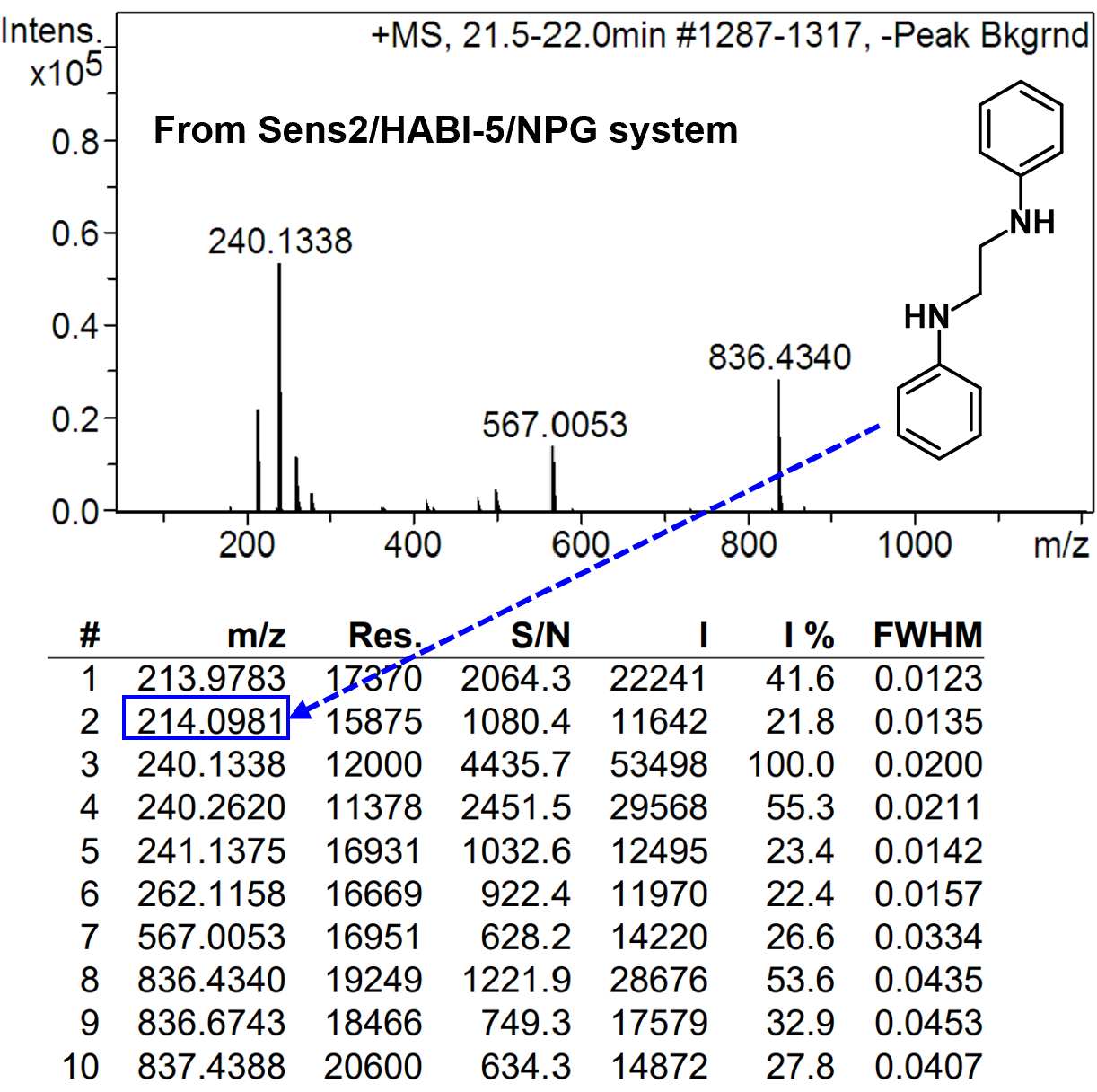


**Figure S57.** LC-MC results of the **Sens2/HABI-5/NPG** system in acetonitrile under 808 nm laser source with an exposure intensity of 714 mW·cm^-2^.

## Preparation and Characterization of Dry Film Photoresist (DFR)


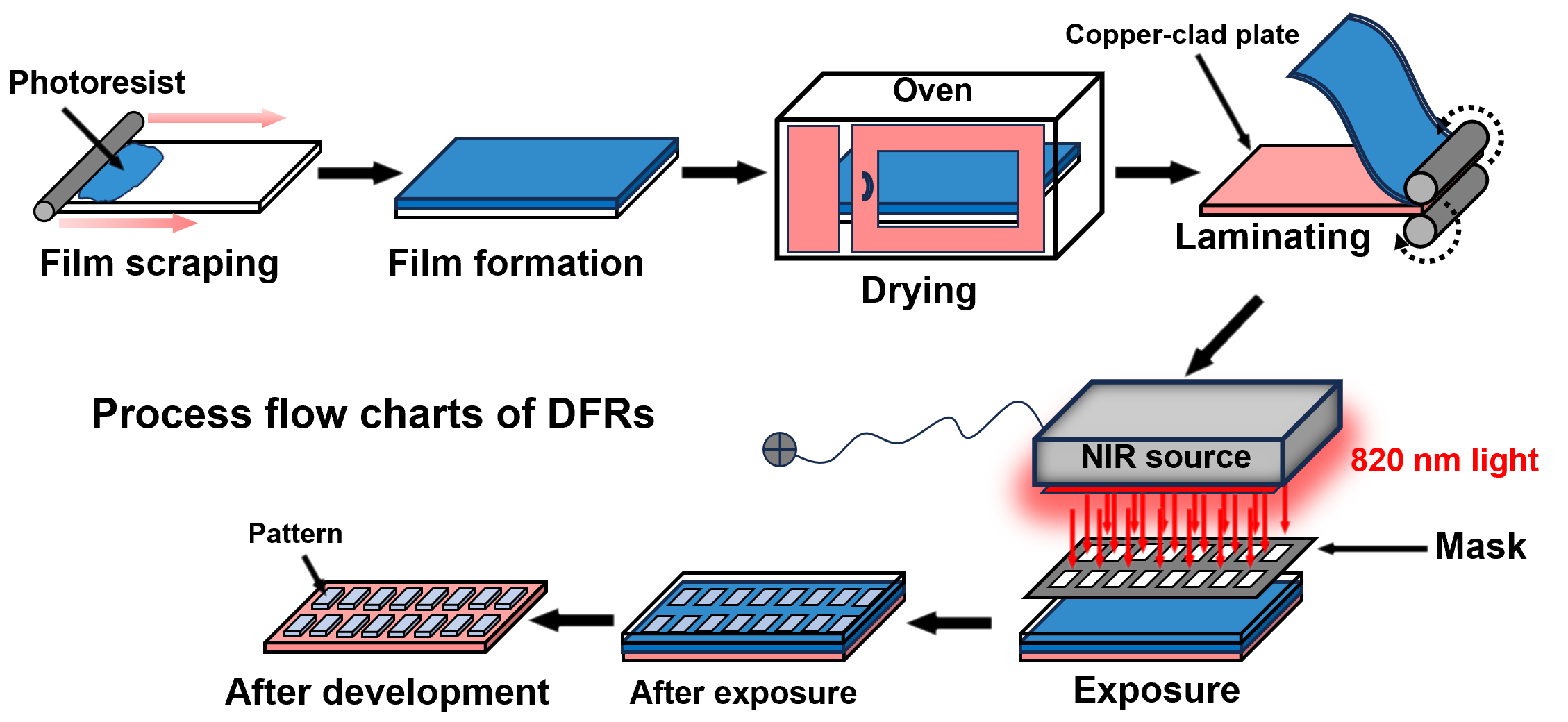


**Figure S58**. The flow chart of preparation and characterization of **DFR**s.


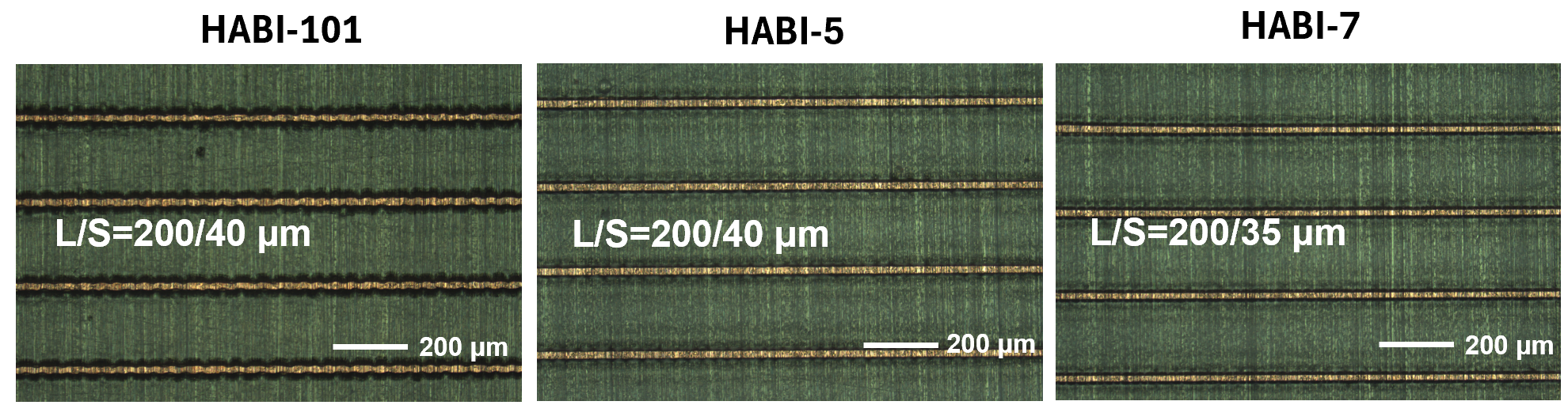


**Figure S59**. The L/S (L: line width, S: line spacing) of NIR **DFR**s containing **Sens**/**HABI**s/**NPG** systems under intense irradiation at 820 nm with a LED source (1.5 W cm^−2^) for 120 s.


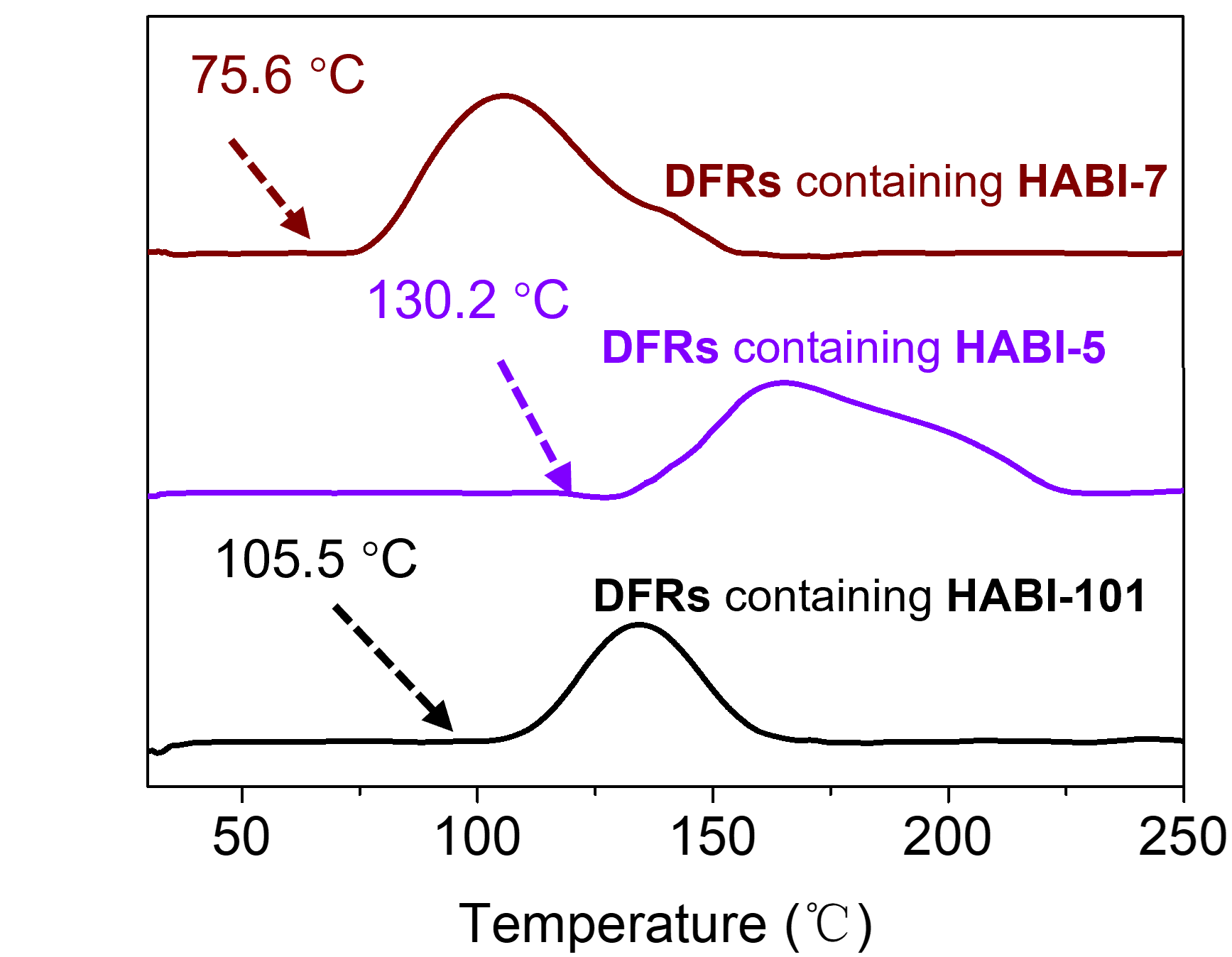


**Figure S60**. DSC of **DFR**s containing **HABI-101**, **HABI-5** or **HABI-7** at a rate of 10 K min^−1^.


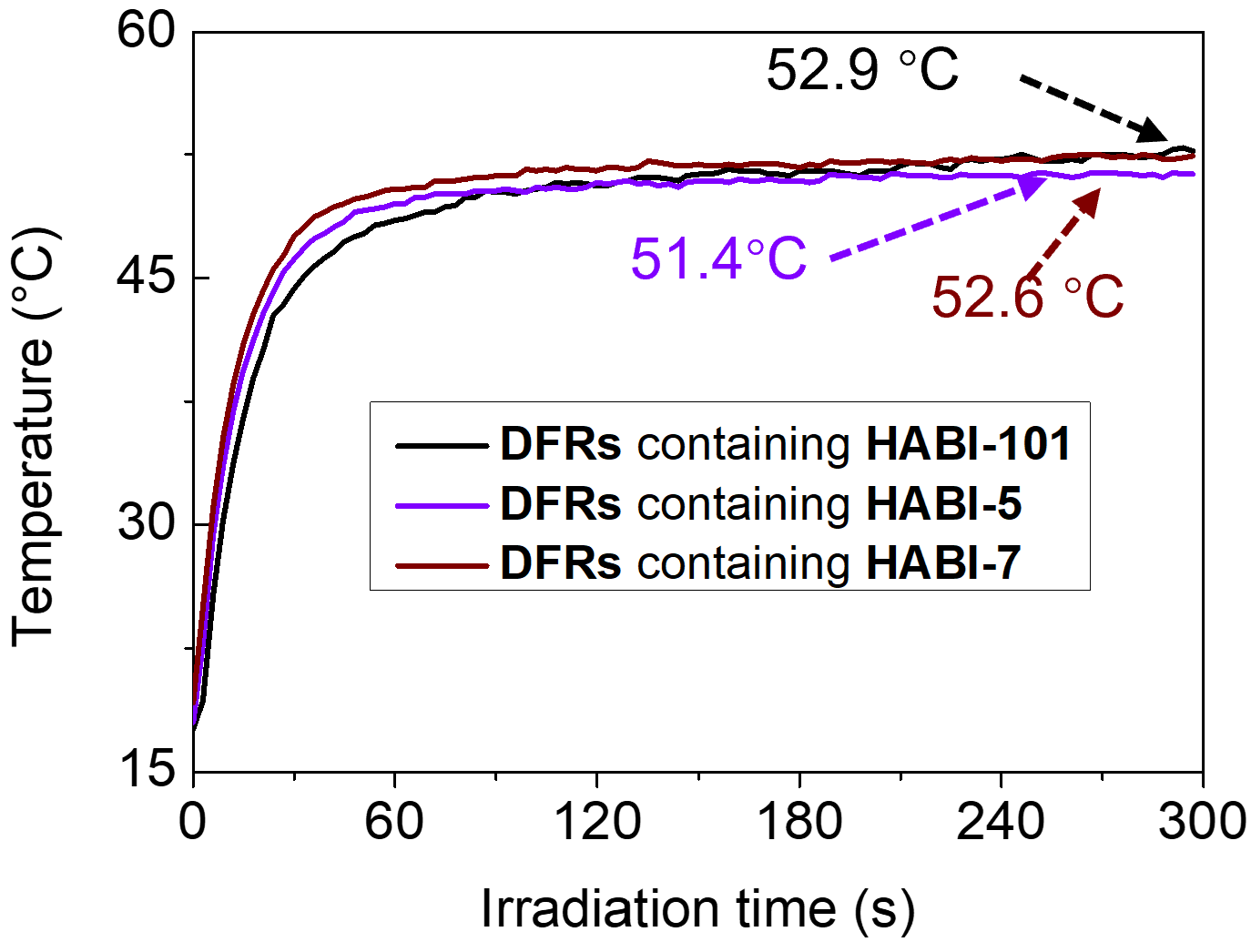


**Figure S61**. The temperature change curves during the **DFR**s exposure process under irradiation upon 820 nm LED (1.5 W cm^-2^) recorded by thermal imaging camera.


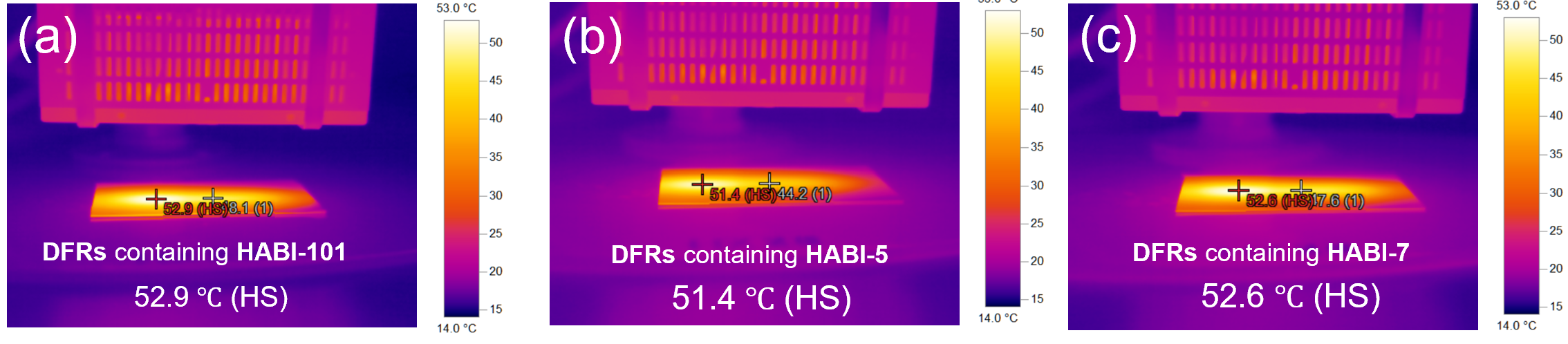


**Figure S62**. Thermal imaging pictures of the hottest (HS) temperatures during the **DFR**s exposure process under irradiation upon 820 nm LED (1.5 W cm^-2^) recorded by thermal imaging camera; (a) **DFR**s containing **HABI-101,** (b) **DFR**s containing **HABI-5**, (c) **DFR**s containing **HABI-7**.

## Nuclear Magnetic Resonance Data


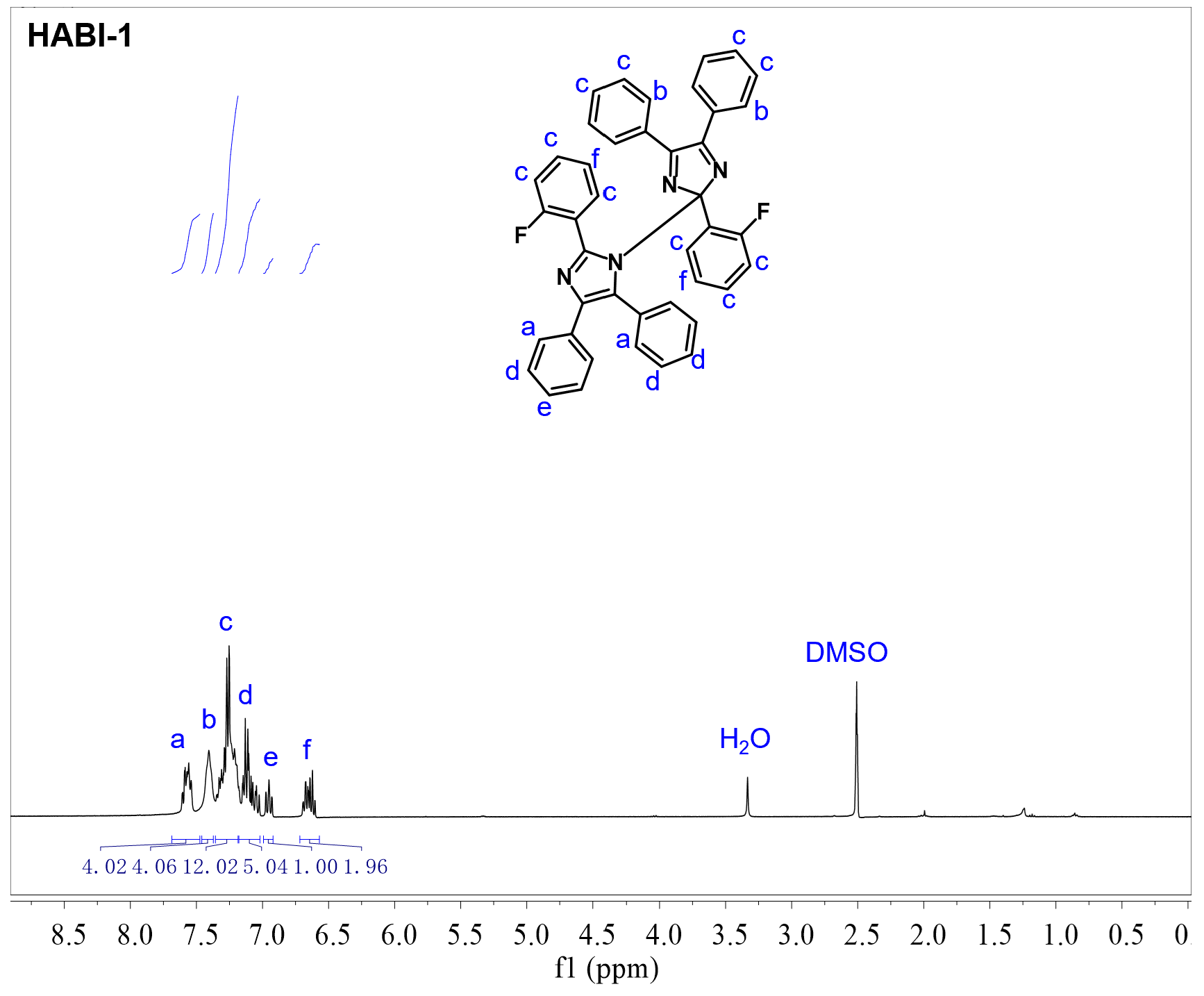


**Figure S63.** ^1^H NMR spectrum of 2,2'-Bis(2-fluorophenyl)-4,4',5,5'-tetraphenyl-2'H-1,2'-biimidazole (**HABI-1**)


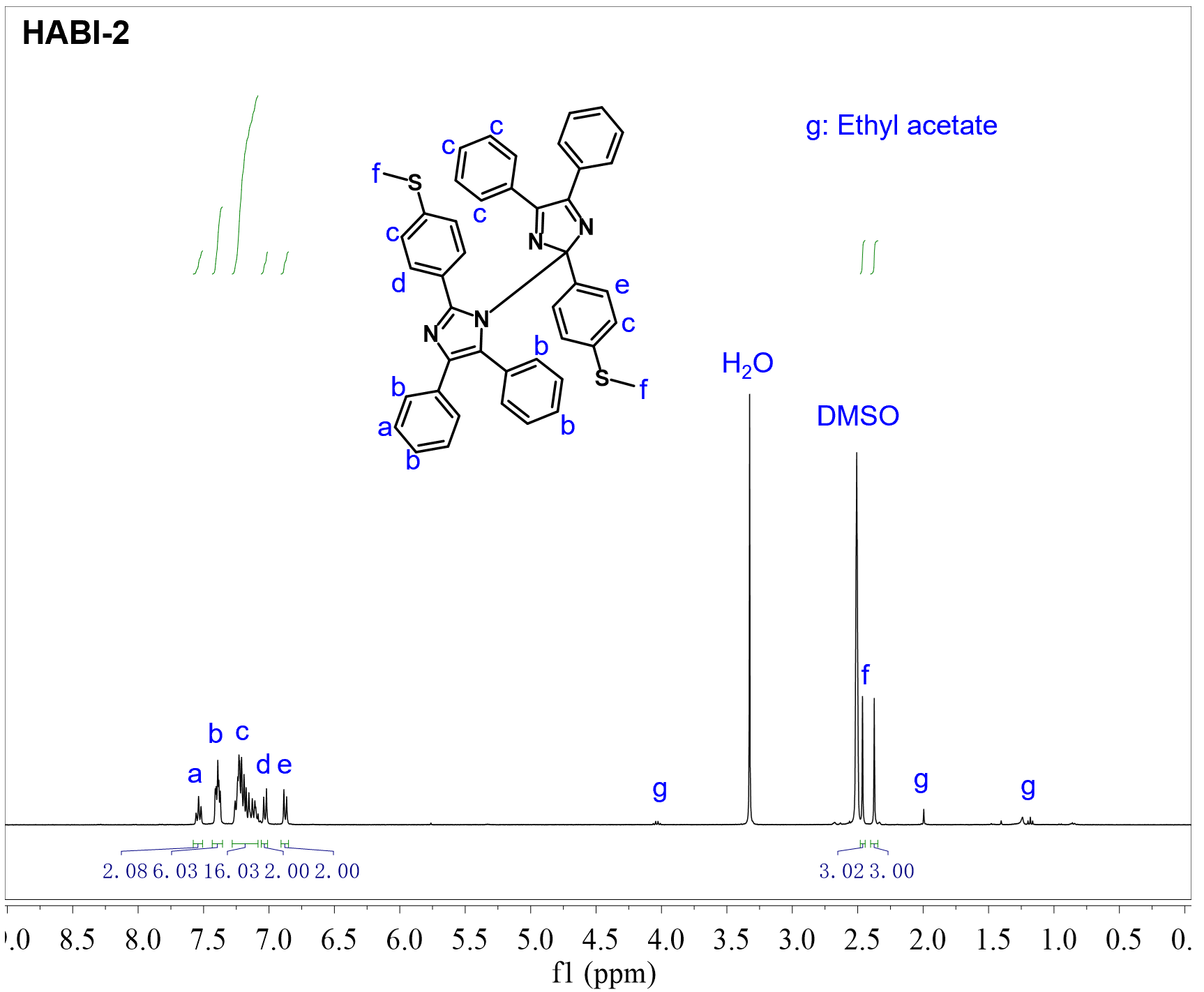


**Figure S64.** ^1^H NMR spectrum of 2,2'-bis(4-(methylthio)phenyl)-4,4',5,5'-tetraphenyl-2'H-1,2'-biimidazole (**HABI-2**)


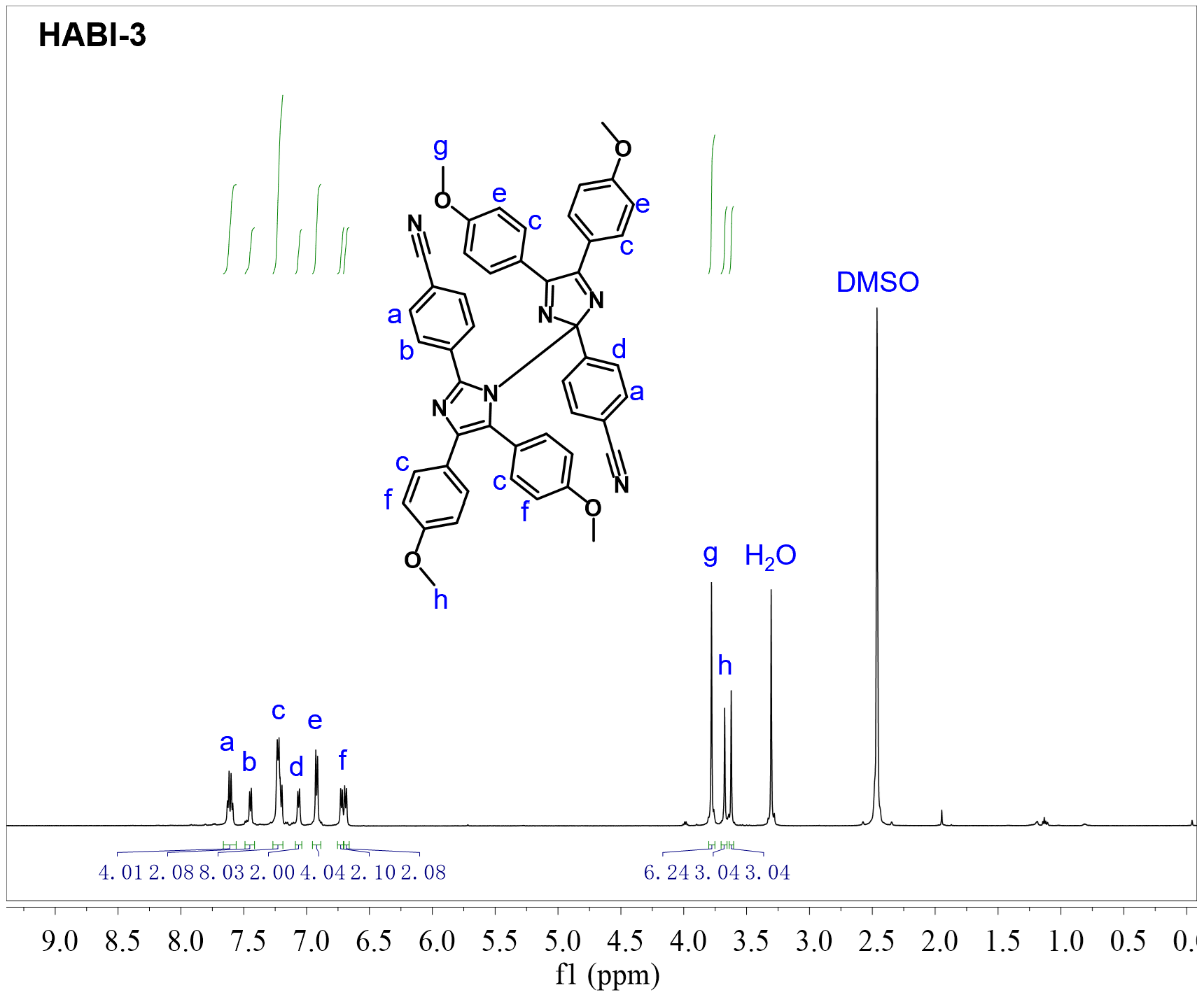


**Figure S65.** ^1^H NMR spectrum of 4,4'-(4,4',5,5'-tetrakis(4-methoxyphenyl)-2'H-[1,2'-biimidazole]-2,2'-diyl) dibenzonitrile (**HABI-3**)


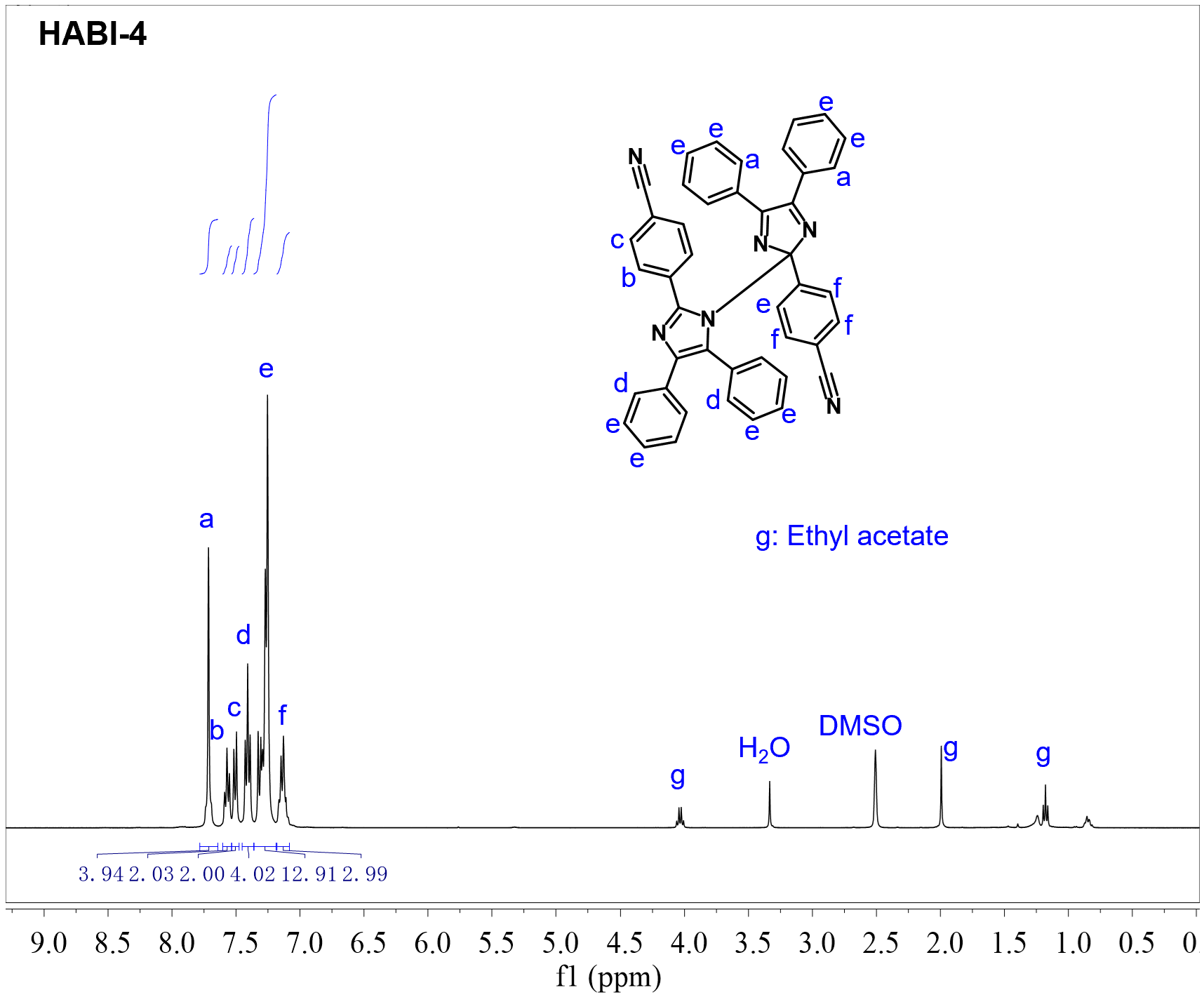


**Figure S66.** ^1^H NMR spectrum of 4,4'-(4,4',5,5'-tetraphenyl-2'H-[1,2'-biimidazole]-2,2'-diyl) dibenzonitrile (**HABI-4**)


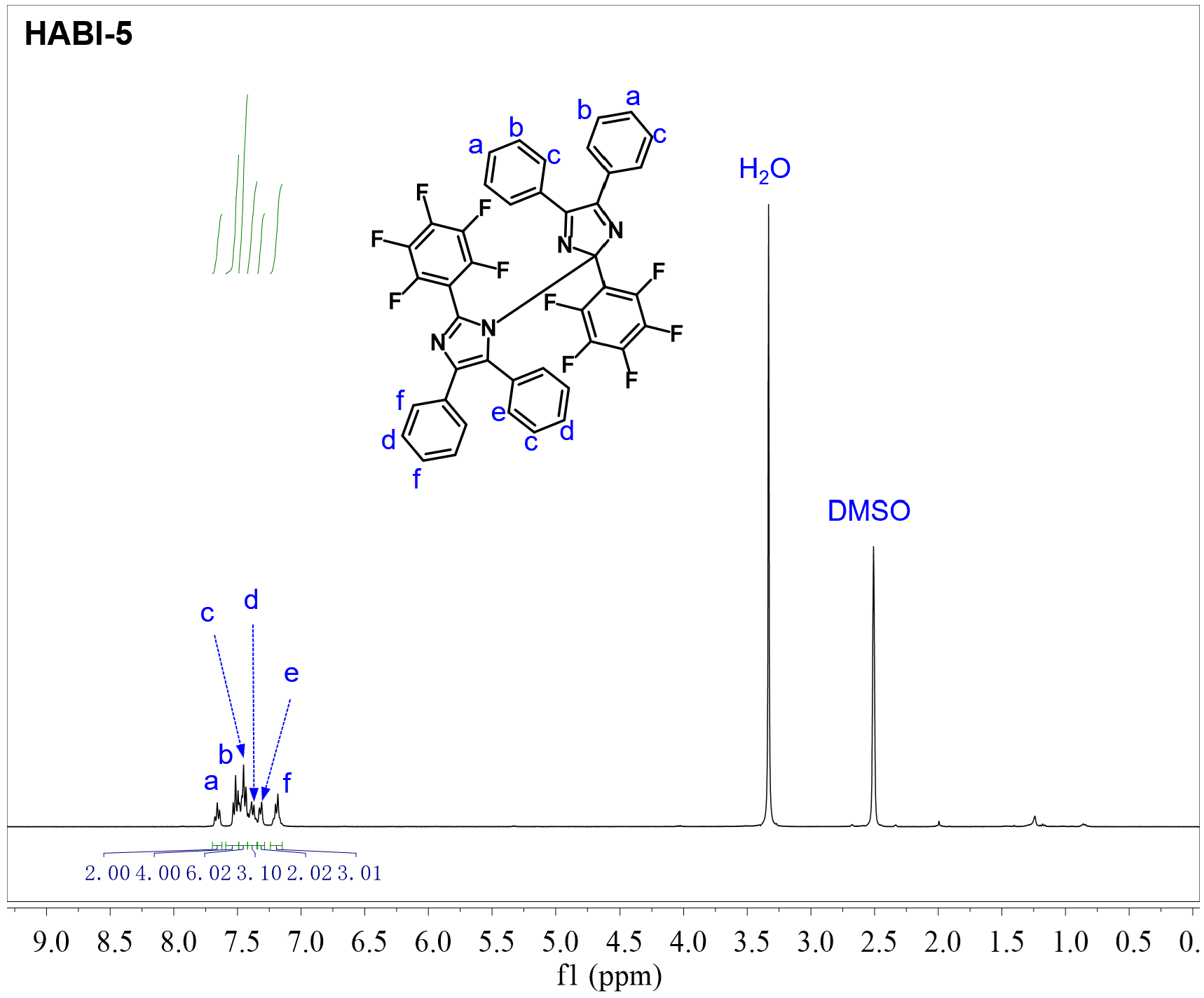


**Figure S67.** ^1^H NMR spectrum of 2,2'-bis(perfluorophenyl)-4,4',5,5'-tetraphenyl-2'H-1,2'-biimidazole (**HABI-5**)


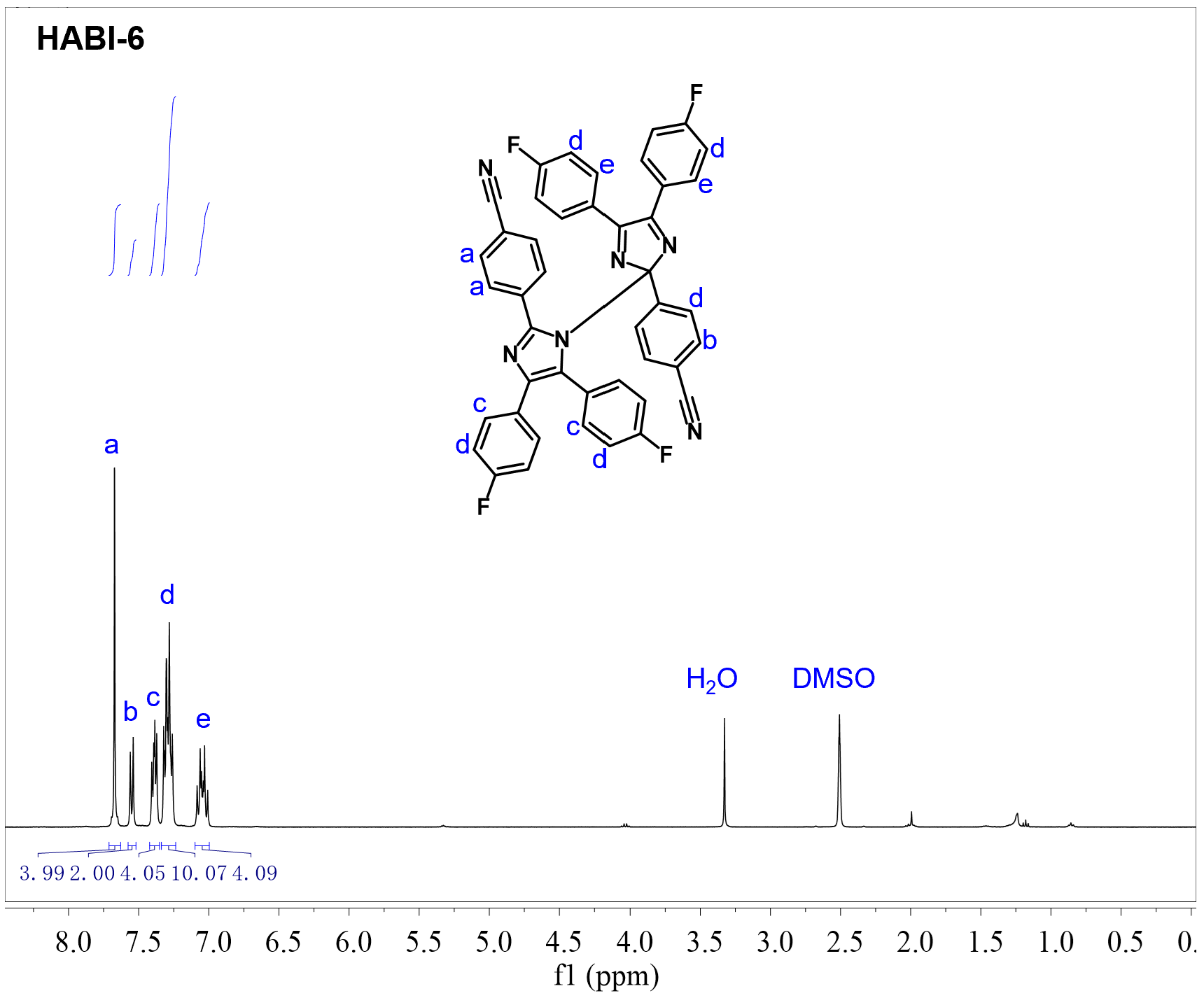


**Figure S68.** ^1^H NMR spectrum of 4,4'-(4,4',5,5'-tetrakis(4-fluorophenyl)-2'H-[1,2'-biimidazole]-2,2'-diyl)dibenzonitrile (**HABI-6**)


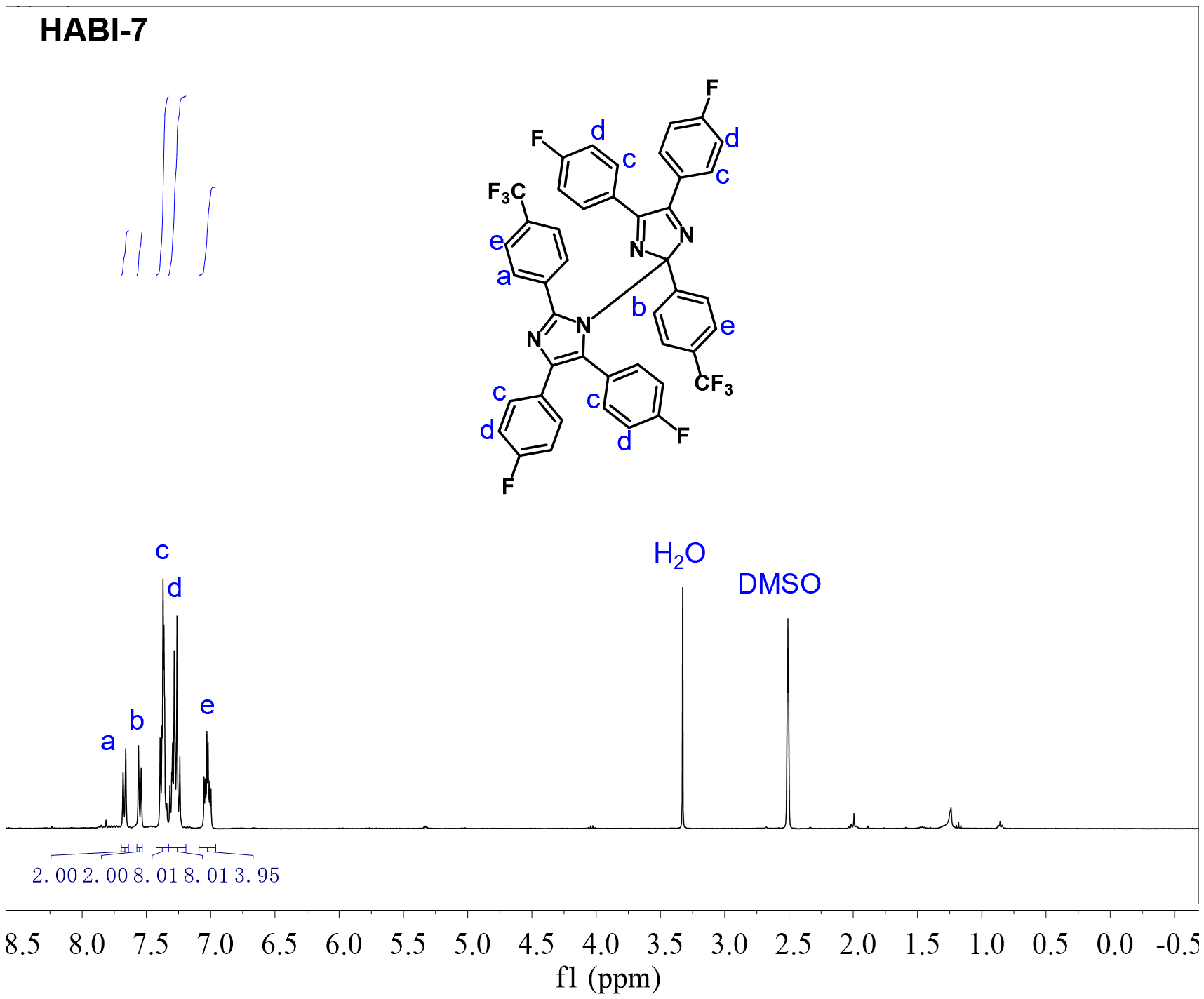


**Figure S69.** ^1^H NMR spectrum of 4,4',5,5'-tetrakis(4-fluorophenyl)-2,2'-bis(4-(trifluoromethyl)phenyl)-2'H-1,2'-biimidazole (**HABI-7**)

## Mass Spectrometry Data


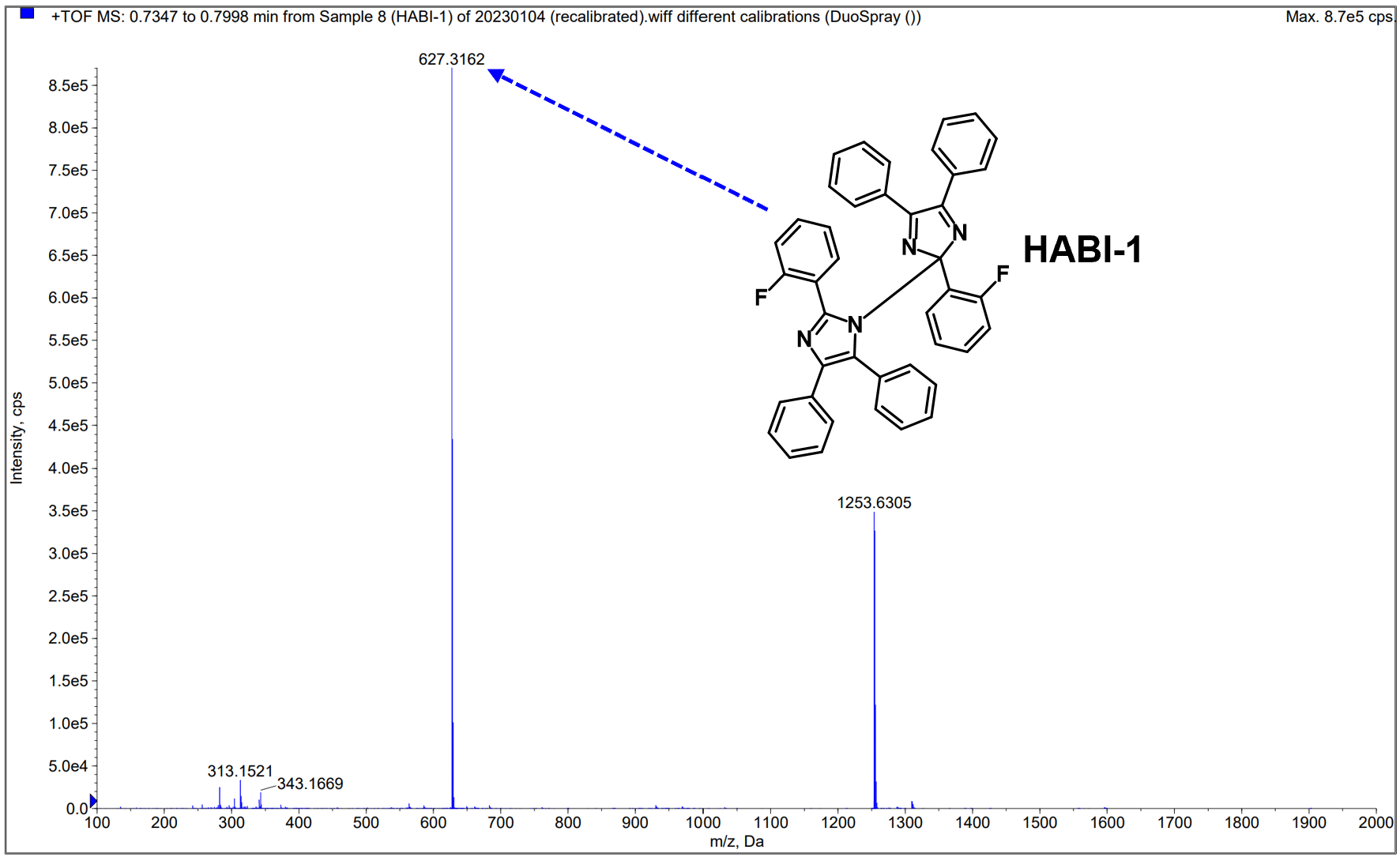


**Figure S70.** HRMS result of the acetonitrile solution of **HABI-1** (0.5 g L^-1^).


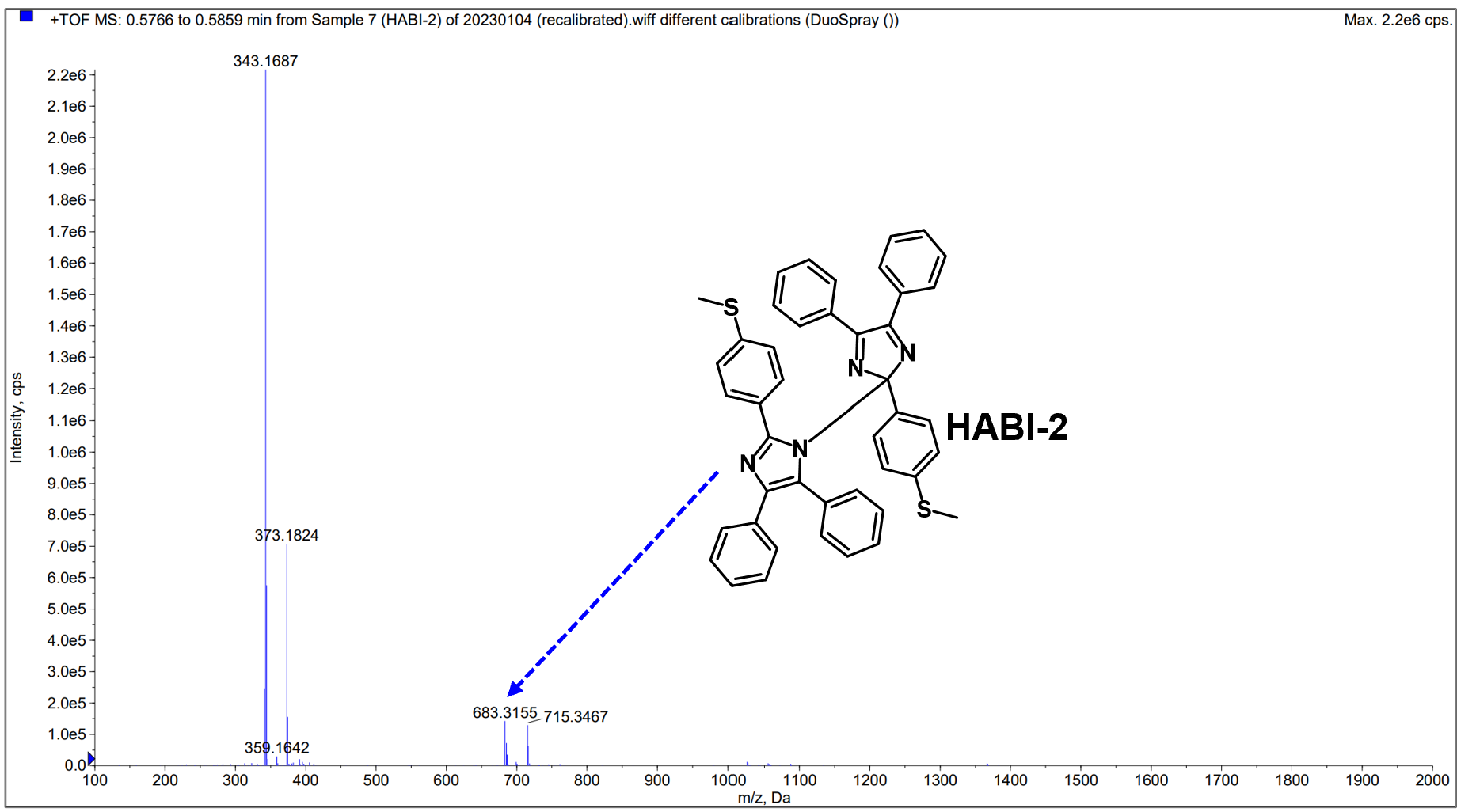


**Figure S71.** HRMS result of the acetonitrile solution of **HABI-2** (0.5 g L^-1^).


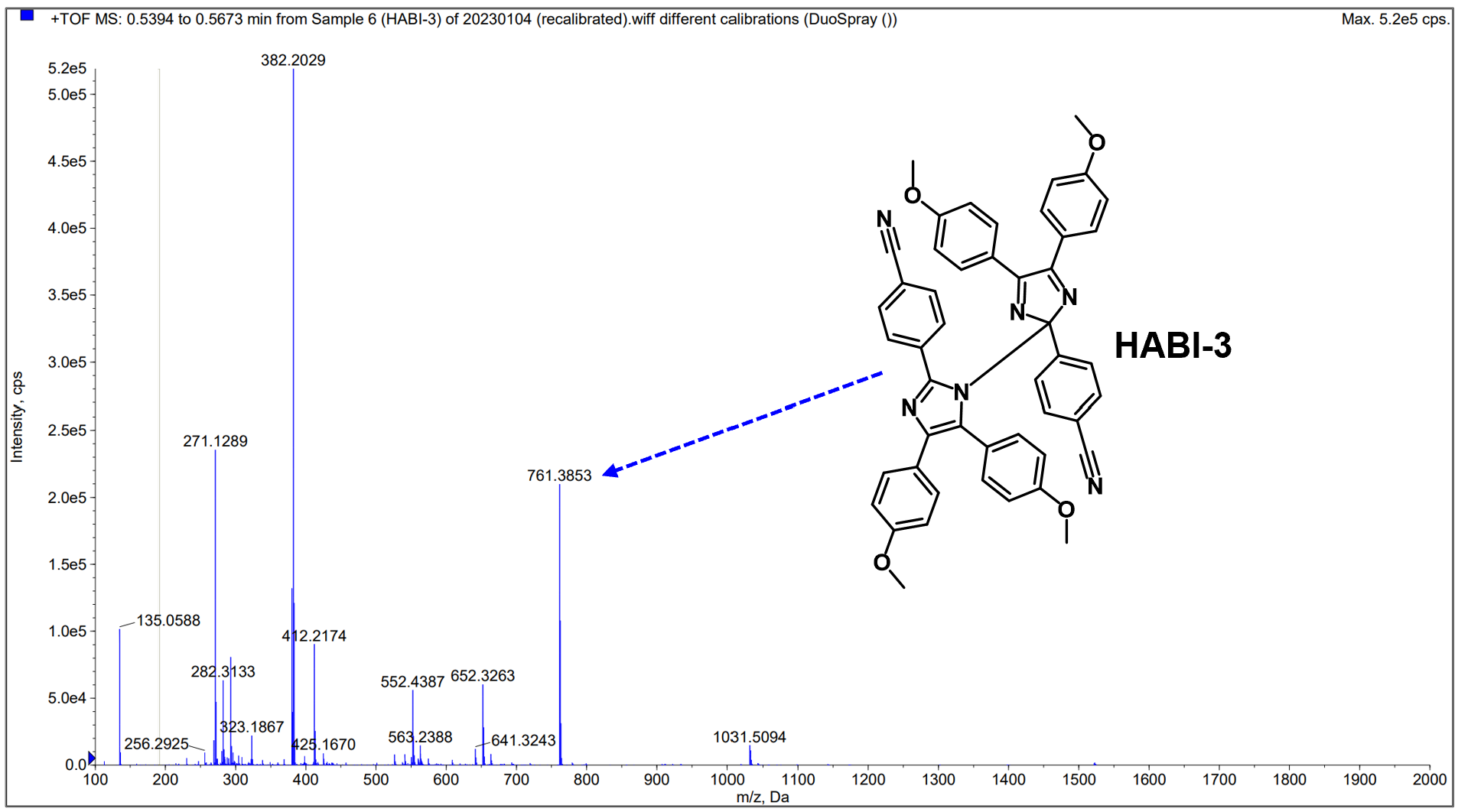


**Figure S72.** HRMS result of the acetonitrile solution of **HABI-3** (0.5 g L^-1^).


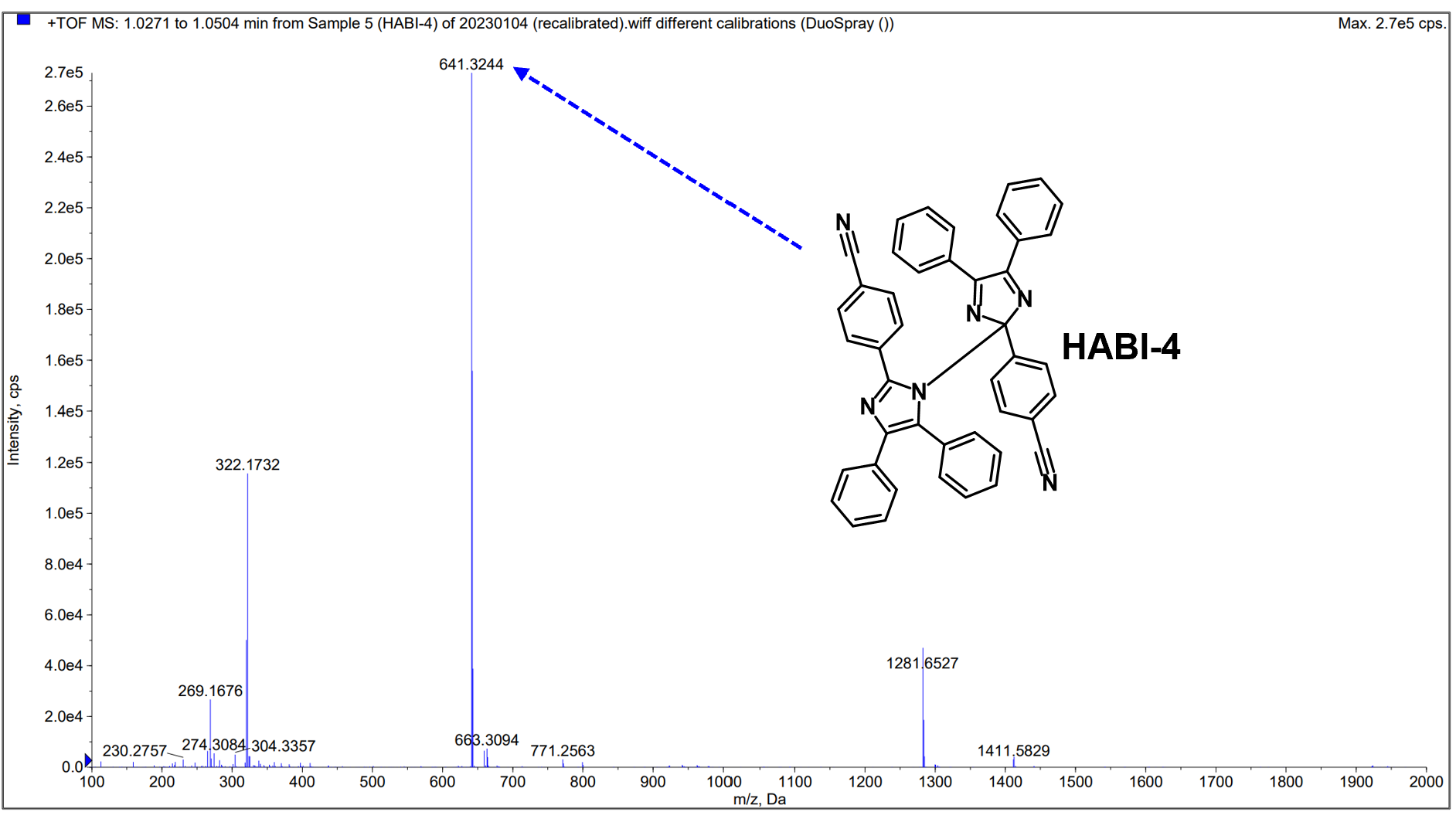


**Figure S73.** HRMS result of the acetonitrile solution of **HABI-4** (0.5 g L^-1^).


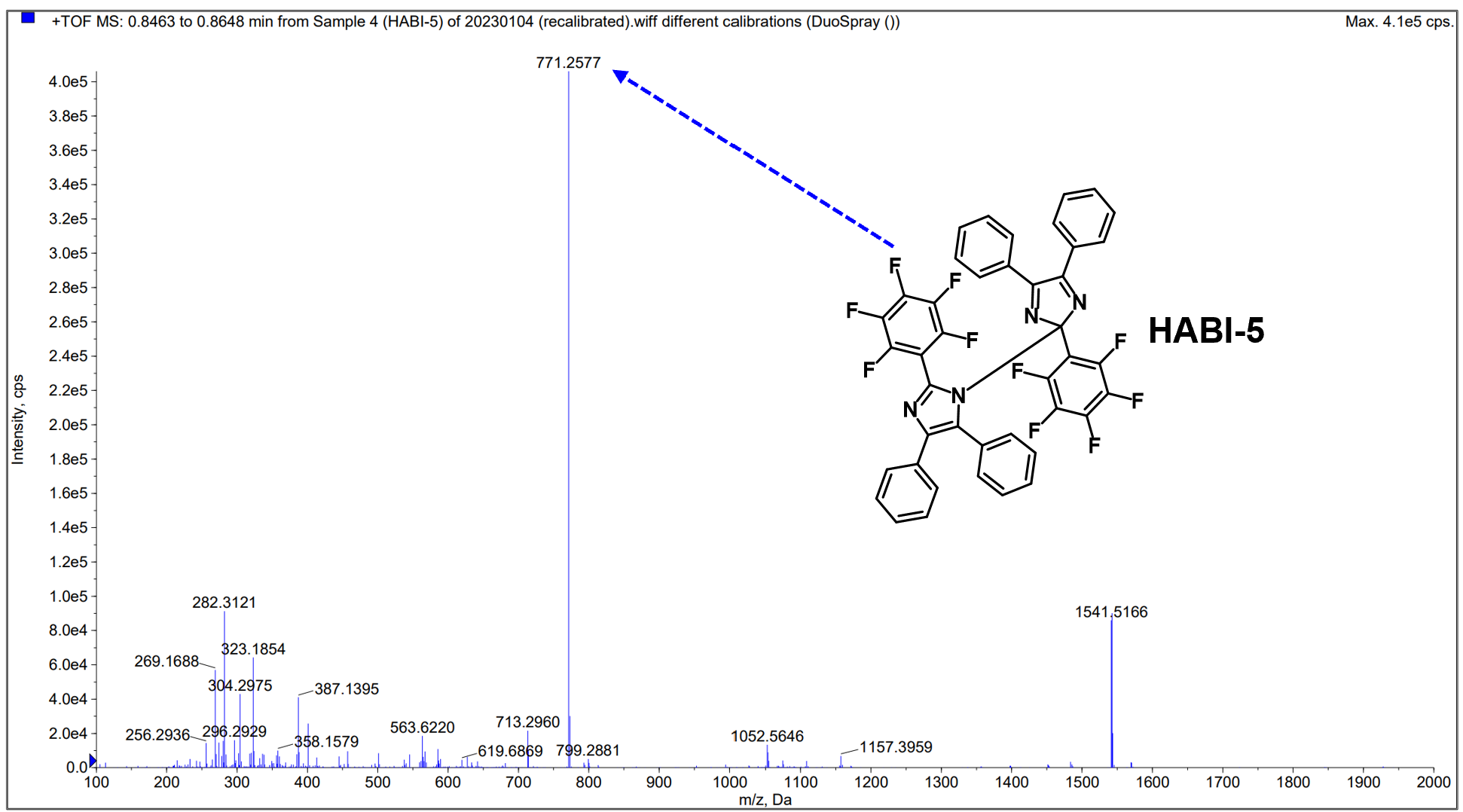


**Figure S74.** HRMS result of the acetonitrile solution of **HABI-5** (0.5 g L^-1^).


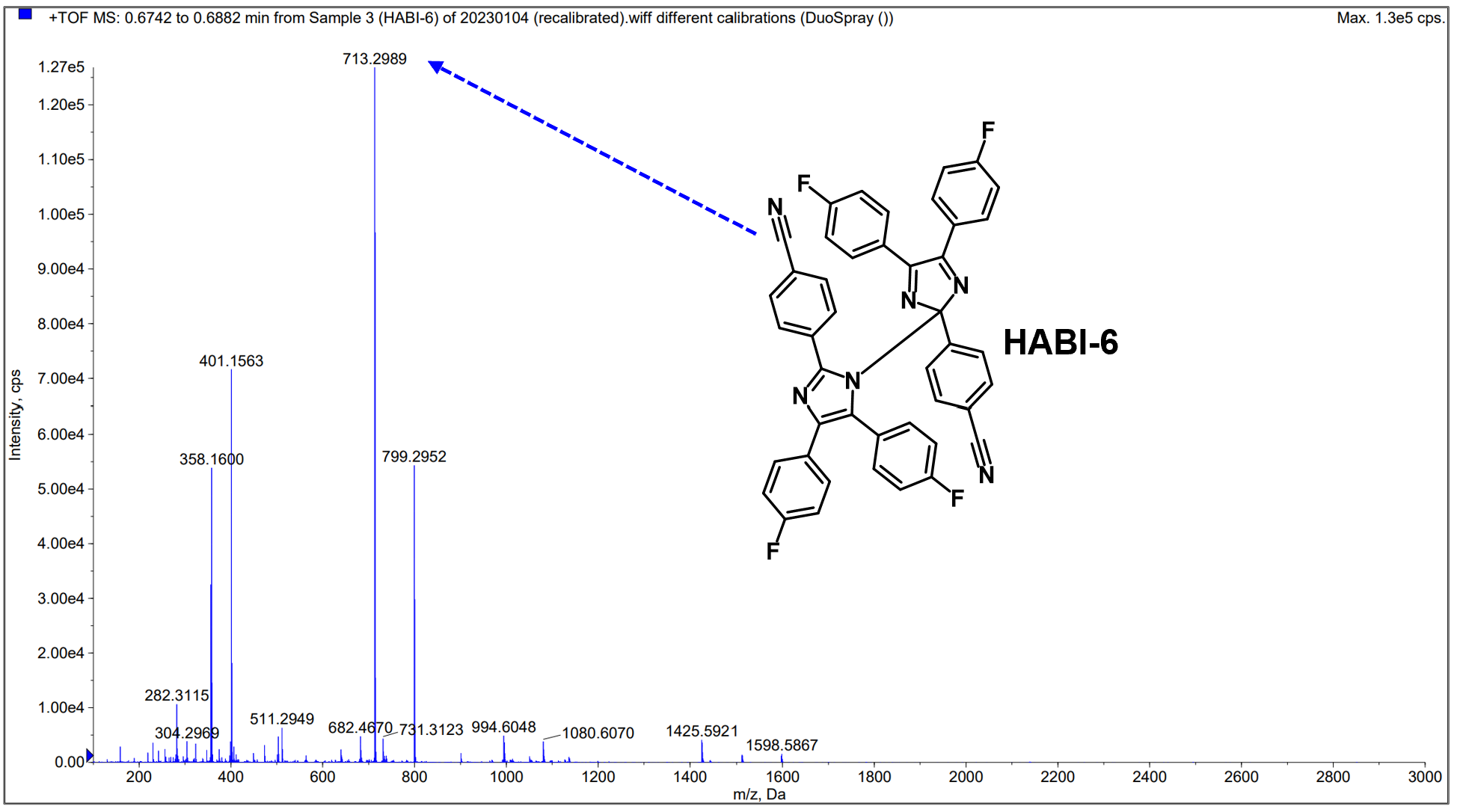


**Figure S75.** HRMS result of the acetonitrile solution of **HABI-6** (0.5 g L^-1^).


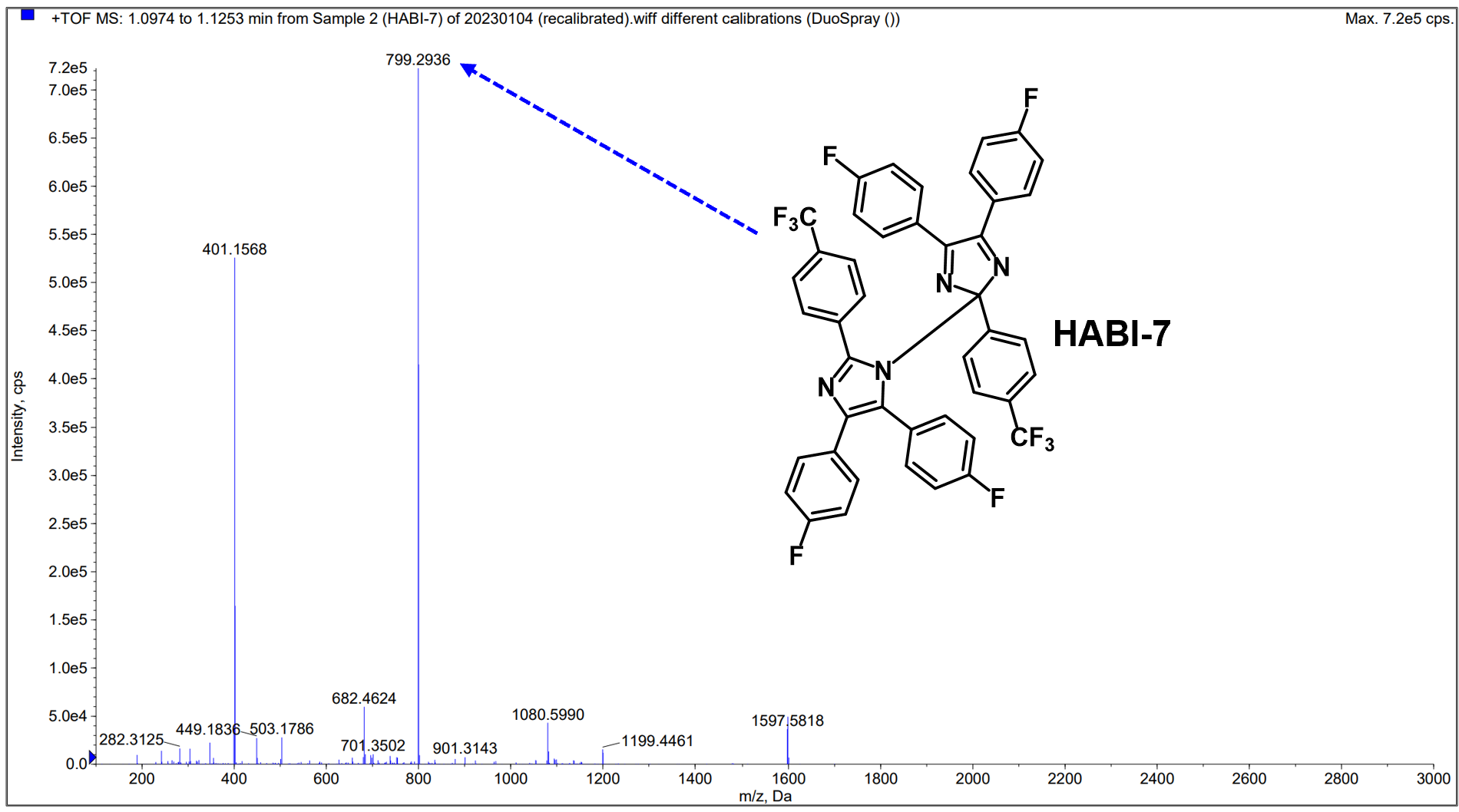


**Figure S76.** HRMS result of the acetonitrile solution of **HABI-7** (0.5 g L^-1^).


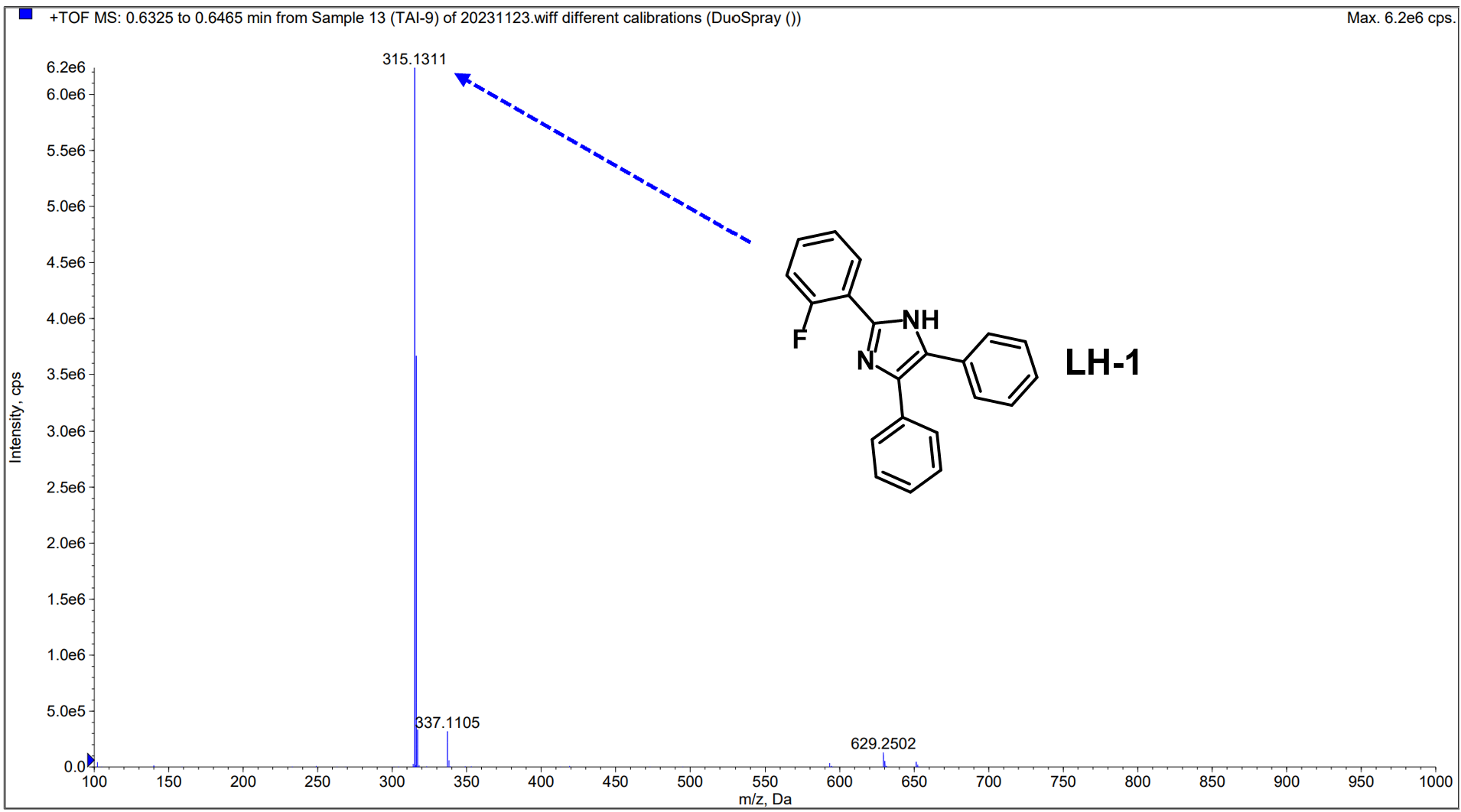


**Figure S77.** HRMS result of the acetonitrile solution of **LH-1** (0.5 g L^-1^).


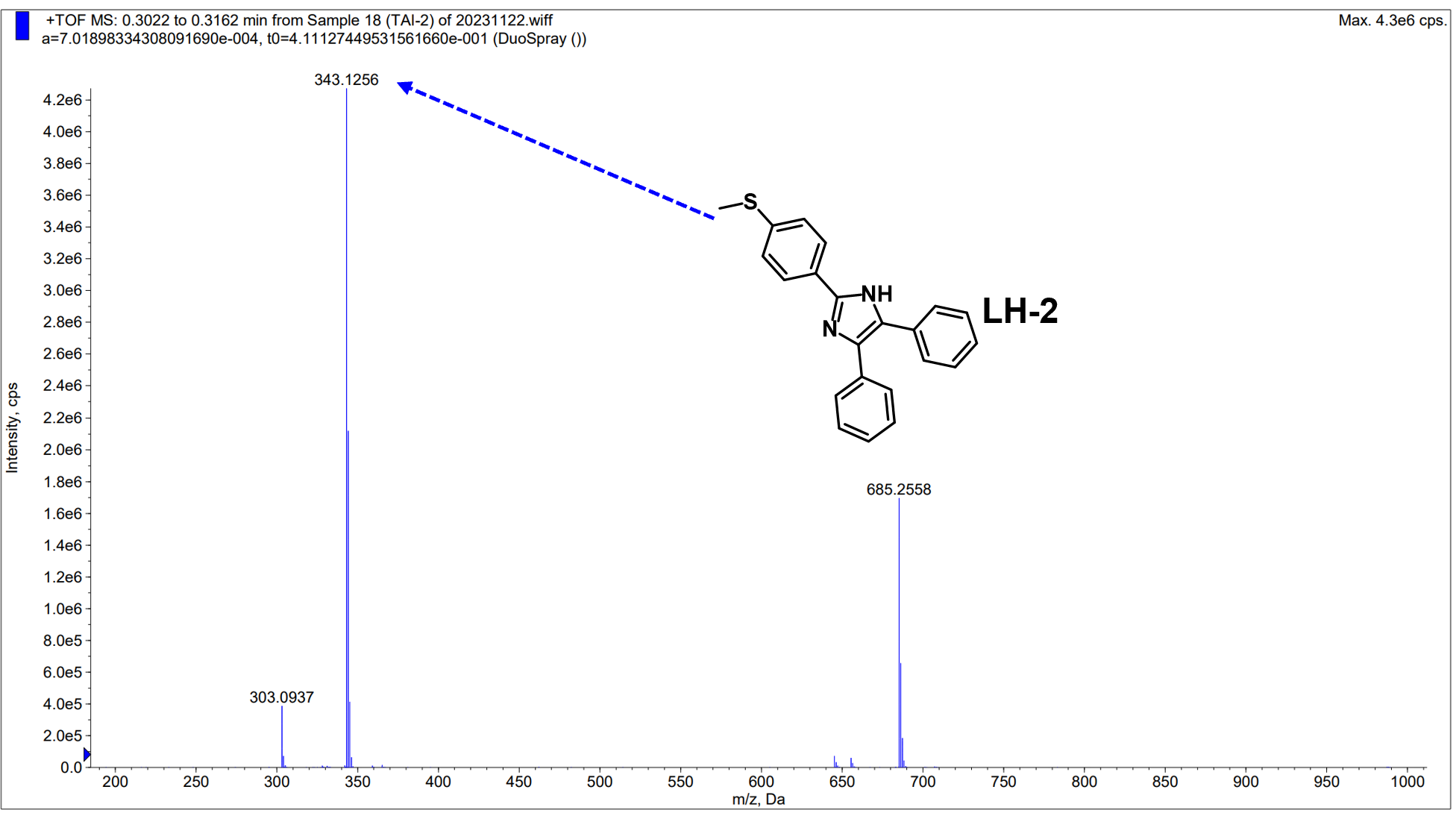


**Figure S78.** HRMS result of the acetonitrile solution of **LH-2** (0.5 g L^-1^).


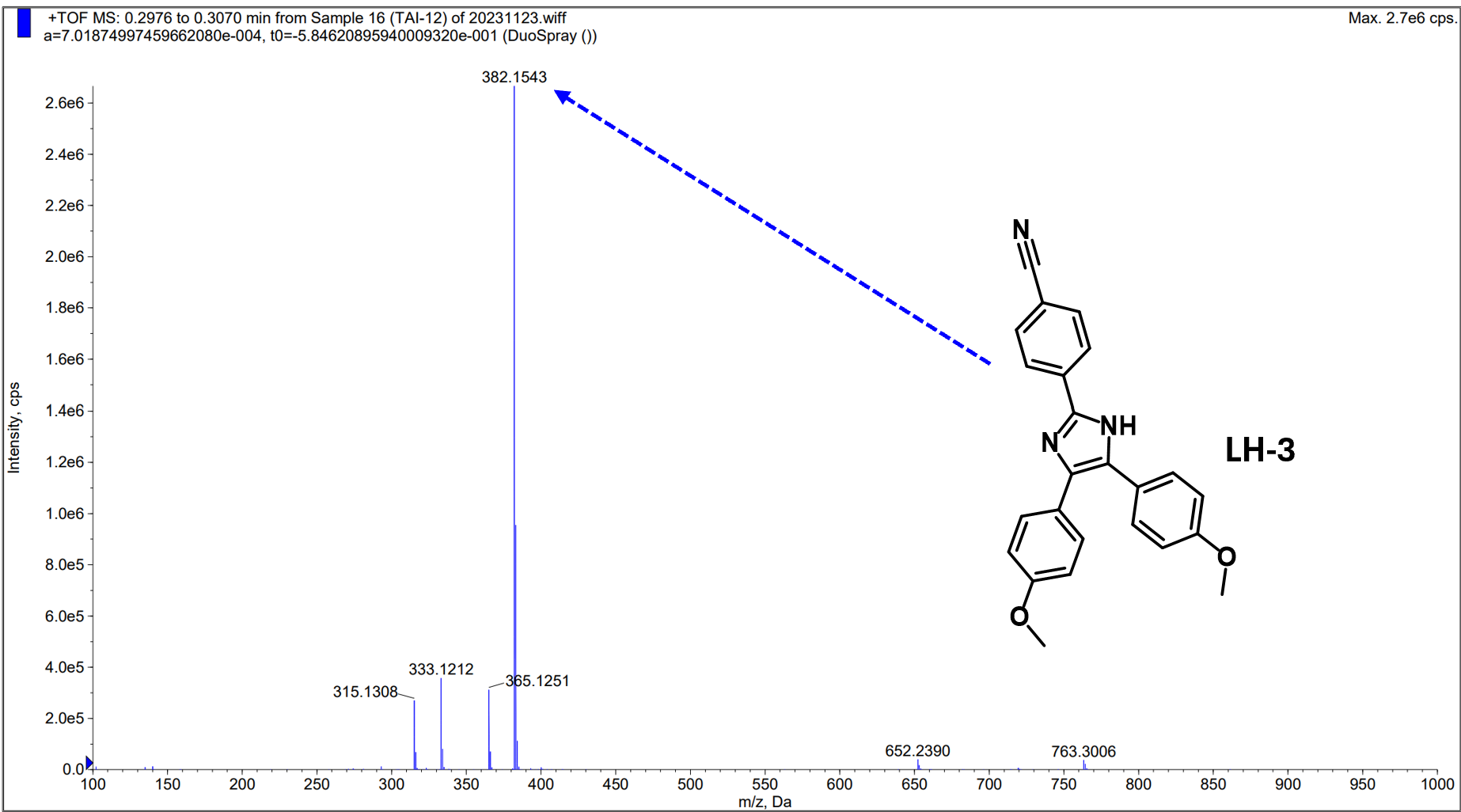


**Figure S79.** HRMS result of the acetonitrile solution of **LH-3** (0.5 g L^-1^).


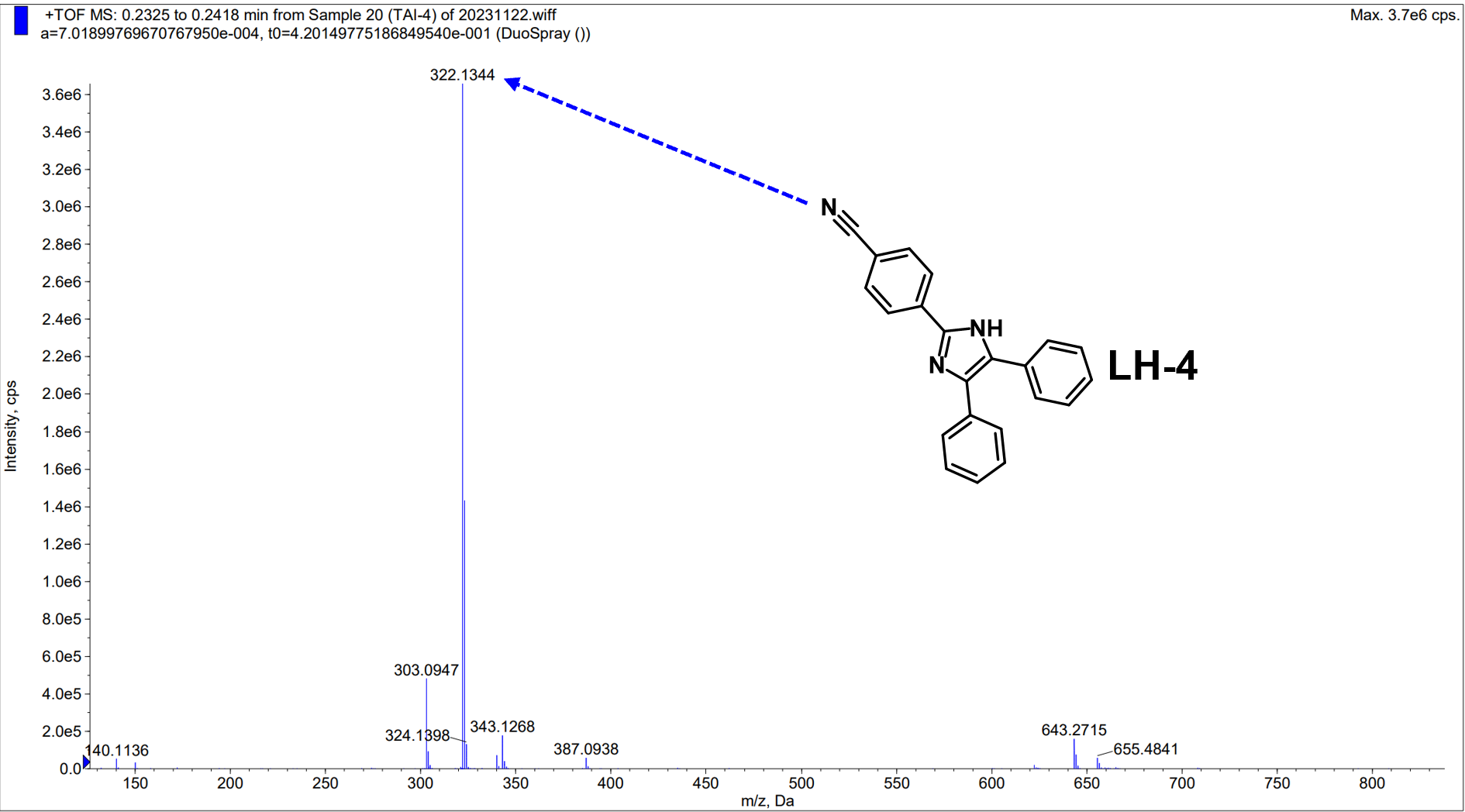


**Figure S80.** HRMS result of the acetonitrile solution of **LH-4** (0.5 g L^-1^).


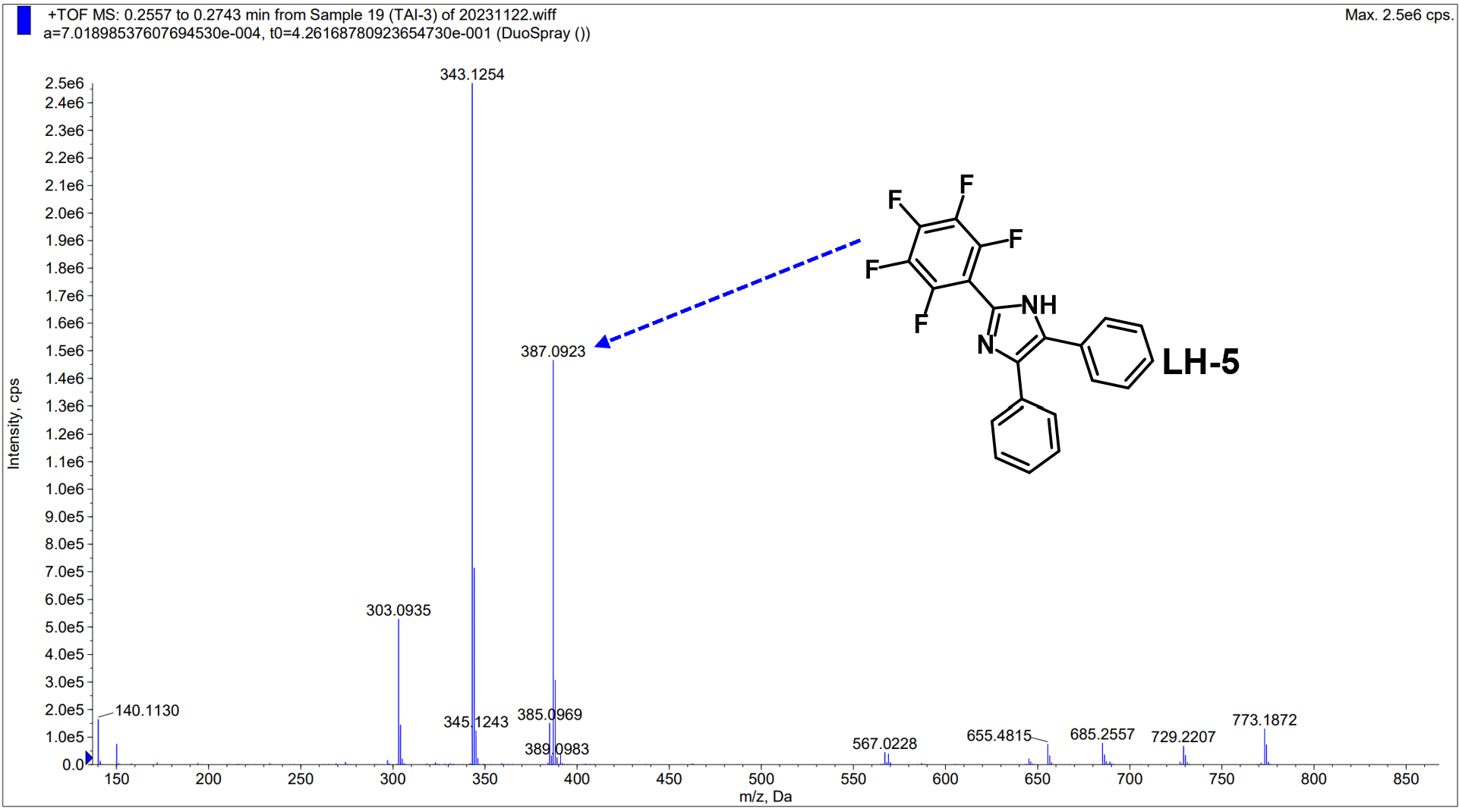


**Figure S81.** HRMS result of the acetonitrile solution of **LH-5** (0.5 g L^-1^).


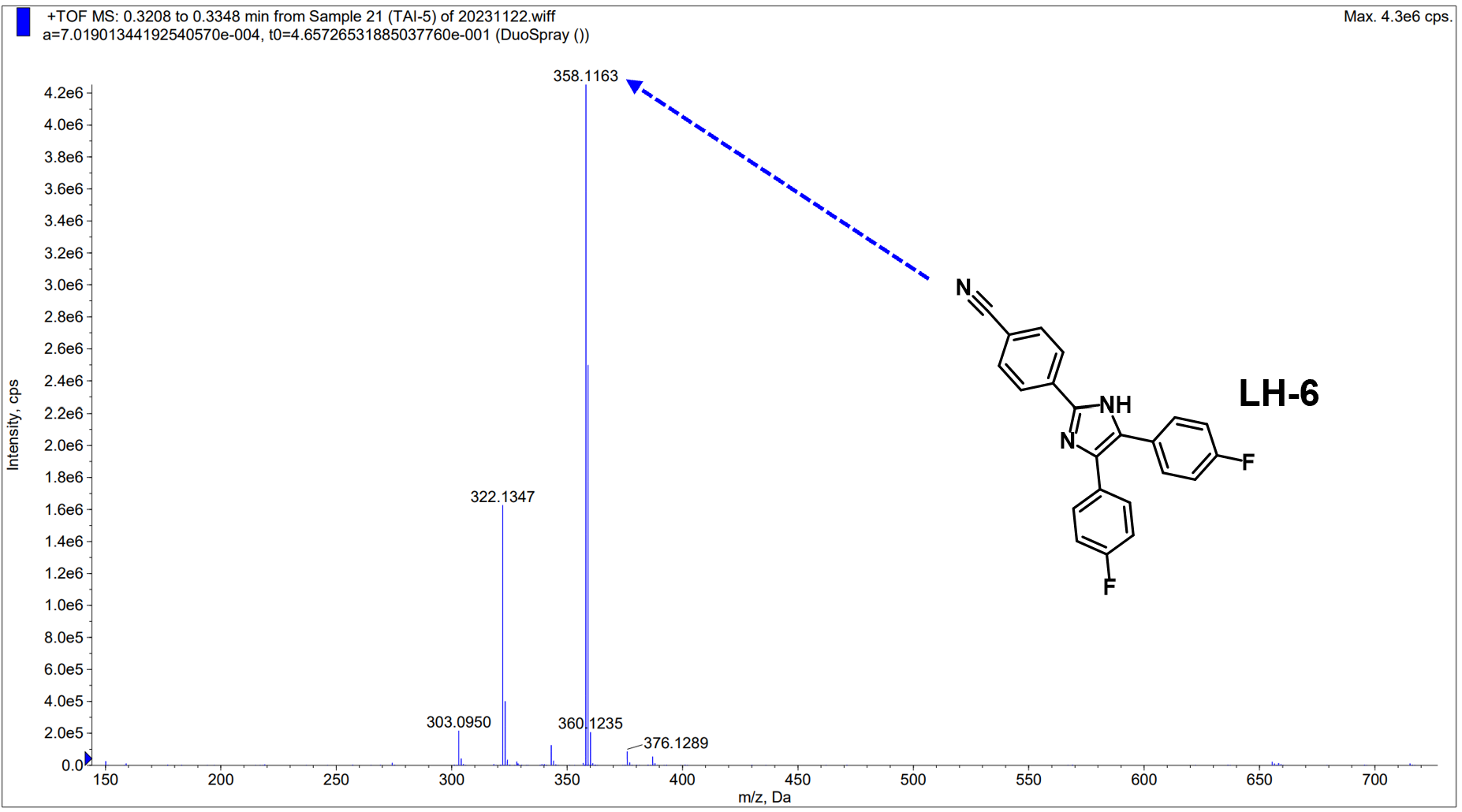


**Figure S82.** HRMS result of the acetonitrile solution of **LH-6** (0.5 g L^-1^).


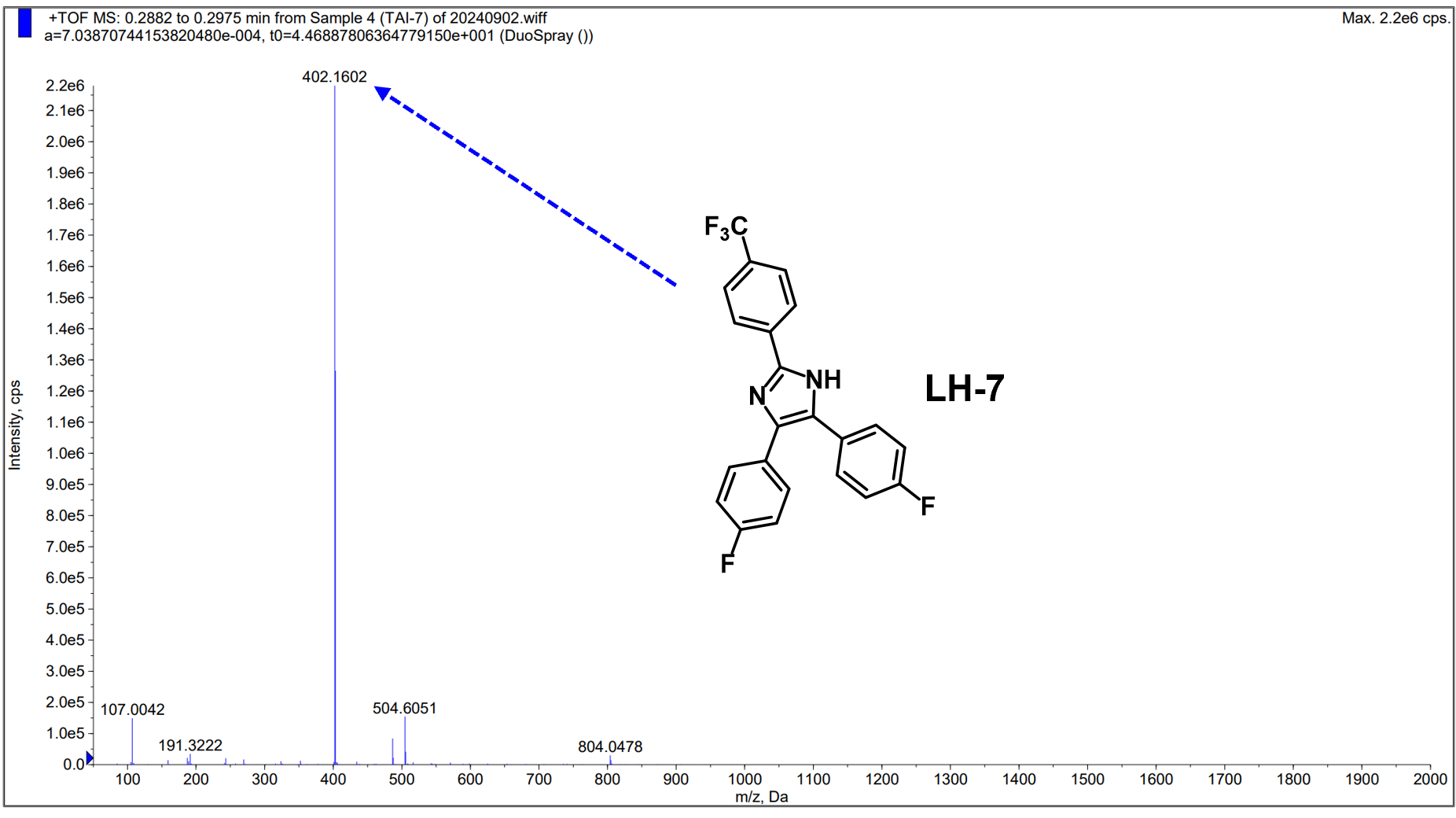


**Figure S83.** HRMS result of the acetonitrile solution of **LH-7** (0.5 g L^-1^).

# References

[1] T. Lu, F. Chen, *Journal of Theoretical and Computational Chemistry* 2012, *11*, 163-183.

[2] Y. Pang, S. Fan, Q. Wang, D. Oprych, A. Feilen, K. Reiner, D. Keil, Y. L. Slominsky, S. Popov, Y. Zou, B. Strehmel, *Angew. Chem. Int. Ed.* **2020**, *59*, 11440-11447.

[3] Y. Pang, A. Shiraishi, D. Keil, S. Popov, V. Strehmel, H. Jiao, J. S. Gutmann, Y. Zou, B. Strehmel, *Angew. Chem. Int. Ed.* **2020**, *60*, 1465-1473.

[4] X. He, Y. Shao, Y. Pang, S. Xiao, Y. Xin, Y. Zou, *Macromolecules* **2024**, *57*, 3148-3159.

[5] X. Sun, X. He, M. Yi, S. Fan, B. Xiang, B. Yuan, J. Zhu, P. Luo, Y. Zou, Y. Pang, *European Polymer Journal* **2024**, *211*, 113025.

[6] Y.-H. Li, Y.-C. Chen, *Polymer Chemistry* **2020**, *11*, 1504-1513.
